# Synthesis of benzoyl hydrazones having 4-hydroxy-3,5-dimethoxy phenyl ring, their biological activities, and molecular modeling studies on enzyme inhibition activities

**DOI:** 10.3906/kim-2107-7

**Published:** 2021-10-22

**Authors:** Bedriye Seda KURŞUN AKTAR, Yusuf SICAK, Gizem TATAR, Emine Elçin ORUÇ-EMRE

**Affiliations:** 1 Department of Property Protection and Security, Yapraklı Vocational School, Çankırı Karatekin University, Çankırı, Turkey; 2 Department of Hair Care and Beauty Services, Yeşilyurt Vocational School, Malatya Turgut Özal University, Malatya, Turkey; 3 Department of Herbal and Animal Production, Köyceğiz Vocational School, Muğla Sıtkı Koçman University, Muğla, Turkey; 4 Department of Biostatistics and Medical Informatics, Karadeniz Technical University, Trabzon, Turkey; 5 Department of Chemistry, Faculty of Arts and Sciences, Gaziantep University, Gaziantep, Turkey

**Keywords:** Benzoyl hydrazone, antioxidant activity, anticholinesterase enzyme inhibitory activity, tyrosinase enzyme inhibition activity, urease enzyme inhibition activity, molecular docking

## Abstract

Hydrazone compounds have high capacity in terms of antioxidant activity and enzyme inhibition activities such as anticholinesterase, tyrosinase, and urease. In this study, benzoyl hydrazones compounds **(7a–7m)** were synthesized starting from 3,5-dimethoxy-4-hydroxybenzaldehyde. Antioxidant activity of the synthesized compounds was evaluated. In the β-carotene-linoleic acid and ABTS cation radical scavenging activities, compounds **7j**, **7e**, and **7m** stood out as the most active compounds, respectively. In the anticholinesterase enzyme inhibition activity results, compound **7f** exhibited the best activity against AChE and BChE enzymes in the synthesis series. In addition, molecular docking analysis was performed to understand the inhibition mechanism of the synthesized compounds with target enzymes at the atomic level. In the light of biological activity and in silico studies, it has the potential to guide studies for the development of new drugs for Alzheimer disease in the future.

## 1. Introduction

The design, synthesis, characterization, and discovery of the effect of structure blocks of biologically active molecules in the wide spectrum has been a topic of interest for scientists in recent years. The emergence of drug resistance is a natural phenomenon, and studies regarding developing new preventatives have always been the subject of pharmaceutical research. This growing area of molecular synthetics has become an active area for research for biological and medical perspectives. Enzyme inhibition has been the subject of the research of scientists in the biomedical field for the last 20 years. Several inhibitors have been discovered and used to control various diseases. For instance, donepezil [1], rivastigmine [2], and galantamine [3] are used for acetylcholine inhibitory; urease inhibitors such as omeprazole, rabeprazole, and lansoprazole in the treatment of *Helicobacter pylori* infection [4]; kojic acid [5] and L-mimosine [6] for tyrosinase inhibitory.

One of the most important features of enzymes is that it shows selectivity against inhibitors, which can be a simple organic molecule or have a complex structure. The specificity of the inhibitor depends on the size, shape, and interaction forces that cause the inhibitor and the enzyme to be paired completely. These inhibitions block the activity of the enzyme under physiological conditions [7].

Alzheimer is a kind of dementia that causes problems in memory, thinking, and behaviors [8]. The brain has neural cells, which are connected to each other, in order to form connection networks. Scientists compare Alzheimer’s disease to system disruption of a factory where a part does not work properly. Although it is not certain where the problem originates from, it is thought that malfunctioning and back up failures in the system cause problems in other sections, as in a real factory. If the damage spreads, the cells could lose their ability to do their duties and die, causing irreversible damage to the brain. One of the reasons for the formation of Alzheimer’s disease is cholinergic effect hypothesis, which was suggested by Drachman and Leavitt in 1974 [9]. According to this hypothesis, cholinesterase inhibition, known as cholinergic effect, selectively degenerates neurons pathologically. According to the mechanism of this system, the decrease in the concentration of acetylcholine damages the neurons in the brain. Acetylcholinesterase affects the acetylcholine and hydrolyses it into acetic acid and choline. In this regard, it is needed to use agents, which inhibit the acetylcholinesterase enzyme.

Melanin is one of the factors which affect the colour of the skin and hair of humans. It is necessary to protect the human skin against radiation. Over accumulation of melanin causes pigmentation defects on the skin such as malezma, frackles, efelid, and senil lentijen [10]. Melanogenesis, which occurs in the melanocytes, is found in the basal layer of the epidermis which is controlled by tyrosinase [11]. Abnormal production of the melanin pigment causes aesthetical problems on the skin which is common in middle and old ages [12]. Melanin protects the skin against skin fotocancer, ultraviolet (UV) damage, and reactive oxygen species (ROS) by absorbing the UV rays and clearing the organism from toxic substances and drug residues [13]. Abnormal pigmentation of melanin is a serious health risk for humans. Tyrosinase inhibitors have clinical use against skin conditions related to melanin pigmentation and also used in cosmetics products thanks to their skin-whitening characteristic [14]. New tyrosinase inhibitors, whose side effects are minimized, are needed to prevent abnormal melanin pigmentation.

Urease is an enzyme, which catalyzes the hydrolysis of urea. It attracts attention due to its effects on the health and well-being of live organisms. The continuity of urease activity in human and animal cells may be the reason for some diseases and pathogenic infections. The urease enzyme which is produced by colonized helicobacter pylori protects the bacteria against the low pH value of the gastric fluid by forming CO_2_ and NH_3_ from urea. However, NH_3_ is not only toxic for gastric epithelium cells but also increases the effect of cytotoxins by adhesion between cells. In this regard, proton providing inhibitors and urease inhibitors are used to treat helicobacter pylori infection. Urease inhibitors also offer effective treatment for diseases caused by urease-dependent pathogenic microorganisms. For this reason, there is an ongoing increase in the number of research that focuses on the potential of natural products (in the form of extracts or pure compounds) as clinical urease inhibitors [4]. Clinical use of urease inhibitors such as phosphorodamidates, hydroxamic acid derivatives and imidazoles are limited because of their toxicity and low stability.

Free radicals are formed during normal metabolic activities or external factors such as environmental agents (pesticides, aromatic hydrocarbons, toxins, solvents, etc.), stress or radiation. Free radicals are short-lived reactive molecules, which have unpaired electron on their outer orbitals. The most important free radicals are superoxide radical (O_2_^•−^), hydroxyl radical (^•^OH), singlet oxygen (^1^O_2_), non-radical hydrogen peroxide (H_2_O_2_), and peroxynitrite (ONOO^−^), which are known as “reactive oxygen types (ROT). ROTs can go into reaction with lipids, nucleic acids, proteins, or carbohydrates easily in an organism. For this reason, they are held responsible for many illnesses such as aging [15], cardiovascular diseases [16], immune system diseases [17], cataract, diabetes [17], kidney & liver diseases [18].

Antioxidants are systems that eliminate the effects of free radicals. There are many enzymatic or non-enzymatic endogenous antioxidant defense mechanisms in the body to prevent the formation of and the damage caused by ROTs. In addition, some drugs, vitamins, and synthetic food antioxidants can also be commentated as exogenous antioxidants. They operate as protective antioxidants (enzymes, metal chelators) which delay or stop the formation of free radicals or chain-breaking antioxidants which prevent the progression of peroxidation.

Antioxidants that are synthesized by the body or taken from outside and that protect the biomolecules from oxidation damage have received more attention in recent years. Hydrazone is a special member of Schiff bases azomethine-containing [19]. It has pharmacological activity in a wide area such as antioxidant [19], analgesic [11], antiviral [12], antiepileptic [13], antimicrobial [14], antibacterial [15], anti-inflammatory [16] and anticancer [17].

The design and discovery of the effect of active molecules in a broad spectrum have become the focus of interest of scientists from a biological perspective in recent years. With the emergence of pathogenic resistance, research on the development of different inhibitors of a natural phenomenon in a new structure has always been the subject of pharmaceutical researchers. This requirement has become an active research area for the synthetic, biological, and medical perspectives at the molecular level.

In this study, it is aimed to synthesize new benzoyl hydrazones, which scavenge the effects of free radicals, are less toxic than the drugs on the market, and whose side effects are minimized. As a result of the literature studies, we examined the differences in biological activity caused by the changes in the positions and numbers of groups such as H, Br, Cl, F, NO_2_, CH_3_, OCH_3_, OCF_3_. In addition, the molecular docking study provided the opportunity to elucidate the binding mechanism of the designed compounds for target enzymes at the molecular level.

## 2. Materials and methods

**Figure 1:**
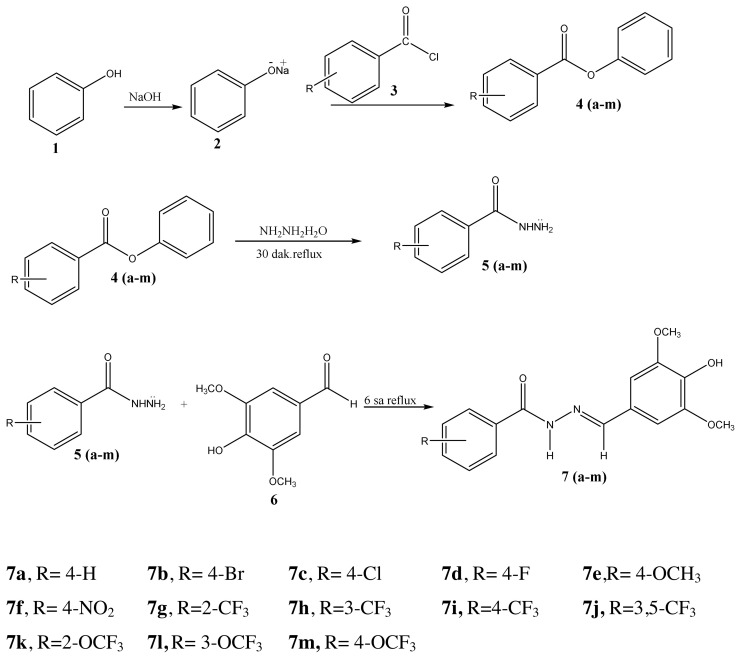
Synthetic route of target compounds (7a–7m).

The whole chemical substances were purchased from Merck and Sigma–Aldrich. The reaction monitoring of the synthesized compounds was checked by thin layer chromatography (TLC) using Merck silica gel 60 F254 plates. The melting point of the synthesized compounds was determined by the EZ-Melt MPA120 Automated Melting Point Apparatus (SRS) device. Infrared (IR) analyses were taken with the Perkin Elmer 1620 model FT-IR device in a system with ATR in the wavelength range of 4000-400 cm−1. Elemental analyses (CHNS) of compounds were determined using the Thermo Scientific Flash 2000 instrument. The ^1^H, ^13^C NMR, 2D NMR spectra of the original compounds were obtained at Çankırı Karatekin University with Agilent brand 600 MHz frequency, Premium Compact NMR device with 14.1 Tesla field strength and Bruker AVANCE III 400 MHz NMR Spectrometer at Giresun University. The FT-IR, Mass spectra, and NMR spectra of the compounds **(7a-m)** were supplied in the supplementary data. The synthetic route of target compounds **(7a-m)** is shown in Figure 1.

### 2.1. Chemistry

#### 2.1.1. Synthesis of substituted phenyl benzoate (4a-m)

0.1 mol of phenol is dissolved in 10% NaOH. 0.1 mol of substituted benzoyl chloride is added to the solution. After solid formation is observed, the reaction is continued to stir for another 1–2 h, and the solid formed is filtered off. The solid is washed with water and recrystallized from ethanol [27, 28].

#### 2.1.2. Synthesis of substituted benzoic acid hydrazide (5a-m)

0.05 mol of substituted benzaldehyde in 3 mL of methanol is mixed with 0.1 mol of 99% hydrazine hydrate, the rinsing is refluxed for 30 min, the mixture is allowed to cool once the product formed, and the precipitated solid is filtered off. The product obtained is purified by washing with plenty of water and crystallizing from ethanol [27, 28].

#### 2.1.3. *N*′-(4-hydroxy-3,5-dimethoxybenzylidene)substituted benzohydrazide (7a-m)

Substituted benzoic acid hydrazide dissolved in acetonitrile is dropped to 1 mol of 3,5-dimethoxy-4-hydroxybenzaldehyde dissolved in acetonitrile, and the reaction is refluxed for 6 h. The product formation terminated the reaction by controlling with TLC. The solid formed is filtered, dried, and purified with ethanol [27, 28].

##### *N*′-(4-hydroxy-3,5-dimethoxybenzylidene)benzohydrazide (7a)

Cream solid; yield: 45.59%, m.p. 217–218°C. FTIR *ν*_max_ (cm^−1^): 3529 (H-O), 3227 (N-H), 3072 (Ar-CH), 2968, 2936 (R-CH), 1643 (C=O), 1579 (C=N), 1558, 1516, 1495, 1458 (C=C). ^1^H NMR (600 MHz, DMSO-*d_6_*): δ 3.81 (s, 6H, H_6_), 6.97 (s, 2H, H_3_), 7.51 (t, 2H, *J_1_*=7.8, *J_1_*=7.2 Hz, H_12_), 7.57 (t, H, *J_1_*=7.2, *J_1_*=7.2 Hz, H_13_), 7.88 (d, 2H, *J*=7.2 Hz, H_11_), 8.32 (s, 1H, H_7_), 8.92 (s, 1H, H_5_) 11.72 (s, 1H, H_8_). ^13^C NMR (150 MHz, DMSO-*d_6_*): δ 56.48 (OCH_3_); 148.59 (C=N); 163.51 (C=O); 106.06, 125.02, 128.04, 128.94, 132.11, 134.09, 138.40 (Ar-C). Anal. Calcd for C_16_H_16_N_2_O_4_: C, 63.99; H, 5.37; N, 9.33%. Found: C, 63.50; H, 5.45; N, 9.40%. MS m/z: (300.11) 426.80 [M-H]^+^. [29].

##### 4-bromo-*N*′-(4-hydroxy-3,5-dimethoxybenzylidene)benzohydrazide (7b)

White solid; yield: 93.75%, m.p. 224–226°C. FTIR *ν*_max_ (cm^−1^): 3574 (H-O), 3502 (N-H), 3184 (Ar-CH), 2970 (R-CH), 1640 (C=O), 1583 (C=N), 1547, 1514, 1450 (C=C), 1123 (C-Br). ^1^H NMR (400 MHz, DMSO-*d_6_*): δ 3.83 (s, 6H, H_6_), 7.09 (s, 2H, H_3_), 7.75 (d, 2H, *J*=8 Hz, H_12_), 7.86 (d, 2H, *J*=8.4 Hz, H_11_), 7.87 (s, 1H, H_7_), 8.99 (s, 1H, H_5_) 11.81 (s, 1H, H_8_). ^13^C NMR (100 MHz, DMSO-*d_6_*): δ 56.51 (OCH_3_); 149.30 (C=N); 162.50 (C=O); 148.61, 138.53, 133.15, 131.96, 130.14, 124.90, 105.16 (Ar-C). Anal. Calcd for C_16_H_15_BrN_2_O_4_: C, 50.68; H, 3.99; N, 7.39%. Found: C, 50.94; H, 4.02; N, 7.45%. MS m/z: (378.02) 378.90 [M-H].^+^ [30].

##### 4-chloro-*N*′-(4-hydroxy-3,5-dimethoxybenzylidene)benzohydrazide (7c)

White solid; yield: 40.76%, m.p. 219–221°C. FTIR *ν*_max_ (cm^−1^): 3340 (H-O), 3168 (N-H), 2988 (Ar-CH), 2934 (R-CH), 1643 (C=O), 1585 (C=N), 1563, 1514, 1489 (C=C), 1088 (C-Cl). ^1^H NMR (600 MHz, DMSO-*d_6_*): δ 3.92 (s, 6H, H_6_), 5.80 (s, 1H, H_5_), 7.01 (s, 2H, H_3_), 7.46 (d, 2H, *J*=8.4 Hz, H_12_), 7.80 (d, 2H, *J*=8.4 Hz, H_11_), 8.20 (s, 1H, H_7_), 9.16 (s, 1H, H_8_). ^13^C NMR (150 MHz, DMSO-*d_6_*): δ 56.51 (OCH_3_); 148.61 (C=N); 165.32 (C=O); 137.26, 131.66, 129.97, 129.85, 129.17, 129.03, 124.91, 105.17 (Ar-C). Anal. Calcd for C_16_H_15_ClN_2_O_4_: C, 57.41; H, 4.52; N, 8.37%. Found: C, 57.93; H, 4.70; N, 8.69%. MS m/z: (334.07) 333.00 [M-H]^+^.[31].

##### 4-fluoro-*N*′-(4-hydroxy-3,5-dimethoxybenzylidene)benzohydrazide (7d)

Cream solid; yield: 79.66%, m.p. 223–225°C. FTIR *ν*_max_ (cm^−1^): 3424 (H-O), 3240 (N-H), 2960 (Ar-CH), 2850 (R-CH), 1636 (C=O), 1583 (C=N), 1557, 1504, 1447 (C=C), 1219 (C-F). ^1^H NMR (600 MHz, DMSO-*d_6_*): δ 3.87 (s, 6H, H_6_), 5.79 (s, 1H, H_5_), 7.01 (s, 2H, H_3_), 7.15 (t, 2H, H_12_), 7.87 (s, 2H, H_11_), 8.21 (s, 1H, H_7_), 9.25 (s, 1H, H_8_). ^13^C NMR (150 MHz, DMSO-*d_6_*): δ 56.51 (OCH_3_); 148.61 (C=N); 165.77 (C=O); 163.30, 162.41, 149.07, 138.49, 130.68, 124.97, 116.01, 105.16 (Ar-C). Anal. Calcd for C_16_H_15_FN_2_O_4_: C, 60.37; H, 4.75; N, 8.80%. Found: C, 60.78; H, 4.99; N, 8.93%. MS m/z: (318.10) 317.00 [M-H]^+^.[32].

##### *N*′-(4-hydroxy-3,5-dimethoxybenzylidene)-4-methoxybenzohydrazide (7e)

Cream solid; yield: 95.8%, m.p. 231–232°C. FTIR *ν*_max_ (cm^−1^): 3439 (H-O), 3254 (N-H), 3061 (Ar-CH), 2933 (R-CH), 1620 (C=O), 1585 (C=N), 1560, 1507, 1463 (C=C). ^1^H NMR (400 MHz, DMSO-*d_6_*): δ 3.82 (s, 6H, H_6,_), 3.84 (s, 3H, H_14_), 6.99(s, 2H, H_3_), 7.07 (d, 2H, *J*=8,4 Hz, H_12_), 7.91 (d, 2H, *J*=8.8 Hz, H_11_), 8.34 (s, 1H, H_7_), 8.94 (s, 1H, H_5_) 11.63 (s, 1H, H_8_). ^13^C NMR (100 MHz, DMSO-*d_6_*): δ 55.88 (C_6_), 56.47 (C_14_), 105.01 (C_3_), 114.18 (C_12_), 125.16 (C_4_), 126.11 (C_10_), 129.96 (C_11_), 138.31 (C_1_), 148.40 (C_2_), 148.60 (C_7_), 162.40 (C_9_), 162.92 (C_13_). COSY NMR (100 MHz) (DMSO-*d_6_*/TMS): δ H_11_-H_12_. HETCOR NMR (100 MHz) (DMSO-*d_6_*/TMS): δ 55.88 - 3.82 (C_6_-H_6_); 56.45 - 3.84 (C_14_-H_14_); 105.01 - 6.99 (C_3_-H_3_); 114.18 - 7.07 (C_12_-H_12_); 129.96 - 7.91 (C_11_-H_11_); 148.40 - 8.34 (C_7_-H_7_). HMBC NMR (100 MHz) (DMSO-*d_6_*/TMS): δ C_1_-H_2_,_6_; C_10_-H_12_; C_7_-H_3,5_,_6_; C_9_-H_11_; C_13_-H_14_. Anal. Calcd for C_17_H_18_N_2_O_5_: C, 61.81; H, 5.49; N, 8.48%. Found: C, 61.90; H, 5.67; N, 8.68%. MS m/z: (330.12) 328.90 [M-H]^+^. [33].

##### *N*′-(4-hydroxy-3,5-dimethoxybenzylidene)-4-nitrobenzohydrazide (7f)

Yellow solid; yield: 70.16%, m.p. 228–230°C. FTIR *ν*_max_ (cm^−1^): 3523 (H-O), 3471 (N–H), 3182, 2982 (Ar-CH), 2965, 2849 (R-CH), 1643 (C=O), 1577 (C=N), 1516, 1454, 1422 (C=C). ^1^H NMR (600 MHz, DMSO-*d_6_*): δ 3.82 (s, 6H, H_6_), 6.93 (s, 2H, H_3_), 8.12 (d, 2H, *J*=8.4 Hz, H_11_), 8.33 (s, 1H, H_7_), 8.36 (d, 2H, *J*=8.4 Hz, H_12_), 8.97 (s, 1H, H_5_), 12.00 (s, 1H, H_8_). ^13^C NMR (150 MHz, DMSO-*d_6_*): δ 56.48 (OCH_3_); 148.57 (C=N); 161.72(C=O); 149.98, 149.60, 139.77, 138.65, 129.55, 124.66, 124.09, 105.21 (Ar-C). Anal. Calcd for C_16_H_15_N_3_O_6_: C, 55.65; H, 4.38; N, 12.17%. Found: C, 55.78; H, 4.47; N, 12.60%. MS m/z: (345.10) 426.80 [M-H]^+^. [32].

##### *N*′-(4-hydroxy-3,5-dimethoxybenzylidene)-2-(trifluoromethyl)benzohydrazide (7g)

Cream solid; yield: 91%, m.p. 225–227°C. FTIR *ν*_max_ (cm^−1^): 3601 (H-O), 3579 (N–H), 3186, 2972 (Ar-CH), 2972 (R-CH), 1655 (C=O), 1580 (C=N), 1546, 1514, 1463 (C=C), 1228 (C-F). ^1^H NMR (400 MHz, DMSO-*d_6_*): δ 3.62, 3.83 (s, 6H, H_6_), 7.00, 6.61 (s, 2H, H_3_), 7.53, 7.87(d, 1H, *J*=7.6 Hz, H_15_), 7.71-7.65 (m, 2H, H_13_,_14_), 7.74 (d, 1H, *J*=7.6 Hz, H_12_), 8.14, 7.85 (s, 1H, H_7_), 8.95, 8.80 (s, 1H, H_5_), 11.94, 11.83 (s, 1H, H_8_). ^13^C NMR (100 MHz, DMSO-*d_6_*): δ 56.24, 56.58, 104.84, 105.39, 122.83, 124.74, 124.86, 125.55, 125.95, 126.39, 126.70, 127.01, 128.88, 129.42, 129.67, 130.69, 132.44, 133.66, 135.25, 135.63, 138.18, 138.71, 144.13, 148.47, 148.68, 149.18, 163.35, 169.55. Anal. Calcd for C_17_H_15_F_3_N_2_O_4_: C, 55.44; H, 4.11; N, 7.61%. Found: C, 55.65; H, 4.23; N, 7.89%. MS m/z: (368.10) 367.00 [M-H]^+^.[34].

##### *N*′-(4-hydroxy-3,5-dimethoxybenzylidene)-3-(trifluoromethyl)benzohydrazide (7h)

Cream solid; yield: 80%, m.p. 226–228°C. FTIR *ν*_max_ (cm^−1^): 3627 (H-O), 3544 (N–H), 3425 (Ar-CH), 2988 (R-CH), 1642 (C=O), 1578 (C=N), 1516, 1490, 1469 (C=C), 1207 (C-F). ^1^H NMR (400 MHz, DMSO-*d_6_*): δ 3.84 (s, 6H, H_6_), 7.05 (s, 2H, H_3_), 7.79 (t, 1H, *J_1_*=7.6, *J_2_*=7.6 Hz, H_14_), 7.98 (d, 1H, *J*=8.4 Hz, H_13_), 8.21 (s, 1H, H_15_), 8.24 (d, 1H, *J*=8.4 Hz, H_11_), 8.35(s, 1H, H_7_), 8.98(s, 1H, H_5_), 11.93 (s, 1H, H_8_). ^13^C NMR (100 MHz, DMSO-*d_6_*): δ 56.54 (OCH_3_); 148.64 (C=N); 161.94 (C=O); 149.73, 138.67, 135.02, 132.23, 130.30, 129.85, 129.53, 128.63, 124.81, 124.56, 105.29 (Ar-C). Anal. Calcd for C_17_H_15_F_3_N_2_O_4_: C, 55.44; H, 4.11; N, 7.61%. Found: C, 55.60; H, 4.27; N, 7.69%. MS m/z: (368.10) 367.00 [M-H]^+^. [34].

##### *N*′-(4-hydroxy-3,5-dimethoxybenzylidene)-4-(trifluoromethyl)benzohydrazide (7i)

White solid; yield: 60.39%, m.p. 199–201°C. FTIR *ν*_max_ (cm^−1^): 3565 (H-O), 3501 (N-H), 3186 (Ar-CH), 2923 (R-CH), 1642 (C=O), 1583 (C=N), 1547, 1514, 1457, 1423 (C=C), 1207 (C-F). ^1^H NMR (400 MHz, DMSO-*d_6_*): δ 3.83 (s, 6H, H_6_), 7.01 (s, 2H, H_3_), 7.92 (d, 2H, *J*=8.00 Hz, H_12_), 8.10 (d, 2H, *J*=7.60 Hz, H_11_), 8.34 (s, 1H, H_7_), 8.99 (s, 1H, H_5_), 11.94 (s, 1H, H_8_). ^13^C NMR (100 MHz, DMSO-*d_6_*): δ 56.99 (OCH_3_); 148.62 (C=N); 162.98(C=O); 159.07, 150.11, 138.52, 130.54, 125.96, 123.91, 122.26, 105.69 (Ar-C). Anal. Calcd for C_17_H_15_F_3_N_2_O_4_: C, 55.44; H, 4.11; N, 7.61 %. Found: C, 55.56; H, 4.37; N, 7.71%. MS m/z: (368.10) 426.80 [M-H]^+^.

##### *N*′-(4-hydroxy-3,5-dimethoxybenzylidene)-3,5-bis-(trifluoromethyl)benzohydrazide (7j)

Yellow solid; yield: 65%, m.p. 207–209°C. FTIR *ν*_max_ (cm^−1^): 3641 (H-O), 3503 (N–H), 3220 (Ar-CH), 2970 (R-CH), 1647 (C=O), 1588 (C=N), 1515, 1461, 1424 (C=C), 1212 (C-F). ^1^H NMR (400 MHz, DMSO-*d_6_*): δ 3.84 (s, 6H, H_6_), 7.03 (s, 2H, H_3_), 8.35 (s, 1H, H_13_), 8.38 (s, 2H, H_11_), 8.55 (s, 1H, H_7_), 9.01 (s, 1H, H_5_), 12.10 (s, 1H, H_8_). ^13^C NMR (100 MHz, DMSO-*d_6_*): δ 56.54 (OCH_3_); 148.64 (C=N); 160.46 (C=O); 150.37, 138.86, 136.39, 131.17, 130.84, 128.84, 124.58, 122.21, 105.38 (Ar-C). Anal. Calcd for C_18_H_14_F_6_N_2_O_4_: C, 49.55; H, 3.23; N, 6.42 %. Found: C, 49.70; H, 4.40; N, 6.51%. MS m/z: (436.09) 435.00 [M-H]^+^.

##### *N*′-(4-hydroxy-3,5-dimethoxybenzylidene)-2-(trifluoromethoxy)benzohydrazide (7k)

Cream solid; yield: 37.67%, m.p. 214–217°C. FTIR *ν*_max_ (cm^−1^): 3674 (H-O), 3410 (N-H), 3210 (Ar-CH), 2987, 2900 (R-CH), 1641 (C=O), 1584 (C=N), 1545, 1517, 1447 (C=C), 1211 (C-F). ^1^H NMR (400 MHz, DMSO-*d_6_*): δ 3.65, 3.83 (s, 6H, H_6_), 7.01, 7.17 (s, 2H, H_3_), 7.69-7.44 (m, 3H, H_13_,_14,15_), 8.17, 7.92 (d, 1H, *J*=8.00 Hz, H_12_), 8.83, 8.60 (s, 1H, H_7_), 9.11, 8.98 (s, 1H, H_5_),11.93, 11.83 (s, 1H, H_8_). ^13^C NMR (100 MHz, DMSO-*d_6_*): δ 56.14, 56.46 (OCH_3_); 148.40, 148.59 (C=N); 161.39, 161.14 (C=O); 167.68, 149.01, 145.63, 145.21, 144.32, 139.34, 138.54, 138.03, 132.45, 131.47, 130.43, 130.21, 130.00, 128.18, 127.32, 124.68, 122.07, 121.75, 119.98, 106.24, 105.17, 104.62 (Ar-C). Anal. Calcd for C_17_H_15_F_3_N_2_O_5_: C, 53.13; H, 3.93; N, 7.29%. Found: C, 53.33; H, 4.00; N, 7.45 %. MS m/z: (384.09) 382.90 [M-H]^+^.

##### *N*′-(4-hydroxy-3,5-dimethoxybenzylidene)-3-(trifluoromethoxy)benzohydrazide (7l)

White solid; yield: 79.62 %, m.p. 209–211 °C. FTIR *ν*_max_ (cm^−1^): 3671 (H-O), 3299 (N–H), 3169 (Ar-CH), 2988, 2900 (R-CH), 1637 (C=O), 1584 (C=N), 1524, 1483 (C=C), 1210 (C-F). ^1^H NMR (400 MHz, DMSO-*d_6_*): δ 3.74 (s, 1H, H_5_), 3.82 (s, 6H, H_6_), 6.84 (s, 1H, H_8_), 6.99 (s, 2H, H_3_), 7.61 (d, 1H, *J*=8,4 Hz, H_13_), 7.68 (t, 1H, H_14_), 7.87 (s, 1H, H_11_), 7.97 (d, 1H, *J*=8 Hz, H_15_), 8.35 (s, 1H, H_7_). ^13^C NMR (100 MHz, DMSO-*d_6_*): δ 56.47 (OCH_3_); 149.79 (C=N); 161.70 (C=O); 105.31, 120.55, 121.89, 124.53, 127.19, 131.16, 136.39, 139.20, 142.52, 145.33, 148.73 (Ar-C). Anal. Calcd for C_17_H_15_F_3_N_2_O_5_: C, 53.13; H, 3.93; N, 7.29 %. Found: C, 53.36; H, 4.03; N, 7.35%. MS m/z: (384.09) 382.90 [M-H]^+^.

##### *N*′-(4-hydroxy-3,5-dimethoxybenzylidene)-4-(trifluoromethoxy)benzohydrazide (7m)

White solid; yield: 62.80 %, m.p. 205–206 °C. FTIR *ν*_max_ (cm^−1^): 3409 (H-O), 3213 (N-H), 2923 (Ar-CH), 2851 (R-CH), 1641 (C=O), 1584 (C=N), 1544, 1505, 1517, 1468, 1427 (C=C), 1210 (C-F). ^1^H NMR (600 MHz, DMSO-*d_6_*): δ 3.94 (s, 6H, H_6_), 5.81 (s, 1H, H_5_), 7.02 (s, 2H, H_3_), 7.32 (d, 2H, *J*=8.4 Hz, H_12_), 7.90 (d, 2H, *J*=8.4 Hz, H_11_), 8.21 (s, 1H, H_7_), 9.14 (s, 1H, H_8_). ^13^C NMR (150 MHz, DMSO-*d_6_*): δ 56.50 (OCH_3_); 148.61 (C=N); 162.28 (C=O); 150.93, 149.36, 138.54, 133.25, 130.42, 124.88, 121.28, 119.17, 105.17 (Ar-C). Anal. Calcd for C_17_H_15_F_3_N_2_O_5_: C, 53.13; H, 3.93; N, 7.29%. Found: C, 53.27; H, 3.99; N, 7.40%. MS m/z: (384.09) 382.90 [M-H]^+^. [34].

### 2.2. Biological Assays

#### 2.2.1. Antioxidant activity

For antioxidant activity, all synthesized compounds were dissolved in DMSO, and stock solutions were prepared at concentrations of 5, 25, 50, and 100 μM/mL. DMSO was used as a control.

##### 2.2.1.1. β-carotene-linoleic acid discoloration activity method

Total antioxidant activity was determined by the method based on the measurement of conjugated diene hydroperoxides by oxidation of linoleic acid, which expresses the β-carotene-linoleic acid color bleaching [35]. By adding 160 μL of β-carotene solution to 40 μL from stock solutions in the different concentrations, the initial absorbance at 490 nm wavelength was measured in the spectrophotometer as soon as the emulsified solutions were transferred to the microplates. After the first measurement, the microplates were incubated at 45°C, and incubation was continued until the control β-carotene color disappeared (approximately 120 min every half hour). β-carotene lightening ratio (R) was calculated according to the formula below:


$$R=\left(\text{ln }\left(\frac{a}{b}\right)\right)\;t$$

ln: natural logarithm, a: initial absorbance, b: final absorbance at the end of incubation, t: incubation time (min)
Antioxidant activity (AA) was calculated according to the following formula:

$$\text{AA }(\%\;\text{Inhibition})=\frac{R_{Control}-R_{Sample}}{R_{control}}\times 100$$

R_Control_ is the lightning speed of the control, while R_Sample_ is the lightning speed of the sample.

##### 2.2.1.2. DPPH free radical scavenging activity method

Free radical scavenging of synthetic substances were determined by using DPPH free radical [36]. A total of 160 μL of DPPH solution was added to 40 μL stock sample solutions at different concentrations. After the samples were incubated for 30 min at 25 °C, absorbance was measured in a spectrophotometer at 517 nm wavelength. Free radical scavenging activity was calculated according to the formula:


$$\text{DPPH scavenging activities }(\%\;\text{Inhibition})=\frac{A_{Control}-A_{sample}}{A_{Control}}\times 100$$

A_Control_, absorbance of control; A_sample_ is the absorbance of the sample.

##### 2.2.1.3. ABTS cation radical scavenging activity method

The cation radical removal activities of the synthesis substances were determined by using the ABTS cation radical [37]. After 160 μL of ABTS solution was added to 40 μL stock sample solutions at different concentrations, the absorbance of the samples was measured at 734 nm wavelength spectrophotometer.


$$\text{ABTS scavenging activities }(\%\;\text{Inhibition})=\frac{A_{Control}-A_{sample}}{A_{Control}}\times 100$$

##### 2.2.1.4. CUPRAC activity method (Cuprac (II) reducing power)

The Cu (II) reducing power of the synthesis substances was determined according to the method of Apak et al., (2004) [38]. After the addition of 40 μL of stock solutions of samples prepared in different concentrations, 60 μL of pH = 7.0 ammonium acetate buffer and finally 100 μL of neocuprin-Cu (II) reagent, the absorbance of the solutions kept at 25 °C for 1 h was measured at 450 nm wavelength spectrophotometer [39].

#### 2.2.2. Enzyme inhibition activities

##### 2.2.2.1. Anticholinesterase inhibitory activity

The anticholinesterase activity, AChE and BChE inhibition activities of the synthetic substances were determined according to the Ellman method [40]. Galantamine and DMSO were used as positive controls for both tests.

For AChE inhibition activity, 160 μL of 0.1 M pH = 8.0 phosphate buffer, 10 μL of different concentrations of stock solutions and 10 μL of AChE solution obtained from eel were added to each well of 96-well microplates, and the first measurement was taken and after measurement incubated for 15 min at 25°C. At the end of the incubation, as soon as 10 μL of DTNB solution and 10 μL of AcI substrate were added to each well, kinetic absorbance was measured for 10 min at a wavelength of 412 nm.

In BChE inhibition activity, enzyme, substrate and activity measurement, butyrylcholinesterase, butyrylthiocholine chloride and 5,5′-dithiobis-(2-nitrobenzoic acid) (DTNB) obtained from horse serum were used, respectively. 160 μL of 0.1M pH=8.0 phosphate buffer, 10 μL of different concentrations of stock solutions and 10 μL of BChE solution obtained from horse serum were added to each well of 96-well microplates, and the first measurement was taken and incubated for 15 min at room conditions. At the end of the incubation, as soon as 10 μL of DTNB solution and 10 μL of BuCI substrate were added to each well, kinetic absorbance was measured for 10 min at a wavelength of 412 nm.

##### 2.2.2.2. Tyrosinase inhibitory activity

The tyrosinase enzyme inhibition activity of the synthesized compounds was determined using by taking measurements at 475 nm wavelength in the spectrophotometer using tyrosinase fungus as enzyme, L-DOPA as substrate, and phosphate buffer as buffer [41]. When using tyrosinase mushroom as enzyme; L-DOPA was used as the substrate. A total of 150 μL of 0.005 M pH = 6.8 phosphate buffer, 10 μL of the stock solution in the different concentrations, 20 μL of tyrosinase enzyme prepared from tyrosinase fungus were added to the microplates with this order, and then mixed for 3 min, and incubated at 37 °C for 10 min. At the end of the incubation, as soon as 20 μL of mikrop-DOPA solution was added to the microplate, kinetic absorbances were measured at 37 °C for 10 min at a wavelength of 475 nm.

##### 2.2.2.3. Urease inhibitory activity

The urease inhibition activity of the synthesized products was determined spectroscopically by measuring the ammonia formed as a result of the reaction according to the indophenol method [42]. Deionized water was used as a control. The urease enzyme was prepared in 25 μL from the 0.01 M pH = 8.2 phosphate buffer to the 10 μL stock solutions in different concentrations and then 50 μL of urea solution in 0.01 M pH = 8.2 phosphate buffer as a substrate was added and incubated at 25 °C for 15 min. At the end of the incubation, 30 min after the addition of 45 μL of phenol reagent and 70 μL of alkaline reagent, kinetic absorbance measurements of the samples were taken in the spectrophotometer at a wavelength of 630 nm.

### 2.3. Statistical analysis

The results of antioxidant, anticholinesterase, tyrosinase inhibition and urease inhibition activities are given as the mean of three parallel measurements ± standard error. Results were found within 95% confidence limits according to Student-t test. There was no significant difference between parallel measurements. Linear regression analysis using the least squares method was performed by evaluating the slope, intercept, and correlation coefficients.

### 2.4. Molecular docking

The molecular docking approach is a structure-based drug design method that requires three-dimensional (3D) structure information of the target protein and ligand to model the binding interaction between the ligand and the protein in the atomic level. In accordance with this, the 3D crystal structures of target enzymes of AChE, BChE, Tyrosinase, and Urease were accessed via the protein data bank (PDB) web site (http://www.rcsb.org/pdb) as PDB ID: 4EY6, 6QAA, 2Y9X, and 3LA4, respectively. Water and ion molecules were removed from this selected crystal structure and the missing hydrogen atoms and atomic charges were structured through the APBS-PDB2PQR program [43]. Synthesized compounds were drawn, and the 3D structures of these compounds were converted to .pdb format with the BIOVIA Discovery Studio 2020 Client program [44]. After the structure preparation processes were completed for the target proteins and synthesized compounds, molecular docking was performed using the AutoDock 4.2 [45] with Lamanckian genetic algorithm and 100 run steps.

## 3. Results and discussion

### 3.1. Chemistry

**Figure 2:**
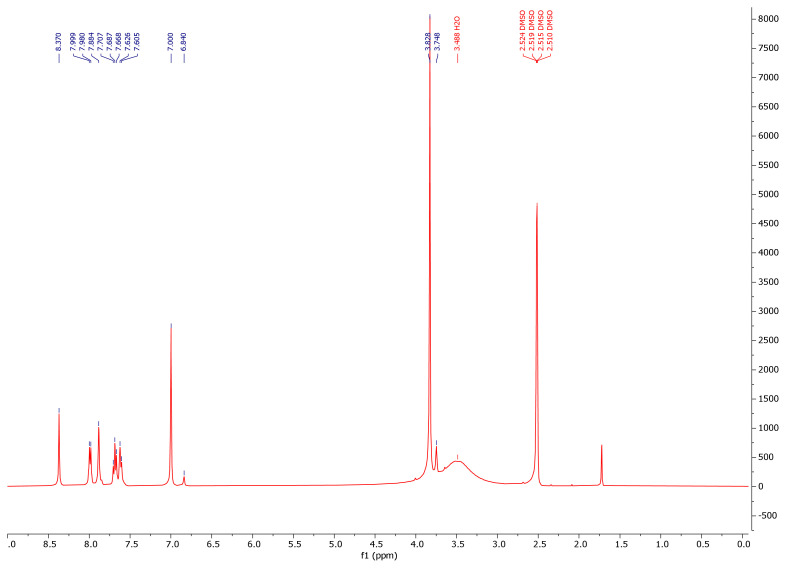
^1^H NMR spectrum of compound **7l**.

**Figure 3:**
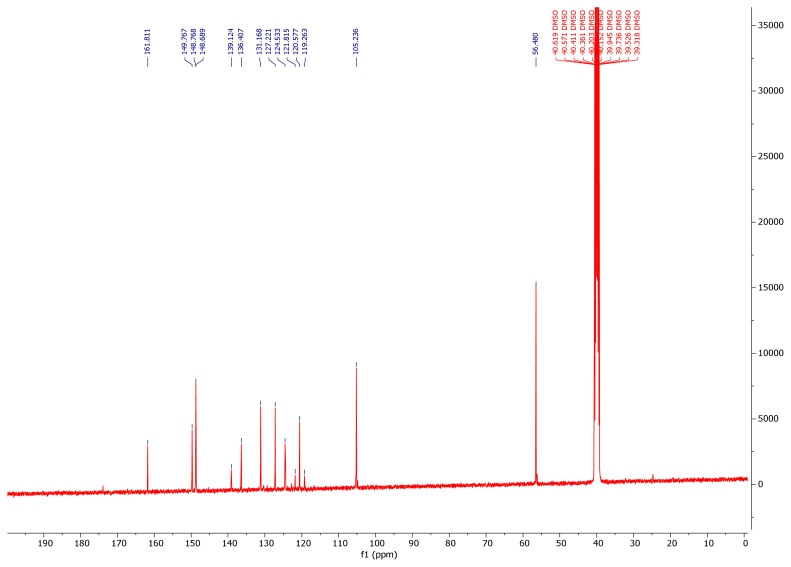
^13^C NMR spectrum of compound **7l**.

**Figure 4:**
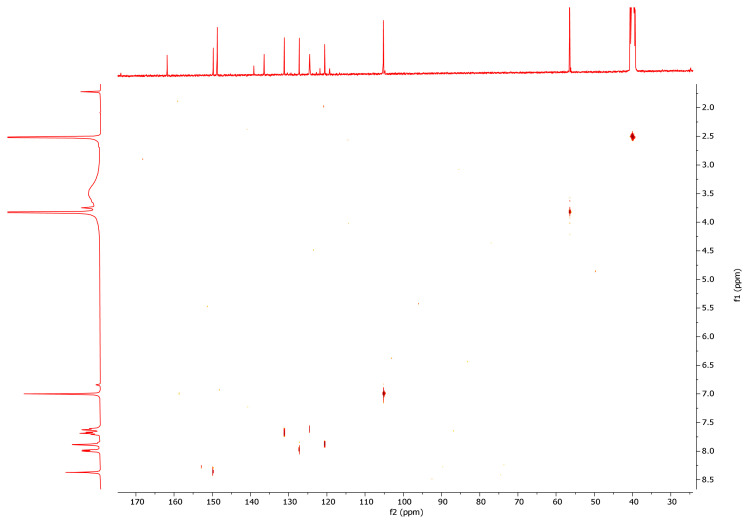
HETCOR NMR spectrum of compound **7l**.

**Figure 5:**
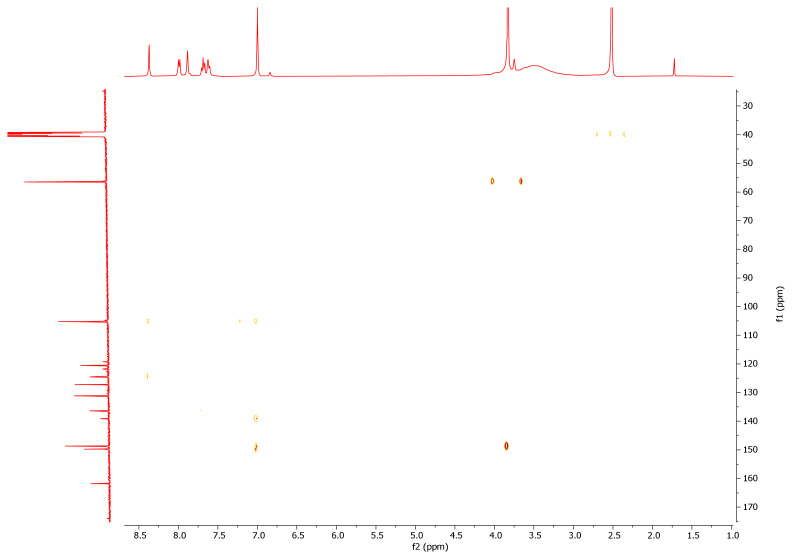
HMBC NMR spectrum of compound **7l**.

While the -NH2 protons of the hydrazide compounds were observed at 4–5 ppm, it was determined that this peak disappeared in the synthesized benzoyl hydrazone compounds and an NH peak was formed in the 12.00–8.35 ppm range. OCH_3_ protons in the compounds **7a–7m** were in the range of 3.94–3.62 ppm; the -OH proton was in the range of 9.11–5.79 ppm (compound **7l** only came out at 3.74); the -CH proton of the aldehyde was detected in the range of 8.83–7.87 ppm. Chlorine and fluorine compounds attached to the aromatic ring shift the neighbouring proton to the upper area because they gave their electron density to the ring, while the electron-withdrawing group NO_2_ shifts the adjacent proton to the lower area. Since the F atom has ½ spin number, it was determined that the peak belonging to the proton in its neighbor is a triplet peak in (Figure 2) [37–39]. In ^13^C NMR spectra, the most important proof of synthesis of benzoyl hydrazone compounds was the resonance of the carbon peak of azomethine group (C=N) in the range of 148.40–149.79 ppm. The carbon peak of the group (OCH_3_) of compounds (**7a–7m**) was in the range of 56.99–56.14; the carbon peak of the (C=O) group was in the range of 160.46–165.32; while the carbon peak of the (Ar-C) group was detected in the range of 167.68–104.62 in (Figure 3) [46–48]. The 2D NMR value of compound **7l** was given in (Table 1, Figure 4, Figure 5).

### 3.2. Antioxidant activity evaluation

The IC_50_ (μM/mL) values of the antioxidant activity of thirteen benzoyl hydrazones **7a–7m** were given in Table 2. In the β-carotene-linoleic acid and ABTS cation radical scavenging activities, ccompounds**7j**, **7e**, and **7m** stand out as the most active compounds, respectively. According to the DPPH radical scavenging activity assay data, all tested compounds exhibited much better activity than BHT, which was used as the positive standard of the assay. In addition, compounds **7e**, **7j**, and **7m** were the most active compounds in the assay and were also determined to be active from α-TOC. In the CUPRAC activity results, all synthesized compounds (except **7b**, **7h** and **7l**), showed much better activity than the α-TOC used as the standard of the assay. Generally, in the antioxidant activity tests of benzoyl hydrazones **7a–7m**, compounds **7e**, **7j** and **7m** were detected having the most active compounds in β-carotene-linoleic acid, DPPH free radical scavenging, ABTS cation radical scavenging and CUPRAC capacity activity.

### 3.3. Evaluation of enzyme inhibition activities

The IC_50_ values of anticholinesterase, tyrosinase and urease enzyme inhibition activities of thirteen benzoyl hydrazones **7a–7m** were given in Table 3. In the anticholinesterase enzyme inhibition activity results, compound **7f** exhibited the best activity against AChE and BChE enzymes in the synthesis series. According to the tyrosinase enzyme inhibition activity test results of benzoyl hydrazones **7a–7m**, compounds **7e** and **7f** were the most active compounds in the synthesized series. In the urease enzyme inhibition activity study, it was found that compound **7e** exhibited the best activity in the synthesis series.

### 3.4. Molecular docking studies

The molecular docking approach allows us to elucidate the interaction between small molecules in the binding site of target biomolecules (enzyme, protein, nucleic acids) at the atomic level, as well as to explain basic biochemical processes. Intermolecular interactions such as hydrogen bonding and hydrophobic interactions play an important role in stabilizing energetically preferred ligands in the binding site of target biomolecules, and contribute to enhancing binding affinity and drug efficacy. Thanks to this analysis, we explained the interactions of these synthesized compounds towards target proteins that will contribute to their biological activities at the molecular level.

**Figure 6:**
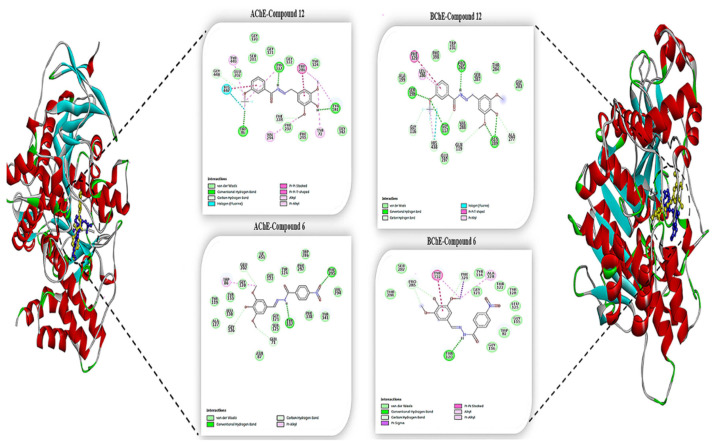
The 2D analysis of compounds **7l** and **7f** showing high efficiency against AChE, BChE.

Acetylcholine (Ach) in the brain is mainly hydrolyzed by cholinesterase (ChE), and thus ChE inhibition has proven to be an effective route for AD treatment. There are two groups of ChE in the nervous system: acetylcholinesterase (AChE) and butyrylcholinesterase (BChE) [49]. For both enzymes, hydrolysis is located near the bottom of a 20 Å deep active site gorge. The catalytic triad of serine, histidine and glutamate residues within the active site gorge were identified as important in facilitating efficient catalysis and inhibitor binding. The human AChE consists of the catalytic triad of Glu334, His447 and Ser203 [50–51] and BChE consists of the catalytic triad of Ser198, His438 and Glu325 [52]. As a result of molecular docking simulation, **7k** showed the best inhibition on AChE and BChE (Binding Energy: −8.76 kcal/mol, −7.01 kcal/mol, respectively). This compound formed five hydrogen bonds with Trp86, Tyr337, Tyr341, Gly448, Phe338 residues, as well as seven pi-alkyl interactions with Tyr72, Trp86, Trp286, Tyr337, Phe338, Tyr341, Tyr449 residues, especially it interacted halogen bond with His447 which belong to the AChE active site gorge flap. Besides, this compound exhibited seven hydrogen bond interactions Glu117, Asn289, Pro285, Gly116, Ala277, and Gly119 residues, particularly it established the hydrogen and halogen bond interaction with Gly116 and His438, which have an important role in the catalytic activity of BChE, with bond distance 1.91 Å, 3.29 Å, respectively (Figure 6).[Table t4-turkjchem-46-1-236]

In addition, compound **7f** was the most active compound against AChE and BChE in in vitro studies (IC_50_ = 40.07 ± 0.25, 52.39 ± 0.49), also exhibited better activity against AChE. Like this study, in silico molecular docking analysis, compound **7f** also showed better binding affinity with −8.19 kcal/mol binding energies against AChE (see in Table 3).

**Figure 7:**
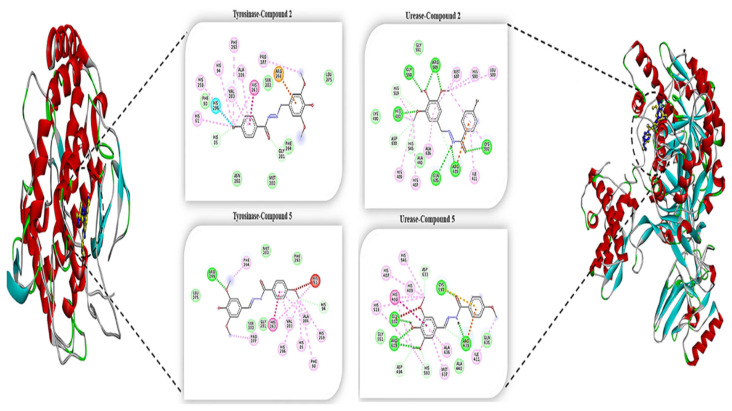
The 2D analysis of compounds **7b** and **7e** showing high efficiency against Tyrosinase and Urease.

Furthermore, compound **7b** exhibited the best binding efficacy against tyrosinase (Binding Energy: −6.20 kcal/mol) and urease (Binding Energy: −7.87 kcal/mol), and it also showed better activity than the positive control compounds (Kojic acid: Binding Energy: −3.96, Thiourea: Binding Energy: −3.32 kcal/mol) (see the Table). At the same time, as a result of in vitro analyses, compound **7e** was determined as the most effective compound for both tyrosinase and urease enzyme. This compound interacted with the pi-alkyl bond to conserved histidine residues of His61, His85, His259, His263, and His269, which were essential for the catalytic activity [53] of the tyrosinase enzyme. Additionally, this compound formed a very strong hydrogen bond interaction with the Cys592 in the mobile flap closing the active site of the urease enzyme [54] with bond distance of 2.12 Å (Figure 7).

In summary, based on this analysis, all synthesized compounds interacted with the catalytic functional sites of the target proteins. Also, intermolecular interactions that play an important role in the binding affinity of the ligand in the catalytic region of these target enzymes were elucidated at the molecular level.

### 3.5. Molecular properties, Lipinski rule, and ADME

**Figure 8:**
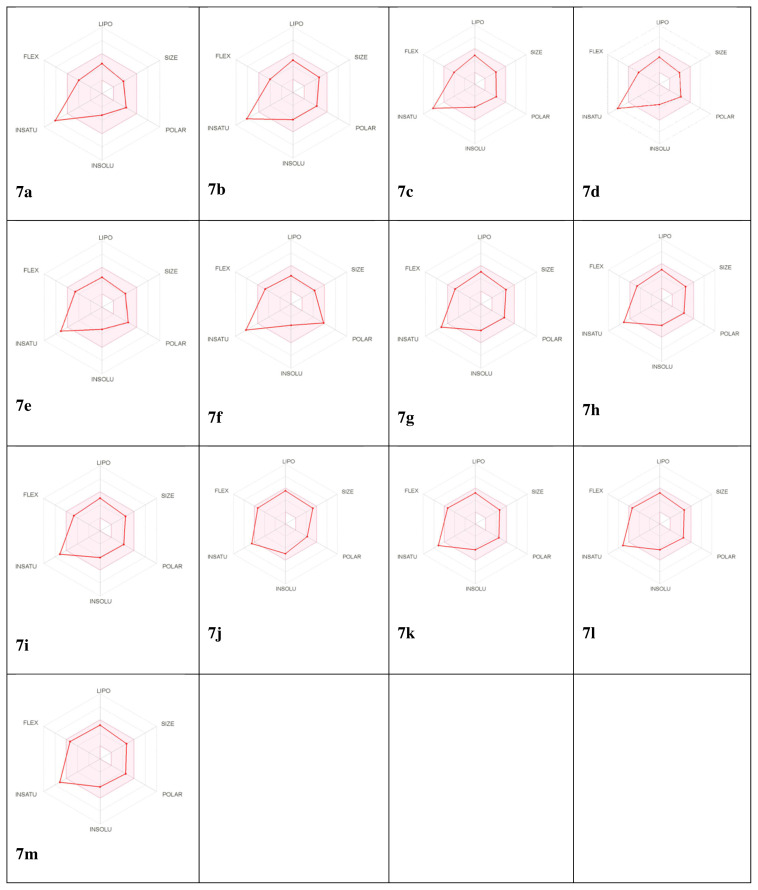
Bioavailability radar of the **7a**–**7m**. The pink area represents the optimal range for each properties (LIPO: Lipophilicity, SIZE: Molecular weight, POLAR: Total Polar Surface Area, INSOLU: Insolubility, INSATU: Instauration, FLEX: Flexibility). (For interpretation of the references to colour in this figure legend, the reader is referred to the web version of this article.)

The pink area indicates the optimal range for six physicochemical properties such as polarity, saturation, size, flexibility, lipophilicity, and solubility, and compound **7j** was the most ideal compound for this. The molecular weights and TPSA values of the **7a–7m** compounds, were in the range of A values of 300.31–436.31 and 80.15–125.97, respectively. Not all compounds cross the brain barrier. Therefore, it will not damage the central nervous system and thus will not cause depression and drowsiness (Figure 8) [55,56].

## 4. Conclusion

A series of benzoyl hydrazone were synthesized and evaluated their anticholinesterase inhibitory activity, tyrosinase inhibitory, and urease inhibitory activity. In the β-carotene-linoleic acid and ABTS cation radical scavenging activities, compounds **7j**, **7e**, and **7m** stand out as the most active compounds, respectively. According to the anticholinesterase enzyme inhibition activity results, compound **7f** exhibited the best activity against AChE and BChE enzymes in the synthesis series. İn silico studies will make significant contributions to the development of new active compounds for Alzheimer’s treatment and to the pharmaceutical industry in the future.

Fig. S1 FT-IR spectrum of compound **7a**

Fig. S2 ^1^H NMR spectrum of compound **7a**

Fig. S3 ^13^C NMR spectrum of compound **7a**

Fig. S4 FT-IR spectrum of compound **7b**

Fig. S5 ^1^H NMR spectrum of compound **7b**

Fig. S6 ^13^C NMR spectrum of compound **7b**

Fig. S7 LC-MS/MS spectrum of compound **7b**

Fig. S8 FT-IR spectrum of compound **7c**

Fig. S9 ^1^H NMR spectrum of compound **7c**

Fig. S10 ^13^C NMR spectrum of compound **7c**

Fig. S11 LC-MS/MS spectrum of compound **7c**

Fig. S12 FT-IR spectrum of compound **7d**

Fig. S13 ^1^H NMR spectrum of compound **7d**

Fig. S14 ^13^C NMR spectrum of compound **7d**

Fig. S15 LC-MS/MS spectrum of compound **7d**

Fig. S16 FT-IR spectrum of compound **7e**

Fig. S17 ^1^H NMR spectrum of compound **7e**

Fig. S18 ^13^C NMR spectrum of compound **7e**

Fig. S19 LC-MS/MS spectrum of compound **7e**

Fig. S20 FT-IR spectrum of compound **7f**

Fig. S21 ^1^H NMR spectrum of compound **7f**

Fig. S22 ^13^C NMR spectrum of compound **7f**

Fig. S23 FT-IR spectrum of compound **7g**

Fig. S24 ^1^H NMR spectrum of compound **7g**

Fig. S25 ^13^C NMR spectrum of compound **7g**

Fig. S26 LC-MS/MS spectrum of compound **7g**

Fig. S27 FT-IR spectrum of compound **7h**

Fig. S28 ^1^H NMR spectrum of compound **7h**

Fig. S29 ^13^C NMR spectrum of compound **7h**

Fig. S30 LC-MS/MS spectrum of compound **7h**

Fig. S31 FT-IR spectrum of compound 7i

Fig. S32 ^1^H NMR spectrum of compound 7i

Fig. S33 ^13^C NMR spectrum of compound 7i

Fig. S34 FT-IR spectrum of compound **7j**

Fig. S35 ^1^H NMR spectrum of compound **7j**

Fig. S36 ^13^C NMR spectrum of compound **7j**

Fig. S37 LC-MS/MS spectrum of compound **7j**

Fig. S38 FT-IR spectrum of compound **7k**

Fig. S39 ^1^H NMR spectrum of compound **7k**

Fig. S40 ^13^C NMR spectrum of compound **7k**

Fig. S41 LC-MS/MS spectrum of compound **7k**

Fig. S42 FT-IR spectrum of compound **7l**

Fig. S43 ^1^H NMR spectrum of compound **7l**

Fig. S44 ^13^C NMR spectrum of compound **7l**

Fig. S45 LC-MS/MS spectrum of compound **7l**

Fig. S46 FT-IR spectrum of compound **7m**

Fig. S47 ^1^H NMR spectrum of compound **7m**

Fig. S48 ^13^C NMR spectrum of compound **7m**

Fig. S49 LC-MS/MS spectrum of compound **7m**

## Figures and Tables

**Table 1:** NMR data in DMSO, chemical shifts (δ, ppm) for compound **7**l.

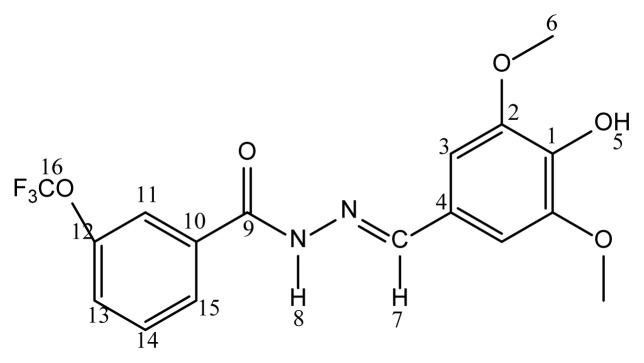				
Number	^13^C NMR	^1^H NMR	HETCOR NMR	HMBC NMR
1	139.12	--------		C_1_-H_3,6_
2	148.69	--------		
3	105.24	6.99 (s, 2H)	H_3_-C_3_	
4	121.82	--------		
5	--------	3.74 (s, 1H)		
6	56.48	3.82 (s, 6H)	H_6_-C_6_	C_6_-H_5_
7	149.77	8.35 (s, 1H)	H_7_-C_7_	C_7_-H_3_
8	--------	6.84 (s, 1H)		
9	161.81	--------		
10	136.41	--------		C_10_-H_11_,_15_
11	120.58	7.87 (s, 1H)	H_11_-C_11_	
12	148.77	-------		
13	124.53	7.61 (d, 1H)	H_13_-C_13_	
14	131.17	7.68 (t, 1H)	H_14_-C_14_	
15	127.22	7.97 (d, 1H)	H_15_-C_15_	
16	119.26	-------		C_16_-H_11_

**Table 2:** Antioxidant activity results of synthesized compounds **7a–7m**^a^.

Compound	Antioxidant Activity			
	*β*-carotene-linoleic acid (IC_50_ μM/mL)	DPPH. activity (IC_50_ μM/mL)	ABTS^.+^ activity (IC_50_ μM/mL)	CUPRAC capacity (A_0.5_ μM)
**7a**	43.24 ± 0.08	35.14 ± 0.53	41.86 ± 0.11	39.29 ± 0.01
**7b**	59.76 ± 0.40	53.11 ± 0.67	60.35 ± 0.60	65.09 ± 0.00
**7c**	39.04 ± 0.56	31.46 ± 0.73	38.22 ± 0.61	31.52 ± 0.00
**7d**	32.82 ± 0.09	26.75 ± 0.18	33.14 ± 0.32	28.85 ± 0.00
**7e**	16.55 ± 0.33	6.02 ± 0.72	8.97 ± 0.20	10.08 ± 0.01
**7f**	47.06 ± 0.82	38.74 ± 0.44	43.19 ± 0.74	46.70 ± 0.02
**7g**	27.61 ± 0.18	22.08 ± 0.92	26.38 ± 0.50	23.64 ± 0.02
**7h**	54.58 ± 0.75	50.72 ± 0.71	56.43 ± 0.16	61.02 ± 0.01
**7i**	20.94 ± 0.25	13.10 ± 0.66	17.41 ± 0.64	17.92 ± 0.02
**7j**	14.59 ± 0.81	5.87 ± 0.48	7.65 ± 0.27	9.88 ± 0.01
**7k**	24.51 ± 0.43	16.27 ± 0.35	21.34 ± 0.19	20.35 ± 0.02
**7l**	51.39 ± 0.24	42.15 ± 0.04	49.57 ± 0.39	54.29 ± 0.01
**7m**	19.68 ± 0.73	8.09 ± 0.11	14.17 ± 0.82	15.66 ± 0.00
**α** **-TOC** b	4.50 ± 0.09	12.26 ± 0.07	4.87 ± 0.45	40.55 ± 0.04
**BHT** b	2.34 ± 0.09	54.97 ± 0.99	2.91 ± 0.55	4.00 ± 0.04

a Values expressed are means ± S.E.M. of three parallel measurements. *P* < 0.05, significantly different with student’s *t*-test.

b Reference compound.

**Table 3:** Results of enzyme inhibition activities of synthesized compounds **7a–7m**^a^.

Compound	Anticholinesterase Activity		*Tyrosinase activity* (IC_50_ mM/mL)	*Urease activity*
	*AChE assay* (IC_50_ μM/mL) (IC_50_ μM/mL)	*BChE assay* (IC_50_ μM/mL)		
**7a**	101.04 ± 0.49	122.04 ± 0.87	88.73 ± 0.26	144.50 ± 0.67
**7b**	93.00 ± 1.11	116.82 ± 0.66	82.08 ± 1.09	139.00 ± 0.08
**7c**	82.14 ± 0.56	102.87 ± 0.48	70.91 ± 0.24	121.31 ± 0.26
**7d**	88.37 ± 0.11	109.44 ± 0.09	64.38 ± 0.99	113.62 ± 0.59
**7e**	75.01 ± 0.94	96.33 ± 0.61	10.15 ± 0.04	54.18 ± 0.19
**7f**	40.07 ± 0.25	52.39 ± 0.49	18.22 ± 0.35	61.02 ± 0.99
**7g**	70.13 ± 0.89	83.08 ± 0.34	61.07 ± 0.55	108.67 ± 0.16
**7h**	73.75 ± 0.34	88.70 ± 0.77	56.88 ± 0.37	91.31 ± 0.87
**7i**	51.08 ± 0.41	64.09 ± 0.30	49.73 ± 0.12	89.03 ± 0.75
**7j**	44.83 ± 0.12	57.30 ± 0.87	40.08 ± 0.86	82.68 ± 0.32
**7k**	59.31 ± 0.77	69.18 ± 0.98	29.78 ± 0.43	69.16 ± 0.28
**7l**	68.09 ± 0.02	80.62 ± 0.36	24.30 ± 0.78	63.87 ± 0.44
**7m**	62.04 ± 0.39	77.26 ± 0.42	36.62 ± 0.56	78.34 ± 0.05
**Galantamine** b	4.53 ± 0.61	48.14 ± 0.14	NT	NT
**Kojic acid** b	NT	NT	0.71 ± 0.33	NT
**L-mimosine** b	NT	NT	0.79 ± 0.47	NT
**Thiourea** b	NT	NT	NT	24.20 ± 0.3

a Values expressed are means ± S.E.M. of three parallel measurements. *P* < 0.05, significantly different with student’s *t*-test.

b Reference compound.

NT: Not tested.

**Table 4 t4-turkjchem-46-1-236:** Physicochemical parameters, Druglikeness, Lipophilicity, and Pharmacokinetics of the synthesized compounds.

Parameter	Physicochemical parameters, Druglikeness, Lipophilicity, Pharmacokinetics												
	7a	7b	7c	7d	7e	7f	7g	7h	7i	7j	7k	7l	7m
**Formula**	C_16_H_16_N_2_O_4_	C_16_H_15_BrN_2_O_4_	C_16_H_15_ClN_2_O_4_	C_16_H_15_FN_2_O_4_	C_17_H_18_N_2_O_5_	C_16_H_15_N_3_O_6_	C_17_H_15_F_3_N_2_O_4_	C_17_H_15_F_3_N_2_O_4_	C_17_H_15_F_3_N_2_O_4_	C_17_H_15_F_3_N_2_O_4_	C_17_H_15_FN_2_O_5_	C_17_H_15_F_3_N_2_O_5_	C_17_H_15_F_3_N_2_O_5_
**Molecular weight (g/mol)**	300.31	379,21	334.75	318.30	330.34	345.31	368.31	368.31	368.31	436.31	384.31	384.31	384.31
**Num. heavy atoms**	22	23	23	23	24	25	26	26	26	30	27	27	27
**Num. arom. heavy atoms**	12	12	12	12	12	12	12	12	12	12	12	12	12
**Fraction Csp** **^3^**	0.12	0.12	0.12	0.12	0.18	0.12	0.18	0.18	0.18	0.22	0.18	0.18	0.18
**Num. H-bond acceptors**	5	5	5	6	6	7	8	8	8	11	9	9	9
**Num. H-bond donors**	2	2	2	2	2	2	2	2	2	2	2	2	2
**Molar Refractivity**	82.62	90.32	87.63	82.58	89.11	91.44	87.62	87.62	87.62	92.62	89.30	89.30	89.30
**TPSA**	80.15 Å^2^	80.15 Å^2^	80.15 Å^2^	80.15 Å^2^	89.38 Å^2^	125.97 Å^2^	80.15 Å^2^	80.15 Å^2^	80.15 Å^2^	80.15 Å^2^	89.38 Å^2^	89.38 Å^2^	89.38 Å^2^
**Lipinski**	Yes	Yes	Yes	Yes	Yes	Yes	Yes	Yes	Yes	Yes	Yes	Yes	Yes
**Ghose**	Yes	Yes	Yes	Yes	Yes	Yes	Yes	Yes	Yes	Yes	Yes	Yes	Yes
**Veber**	Yes	Yes	Yes	Yes	Yes	Yes	Yes	Yes	Yes	Yes	Yes	Yes	Yes
**Egan**	Yes	Yes	Yes	Yes	Yes	Yes	Yes	Yes	Yes	Yes	Yes	Yes	Yes
**Bioavailability Score**	0.55	0.55	0.55	0.55	0.55	0.55	0.55	0.55	0.55	0.55	0.55	0.55	0.55
**Log Po/w (iLOGP)**	2.38	2.82	2.74	2.62	2.51	2.09	2.60	2.64	2.68	2.89	2.83	2.96	2.83
**GI absorption**	High	High	High	High	High	High	High	High	High	Low	High	High	High
**BBB permeant**	No	No	No	No	No	No	No	No	No	No	No	No	No
