# Supplementary figures and images for: Synthesis of benzoyl hydrazones having 4-hydroxy-3,5-dimethoxy phenyl ring, their biological activities, and molecular modeling studies on enzyme inhibition activities
Source: Turk J Chem. 2021 Oct 22;46(1):236–52. doi: 10.3906/kim-2107-7 (PMC10734773; doi:10.3906/kim-2107-7)

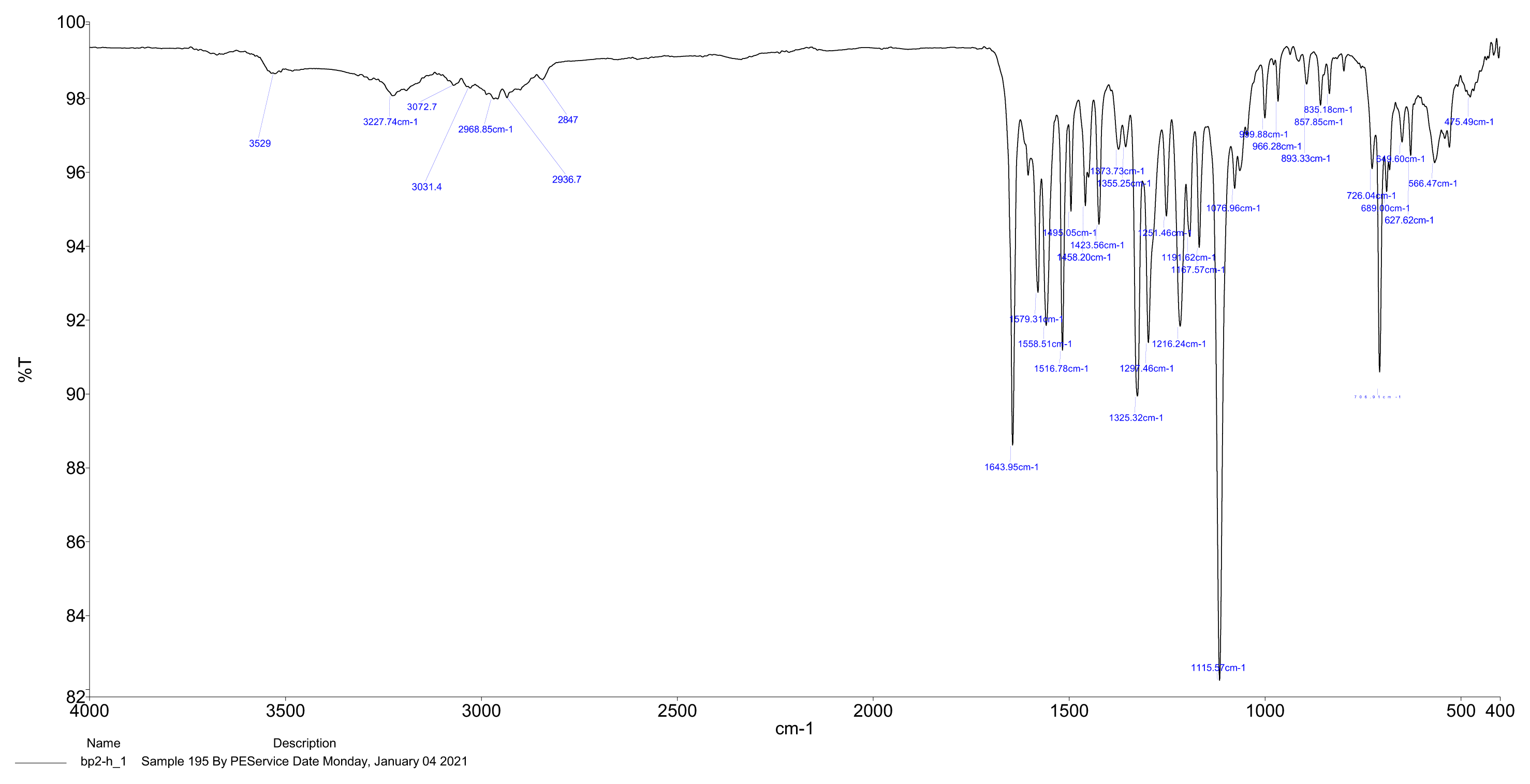

Supplement: Fig. S1 — FT-IR spectrum of compound 7a [file turkjchem-46-1-236s1.tif]

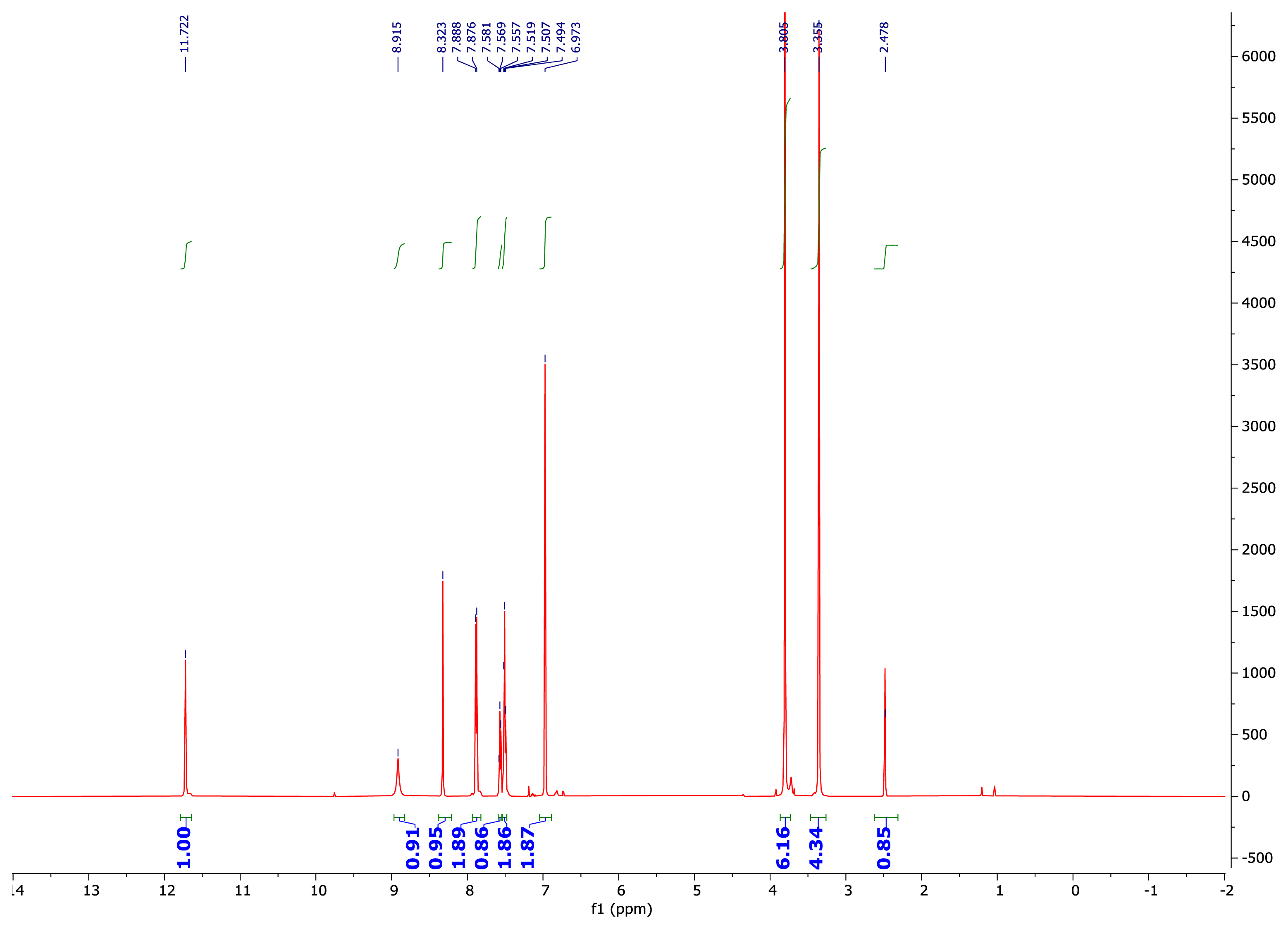

Supplement: Fig. S2 — 1H NMR spectrum of compound 7a [file turkjchem-46-1-236s2.tif]

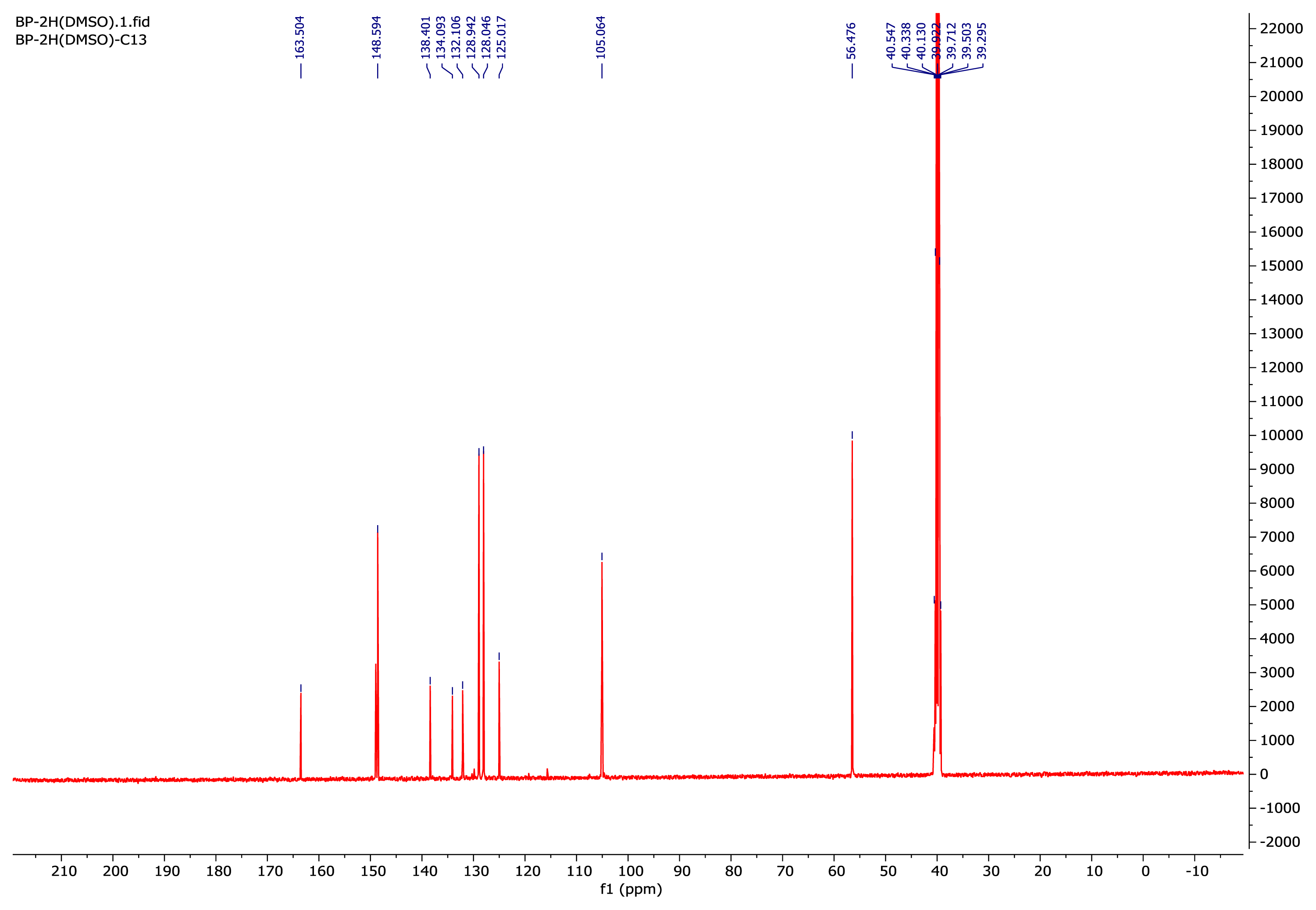

Supplement: Fig. S3 — 13C NMR spectrum of compound 7a [file turkjchem-46-1-236s3.tif]

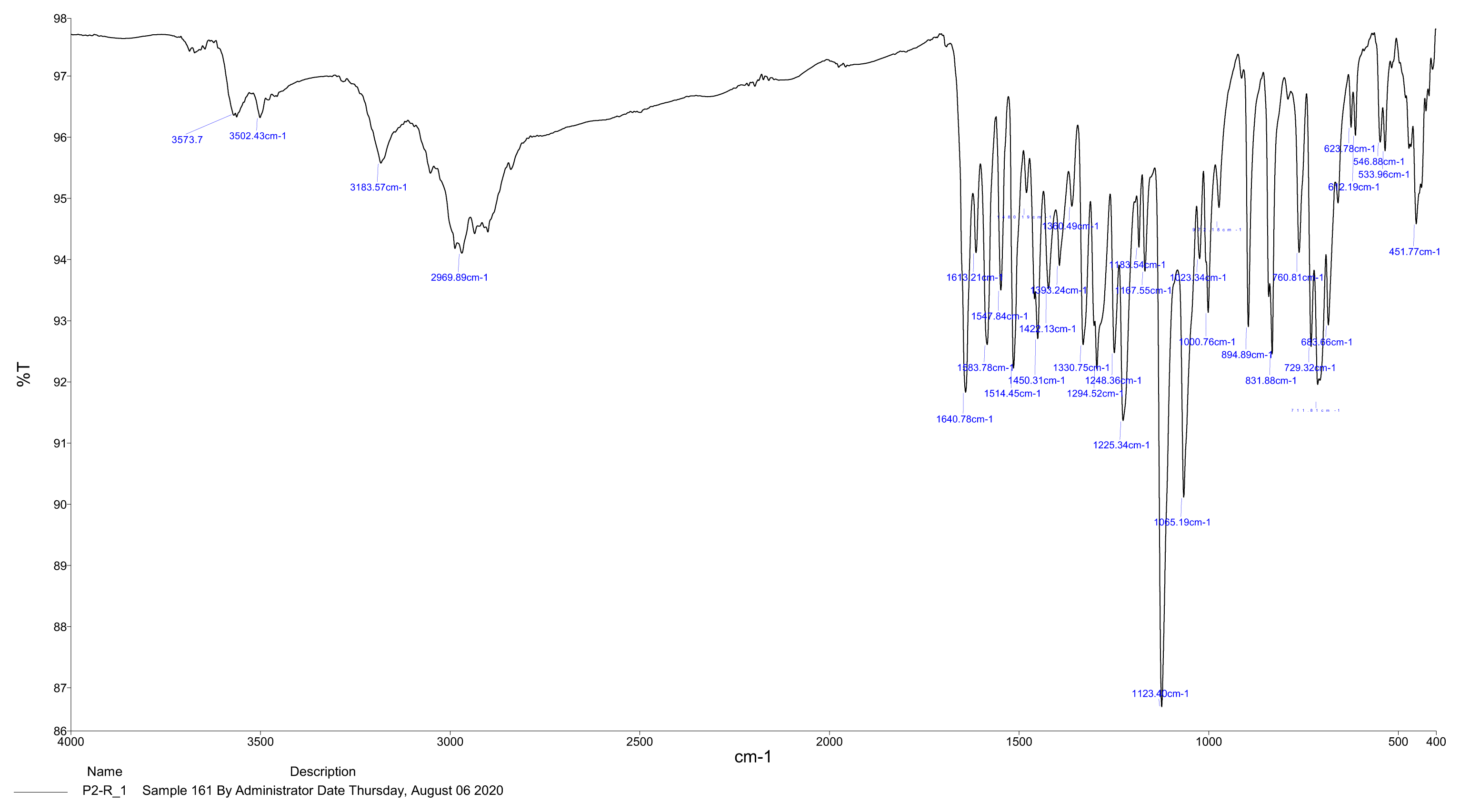

Supplement: Fig. S4 — FT-IR spectrum of compound 7b [file turkjchem-46-1-236s4.tif]

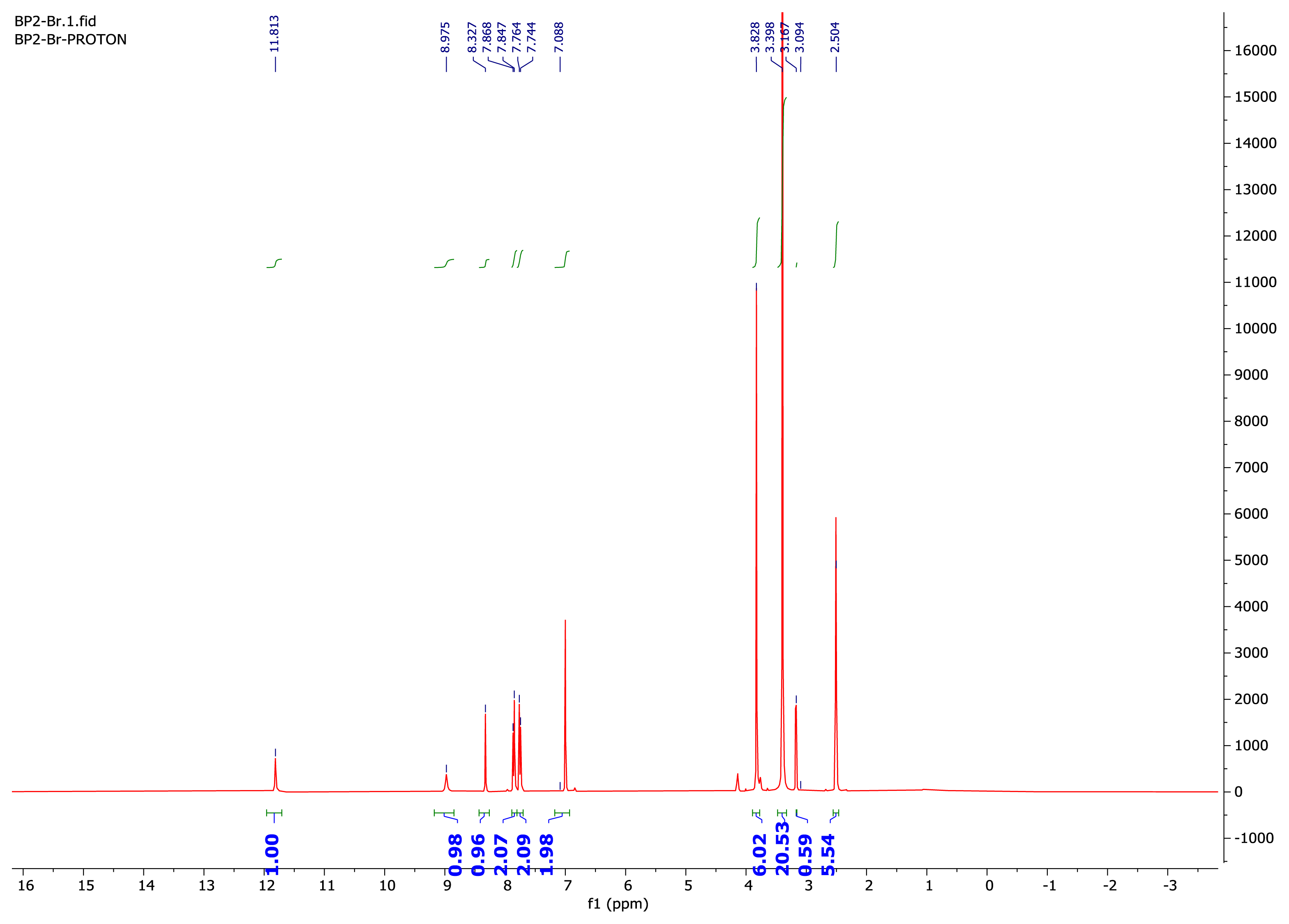

Supplement: Fig. S5 — 1H NMR spectrum of compound 7b [file turkjchem-46-1-236s5.tif]

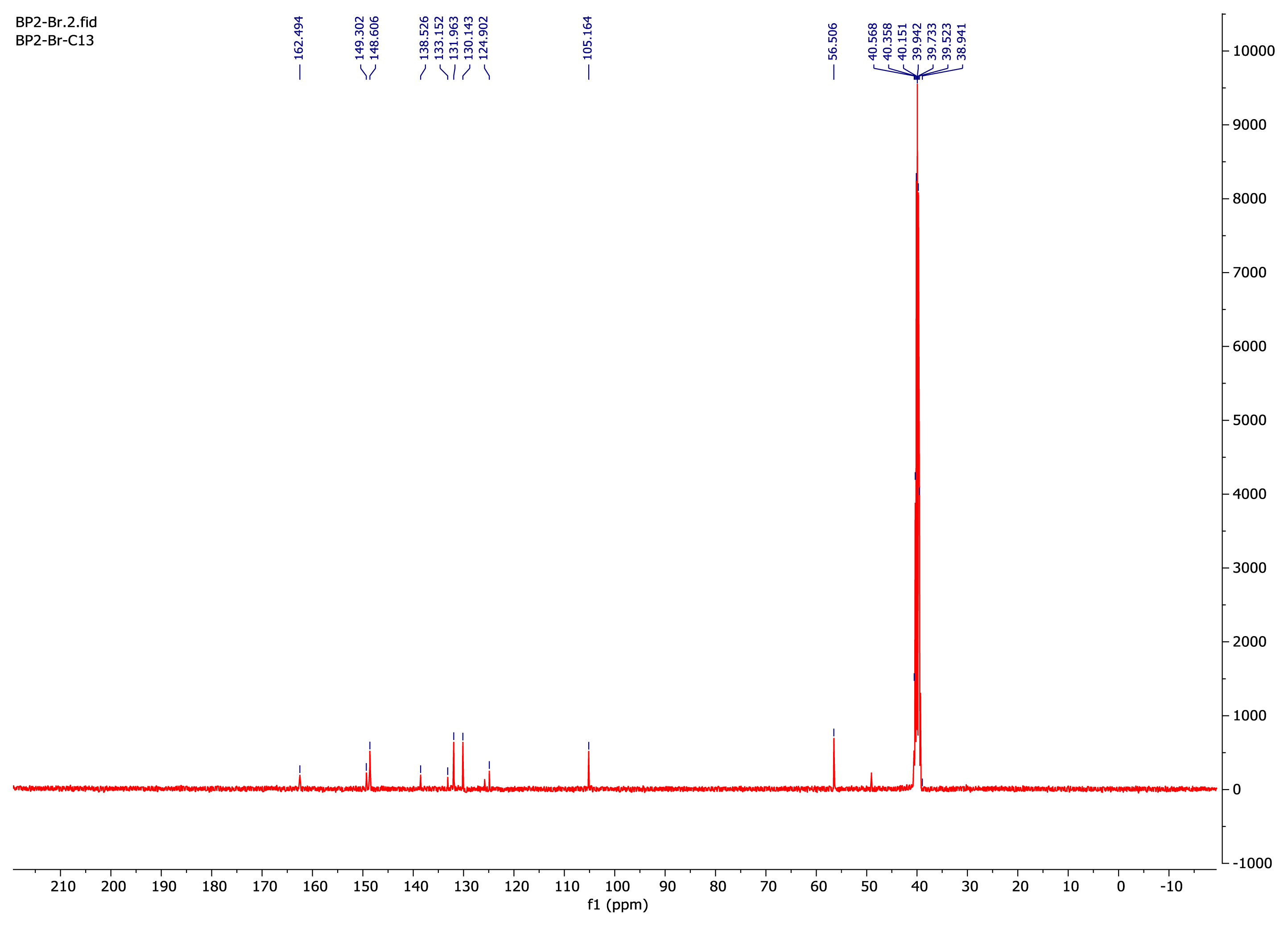

Supplement: Fig. S6 — 13C NMR spectrum of compound 7b [file turkjchem-46-1-236s6.tif]

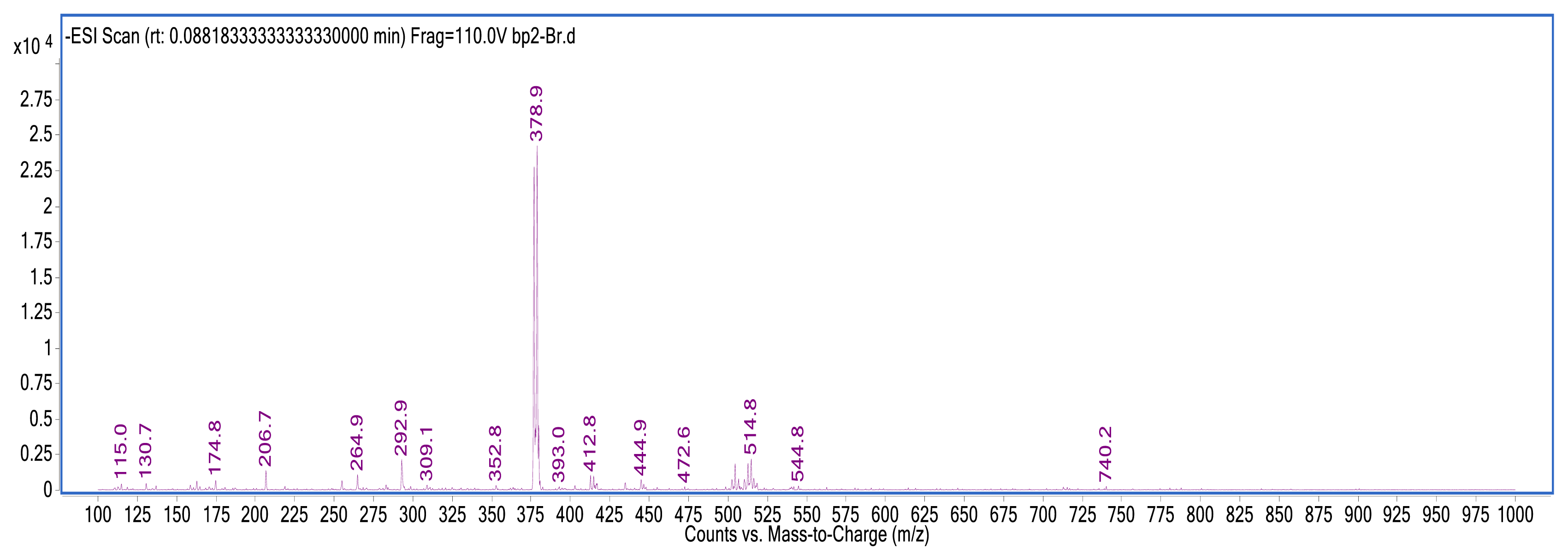

Supplement: Fig. S7 — LC-MS/MS spectrum of compound 7b [file turkjchem-46-1-236s7.tif]

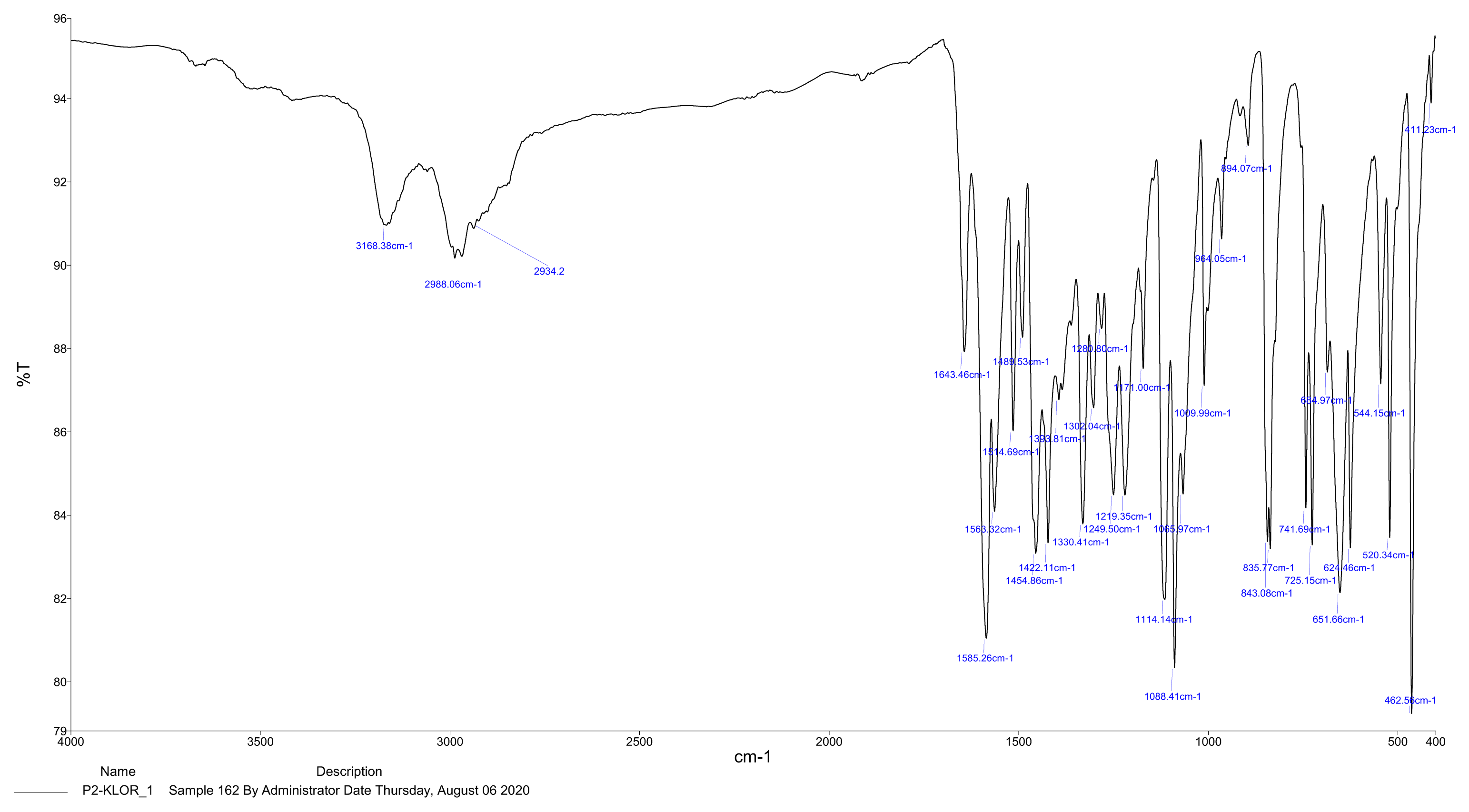

Supplement: Fig. S8 — FT-IR spectrum of compound 7c [file turkjchem-46-1-236s8.tif]

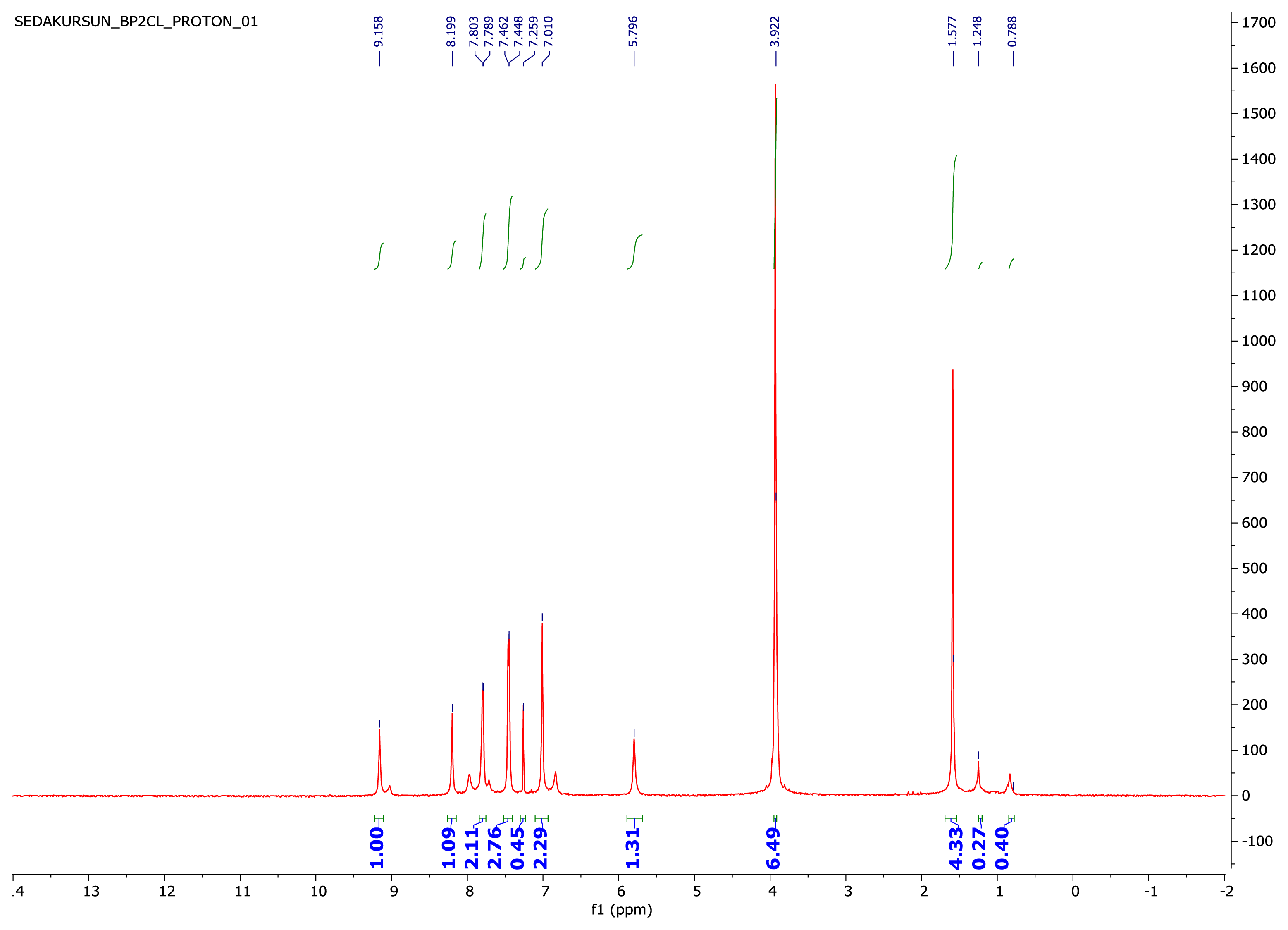

Supplement: Fig. S9 — 1H NMR spectrum of compound 7c [file turkjchem-46-1-236s9.tif]

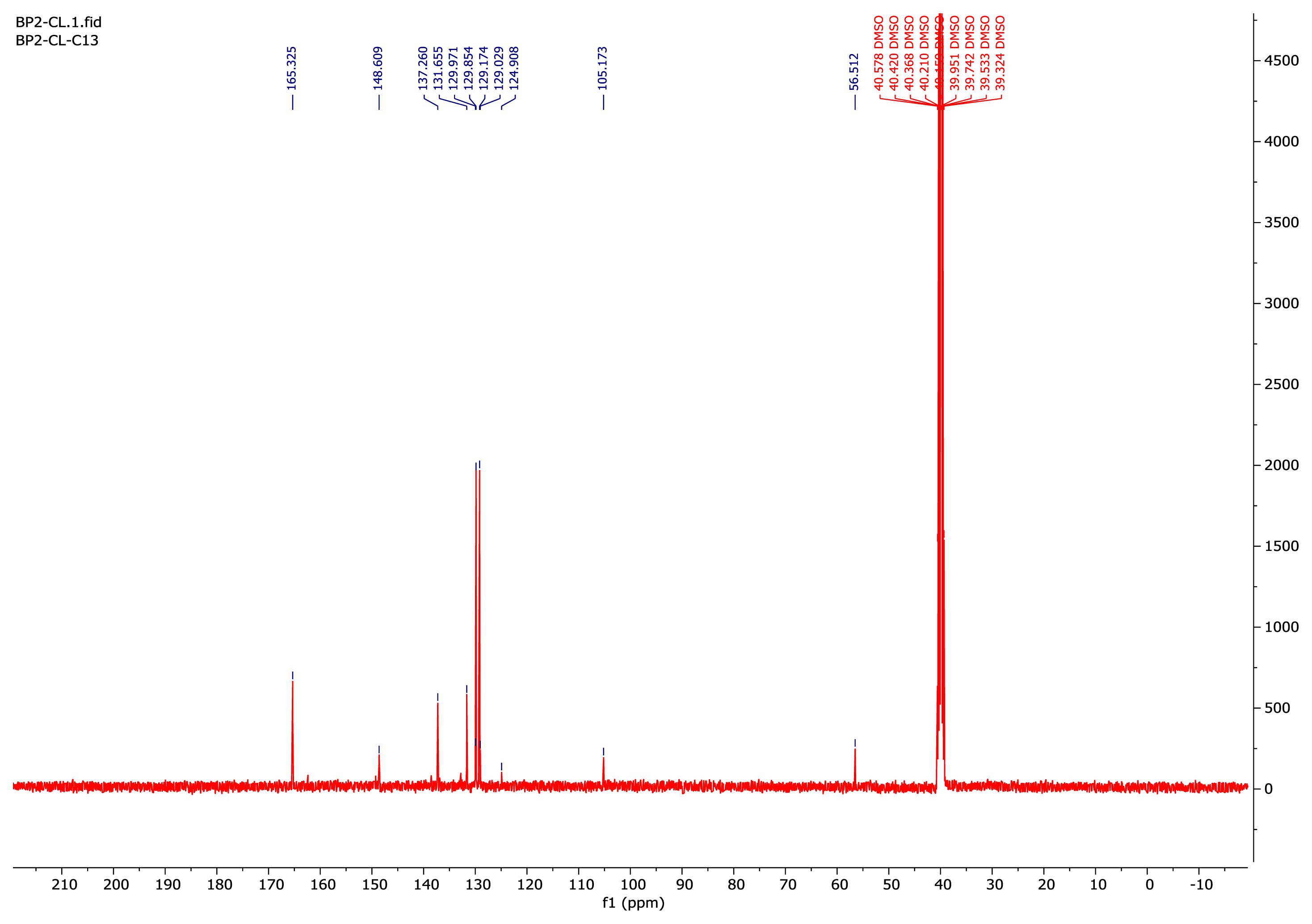

Supplement: Fig. S10 — 13C NMR spectrum of compound 7c [file turkjchem-46-1-236s10.tif]

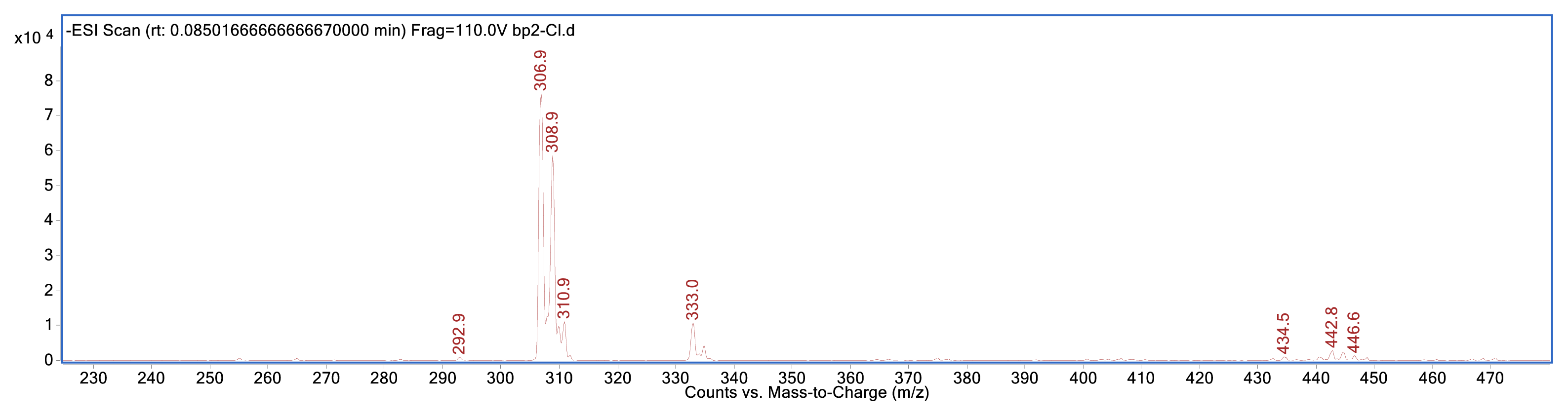

Supplement: Fig. S11 — LC-MS/MS spectrum of compound 7c [file turkjchem-46-1-236s11.tif]

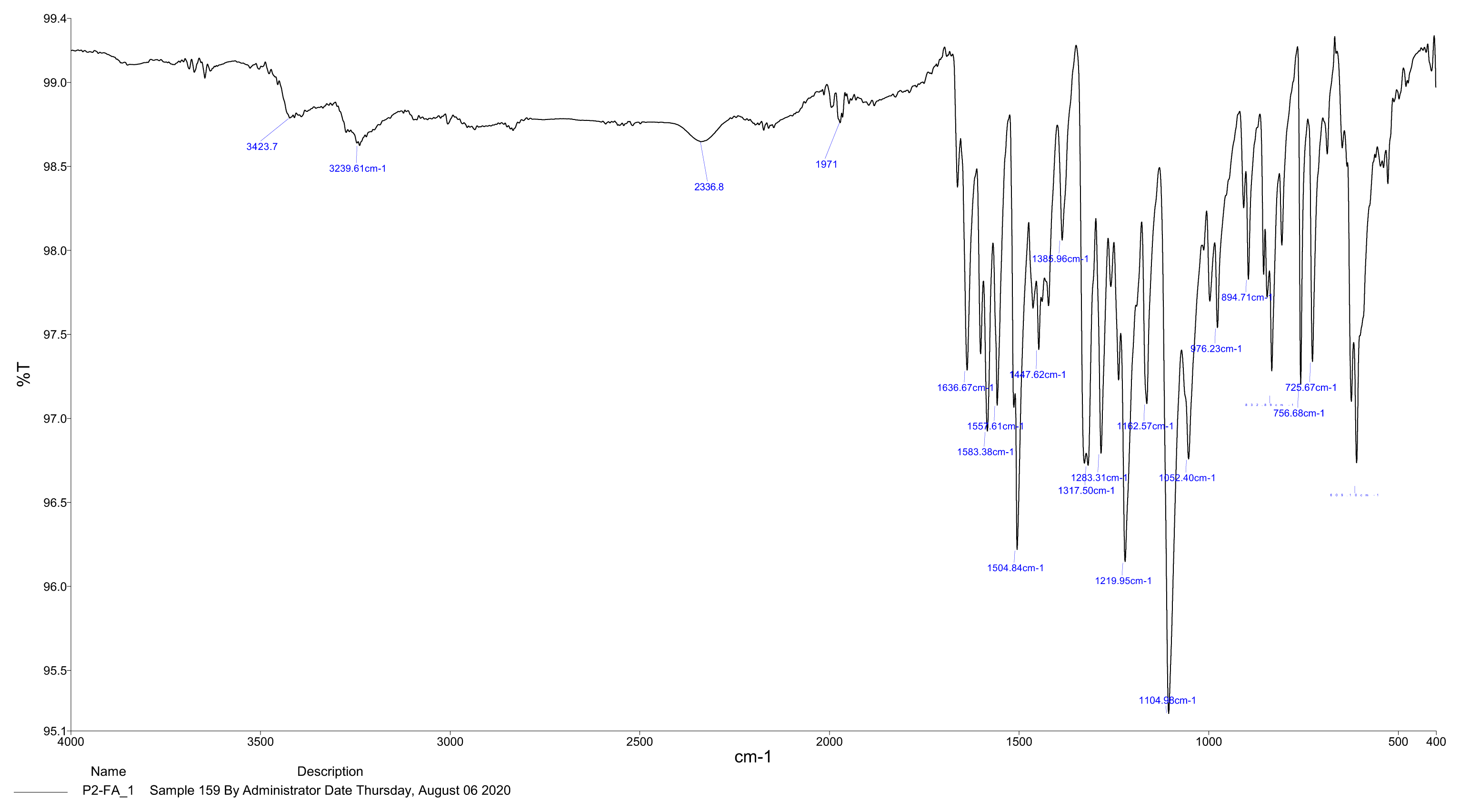

Supplement: Fig. S12 — FT-IR spectrum of compound 7d [file turkjchem-46-1-236s12.tif]

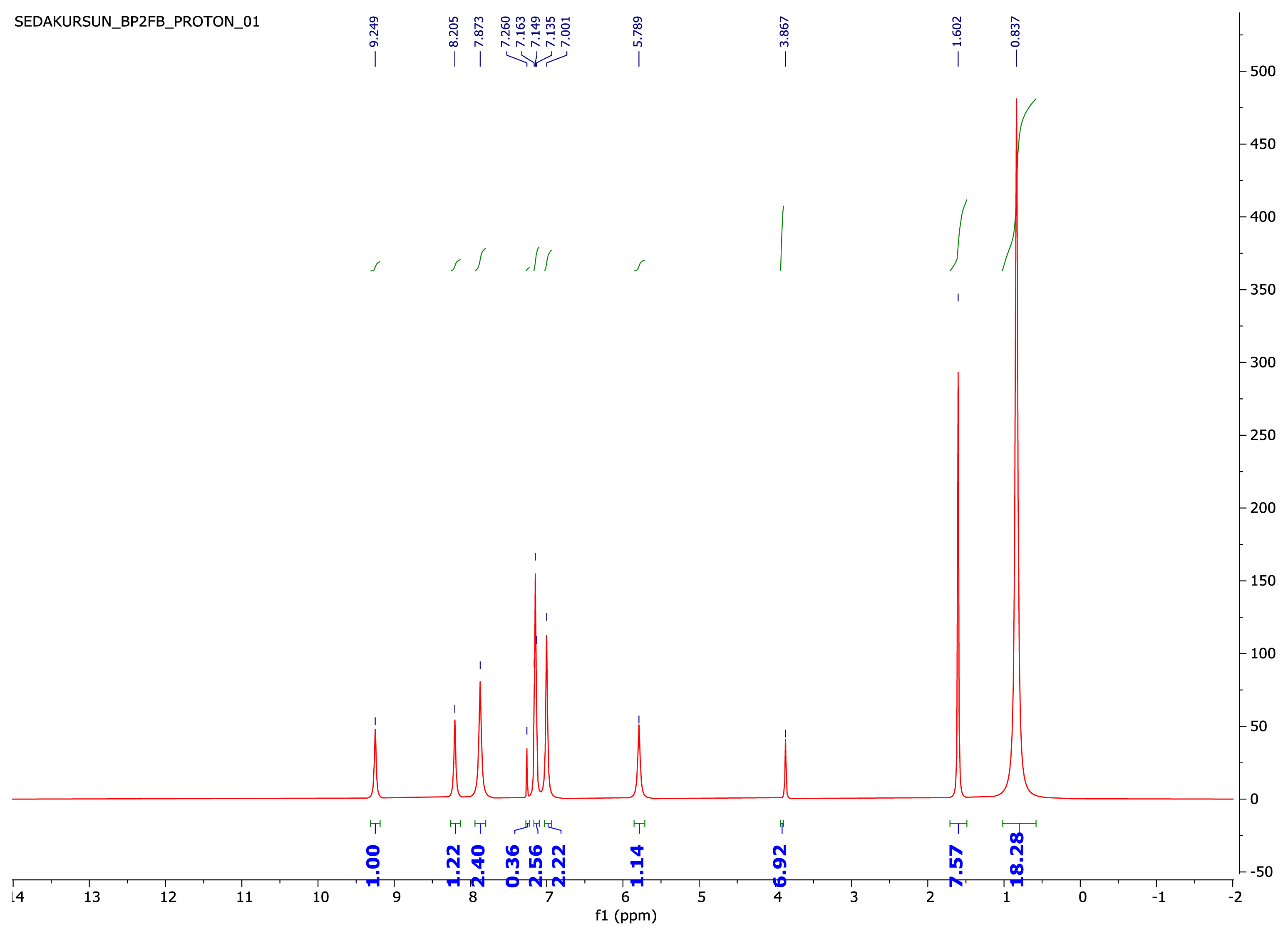

Supplement: Fig. S13 — 1H NMR spectrum of compound 7d [file turkjchem-46-1-236s13.tif]

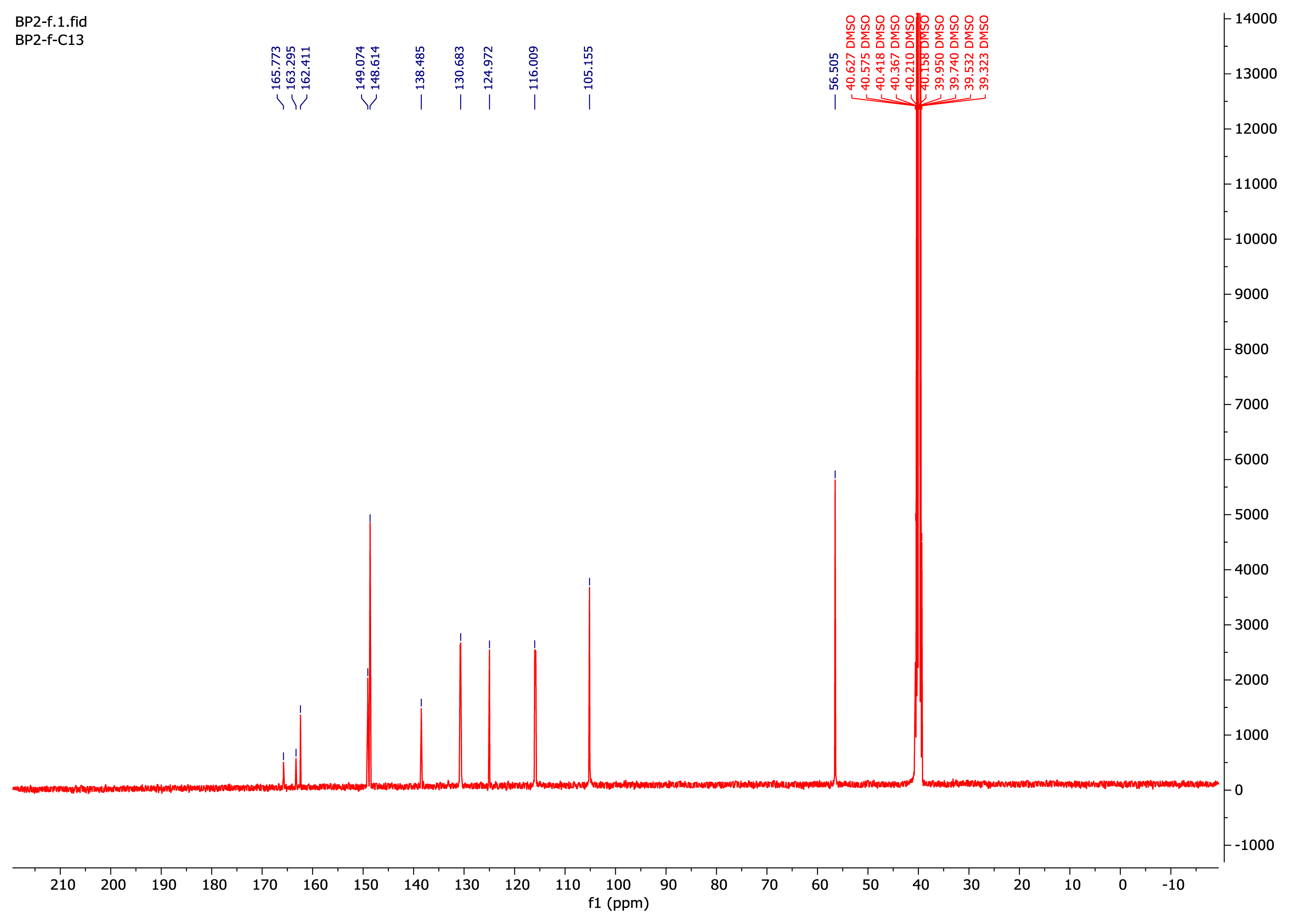

Supplement: Fig. S14 — 13C NMR spectrum of compound 7d [file turkjchem-46-1-236s14.tif]

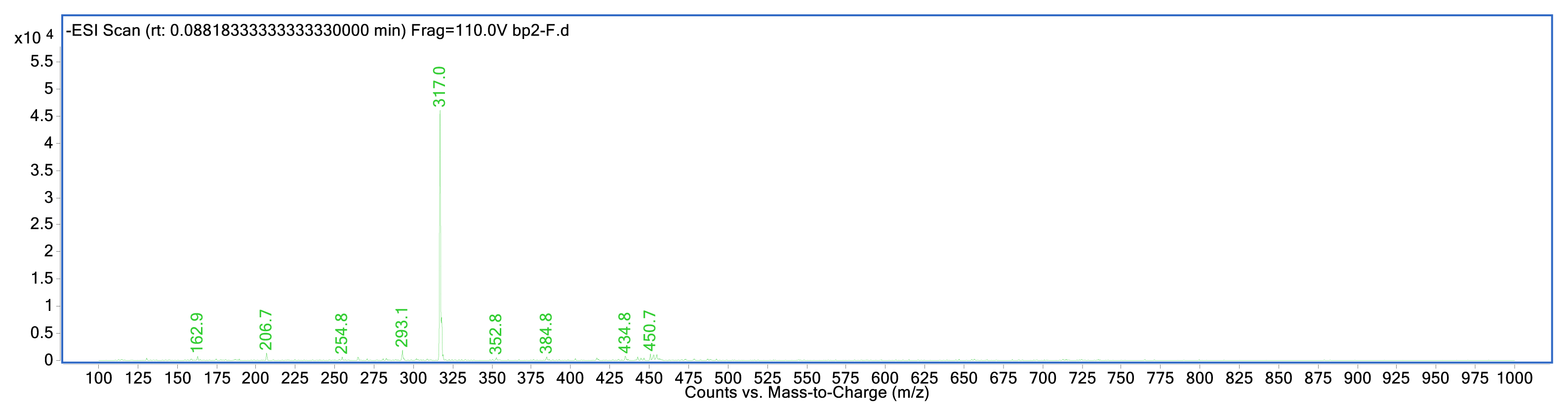

Supplement: Fig. S15 — LC-MS/MS spectrum of compound 7d [file turkjchem-46-1-236s15.tif]

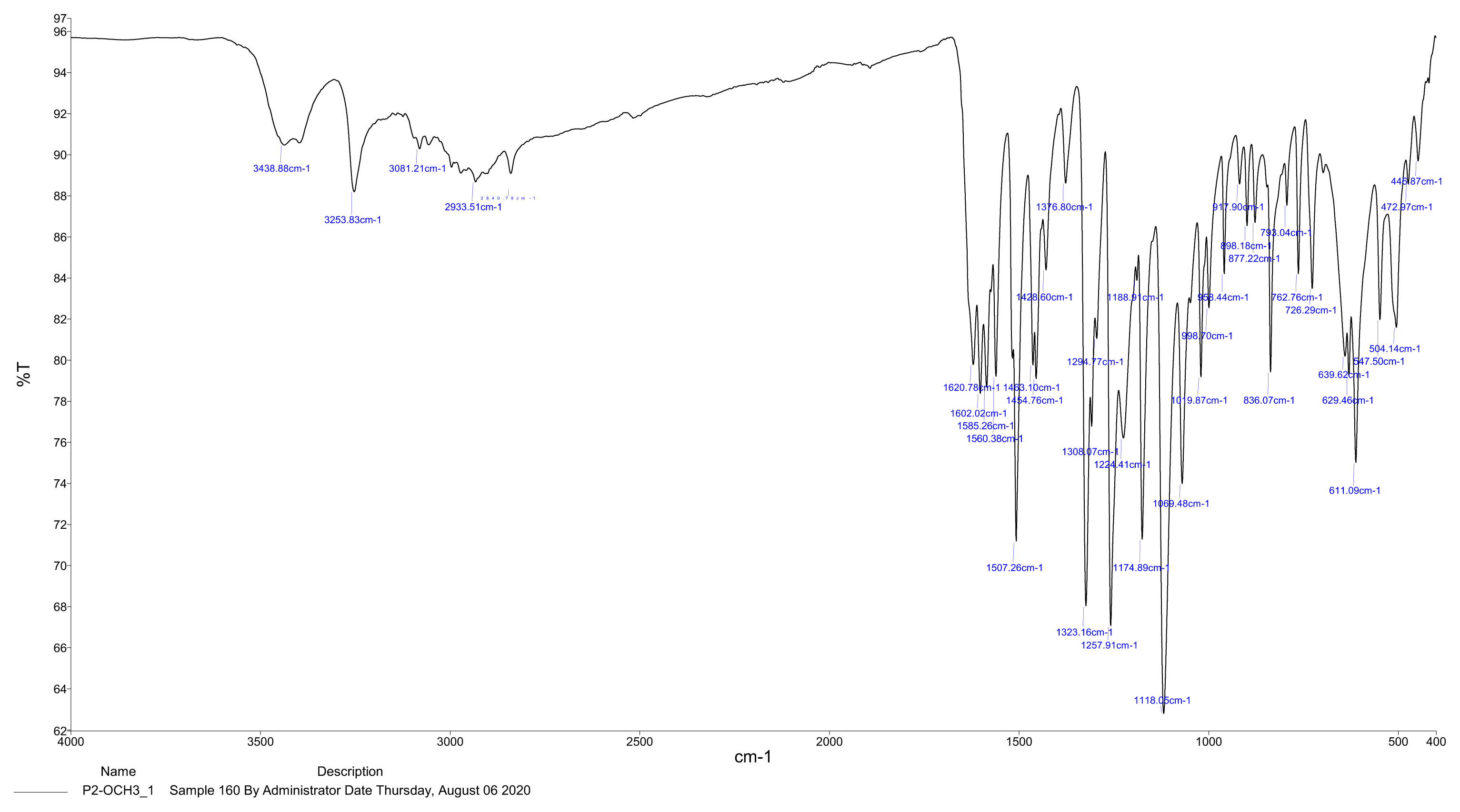

Supplement: Fig. S16 — FT-IR spectrum of compound 7e [file turkjchem-46-1-236s16.tif]

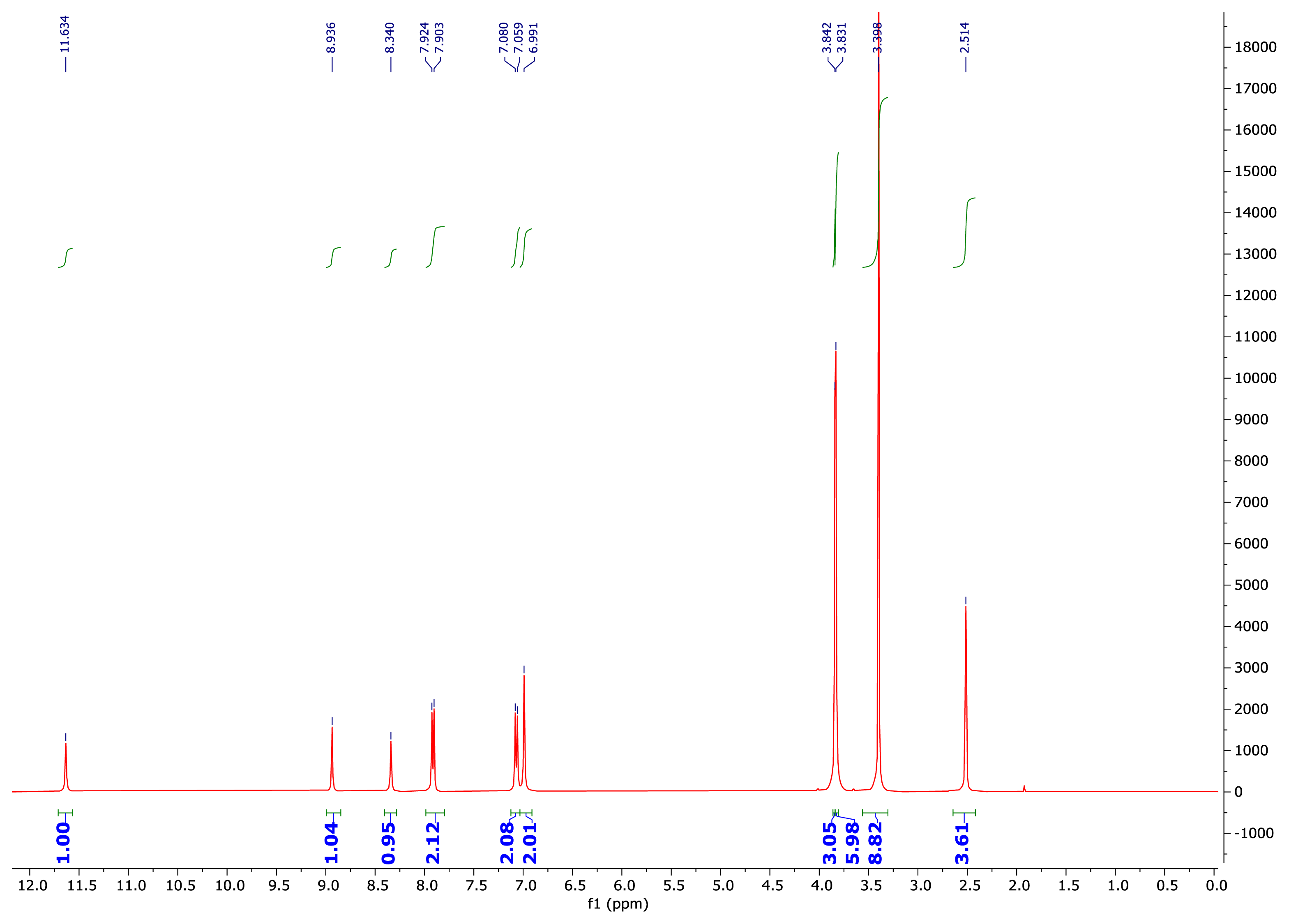

Supplement: Fig. S17 — 1H NMR spectrum of compound 7e [file turkjchem-46-1-236s17.tif]

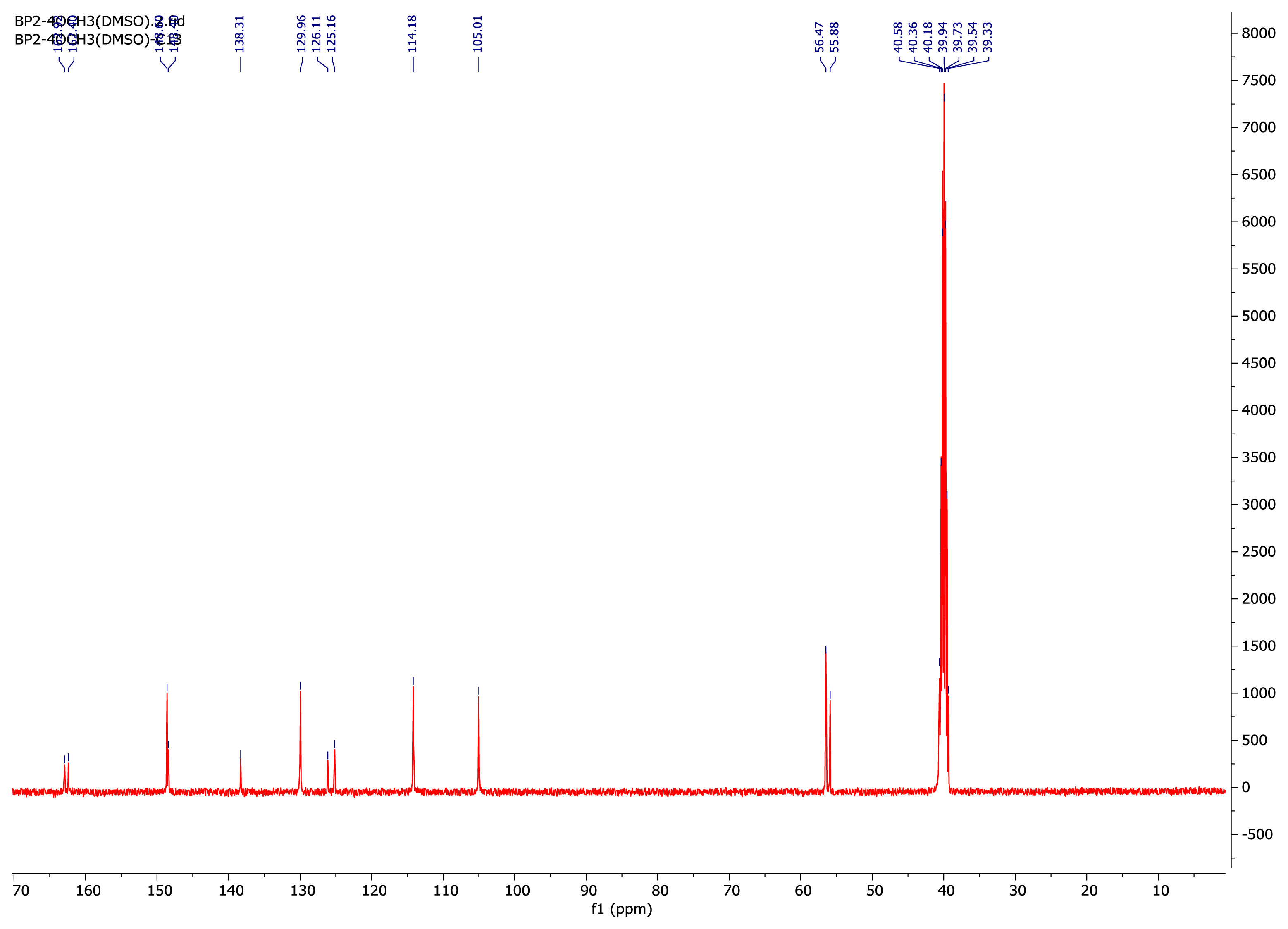

Supplement: Fig. S18 — 13C NMR spectrum of compound 7e [file turkjchem-46-1-236s18.tif]

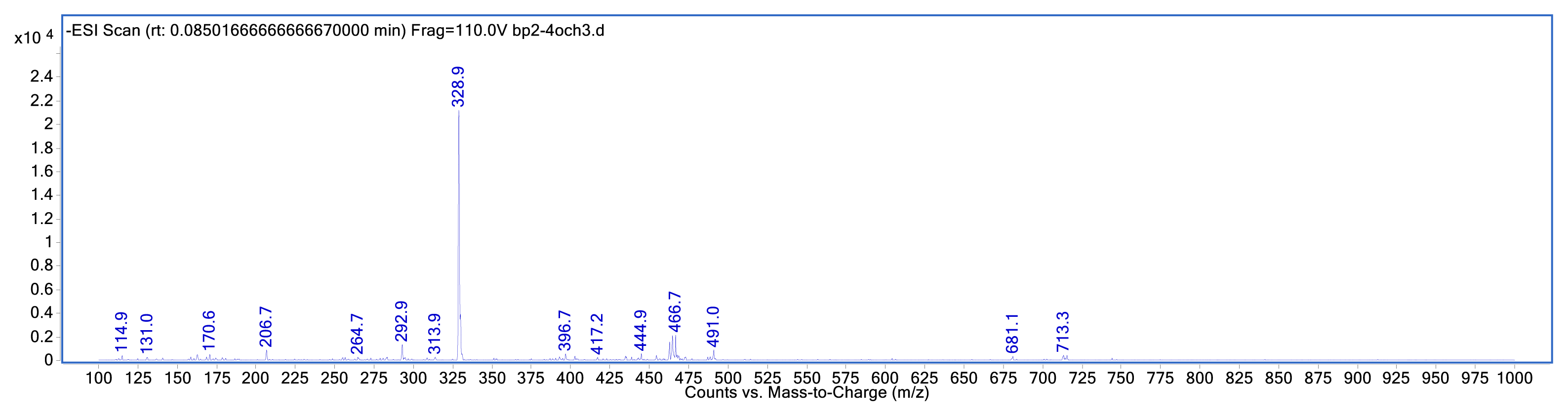

Supplement: Fig. S19 — LC-MS/MS spectrum of compound 7e [file turkjchem-46-1-236s19.tif]

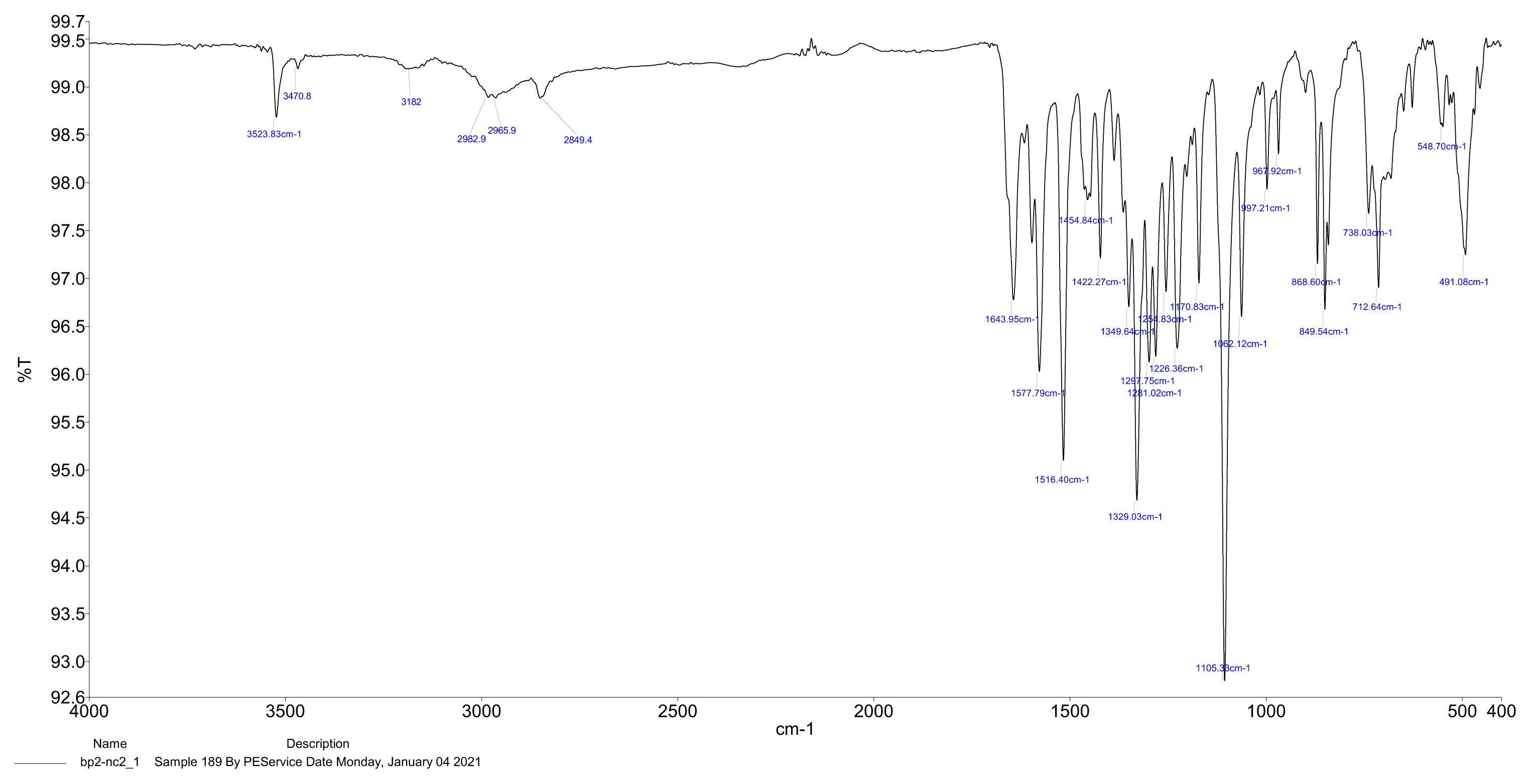

Supplement: Fig. S20 — FT-IR spectrum of compound 7f [file turkjchem-46-1-236s20.tif]

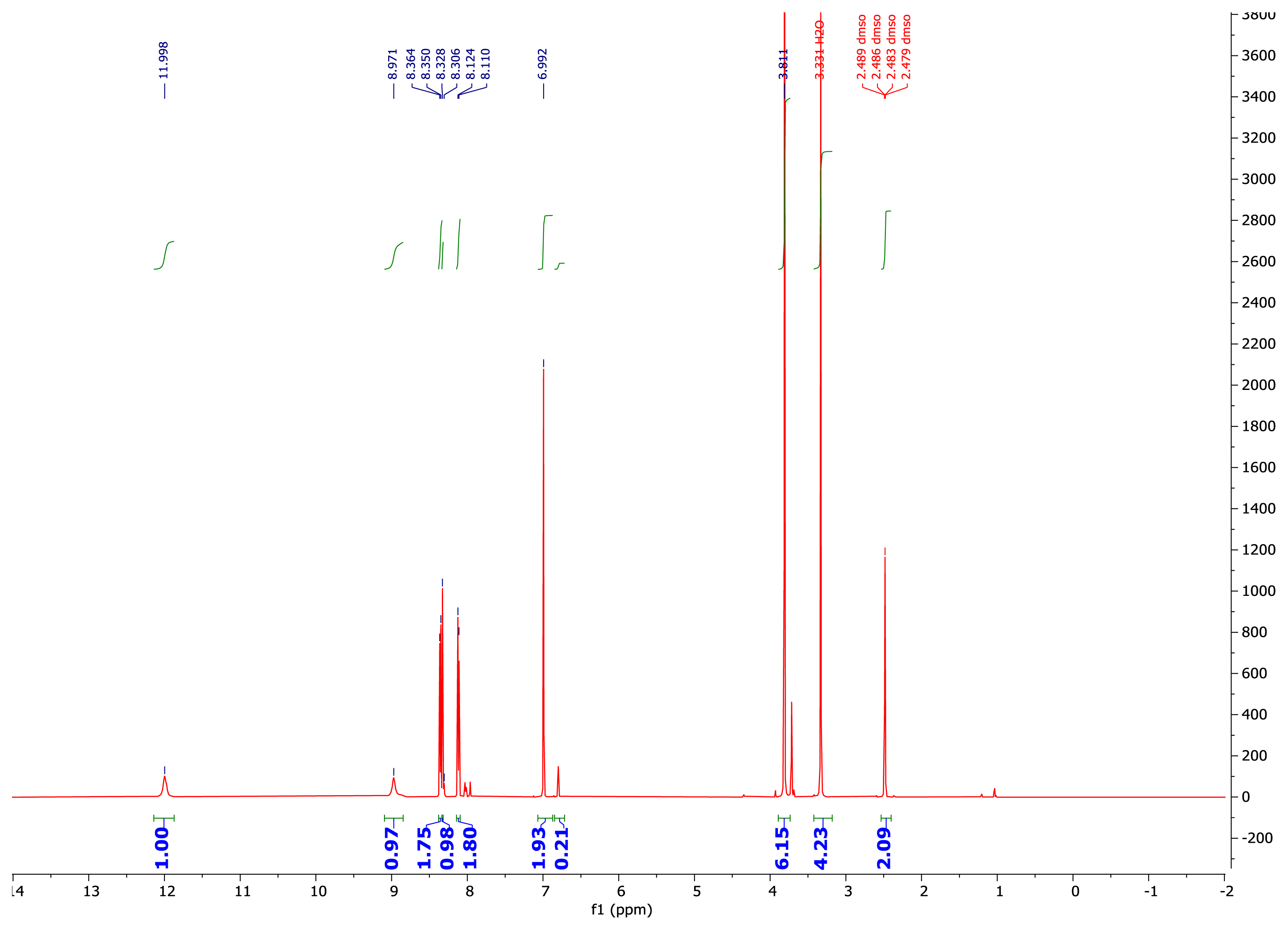

Supplement: Fig. S21 — 1H NMR spectrum of compound 7f [file turkjchem-46-1-236s21.tif]

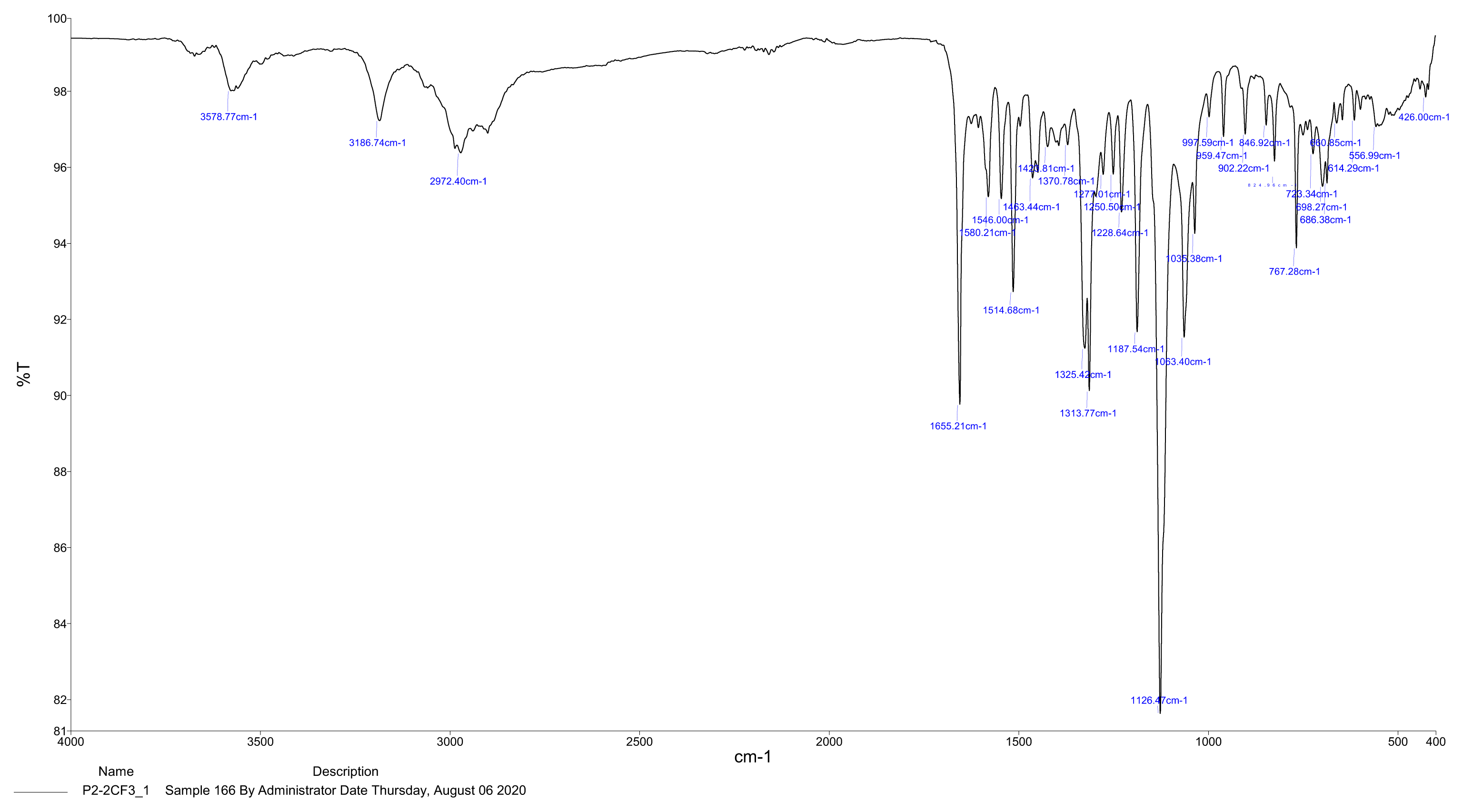

Supplement: Fig. S23 — FT-IR spectrum of compound 7g [file turkjchem-46-1-236s23.tif]

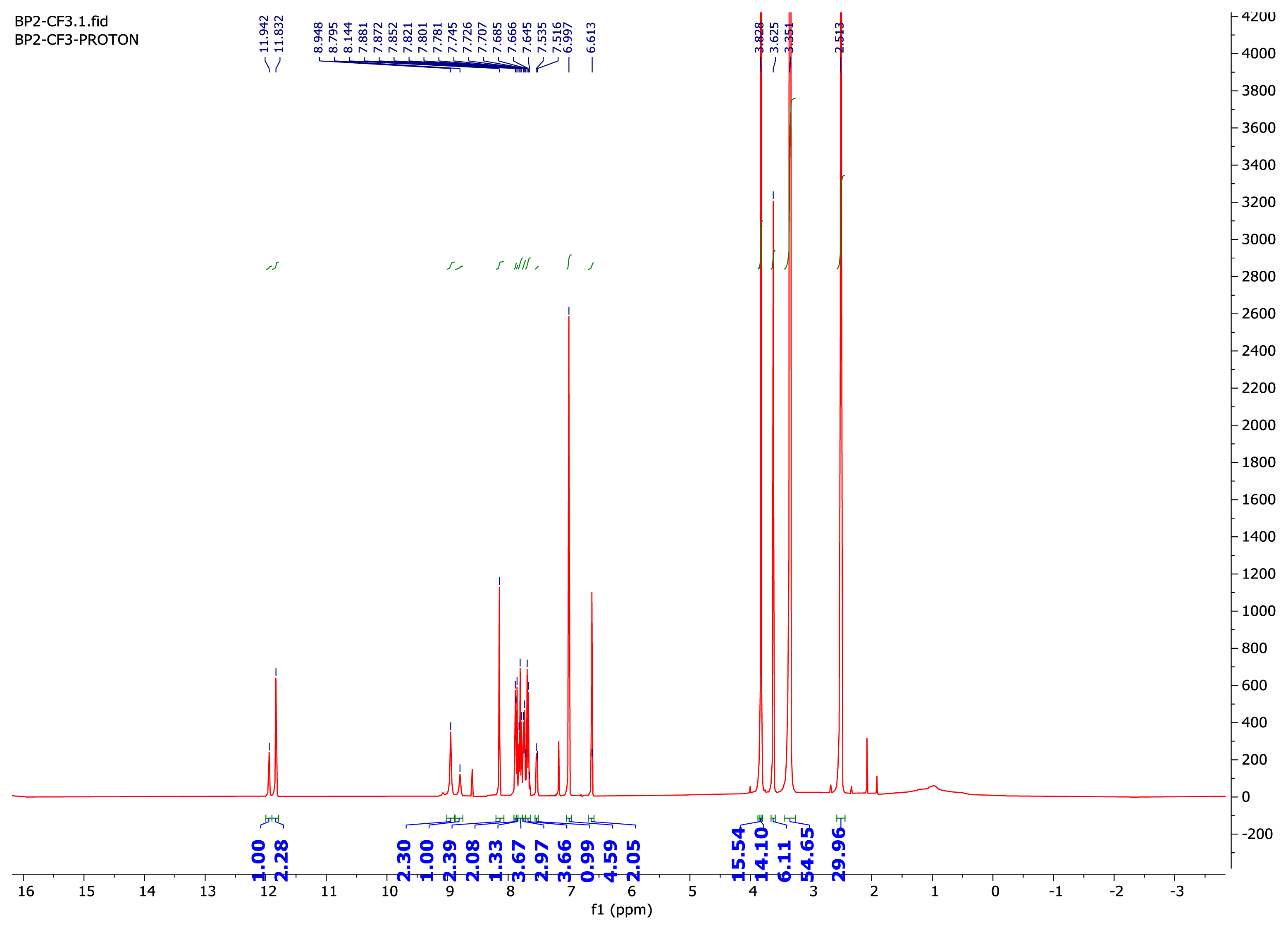

Supplement: Fig. S24 — 1H NMR spectrum of compound 7g [file turkjchem-46-1-236s24.tif]

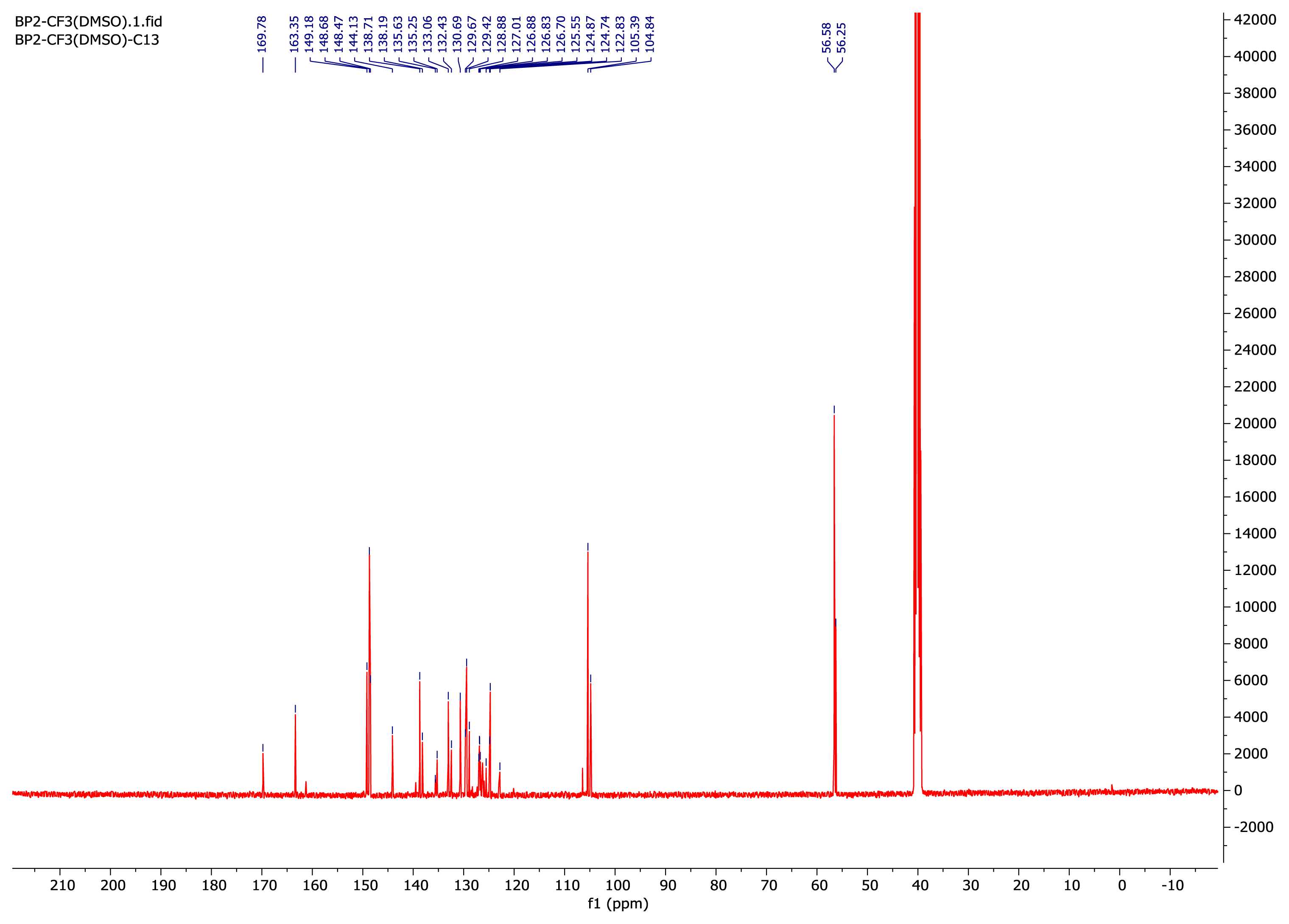

Supplement: Fig. S25 — 13C NMR spectrum of compound 7g [file turkjchem-46-1-236s25.tif]

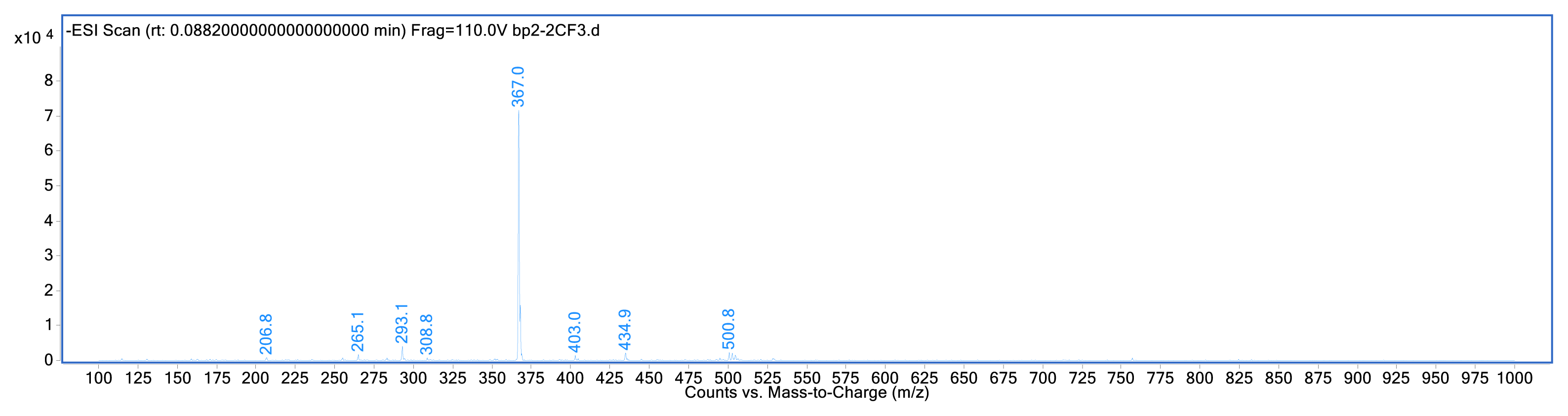

Supplement: Fig. S26 — LC-MS/MS spectrum of compound 7g [file turkjchem-46-1-236s26.tif]

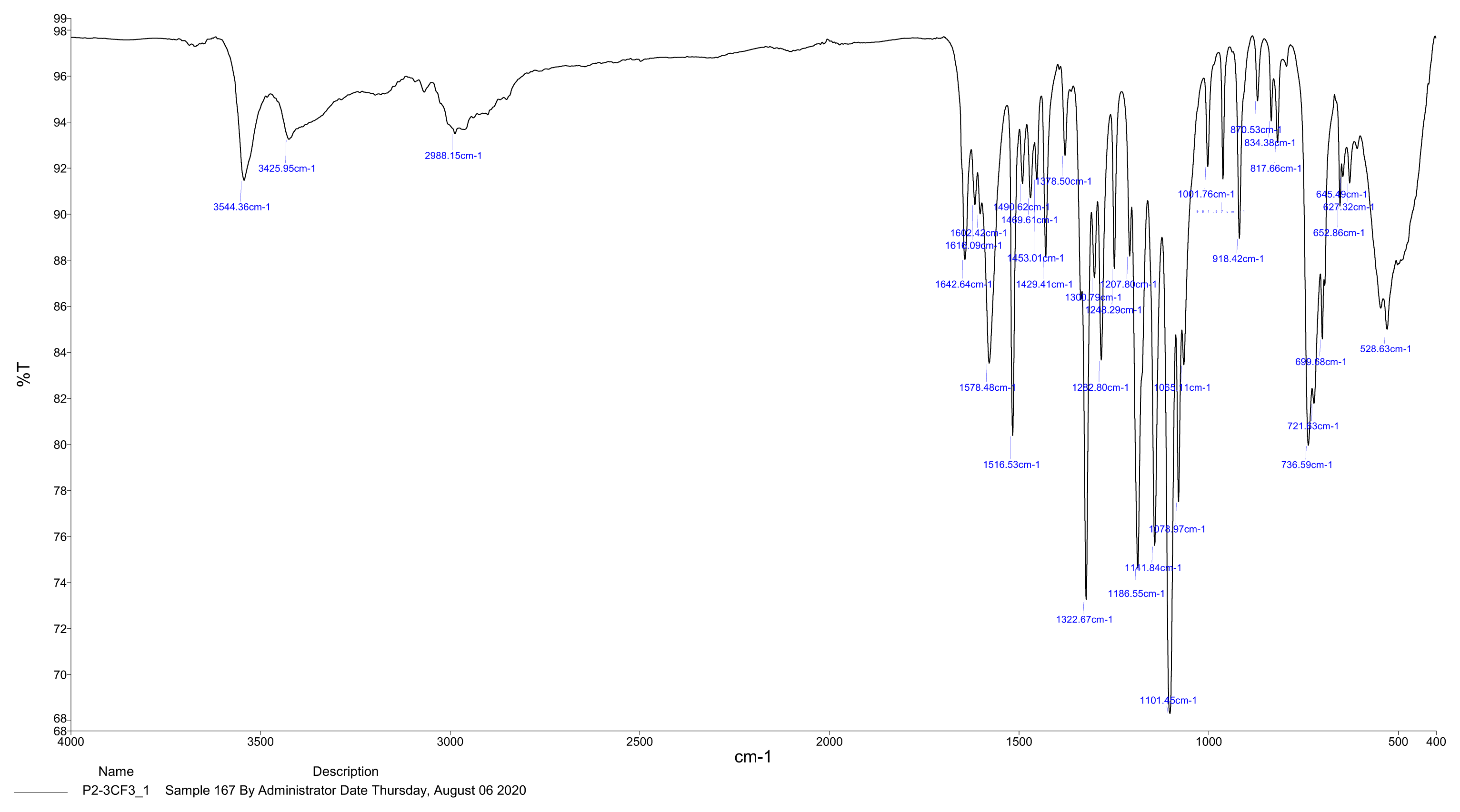

Supplement: Fig. S27 — FT-IR spectrum of compound 7h [file turkjchem-46-1-236s27.tif]

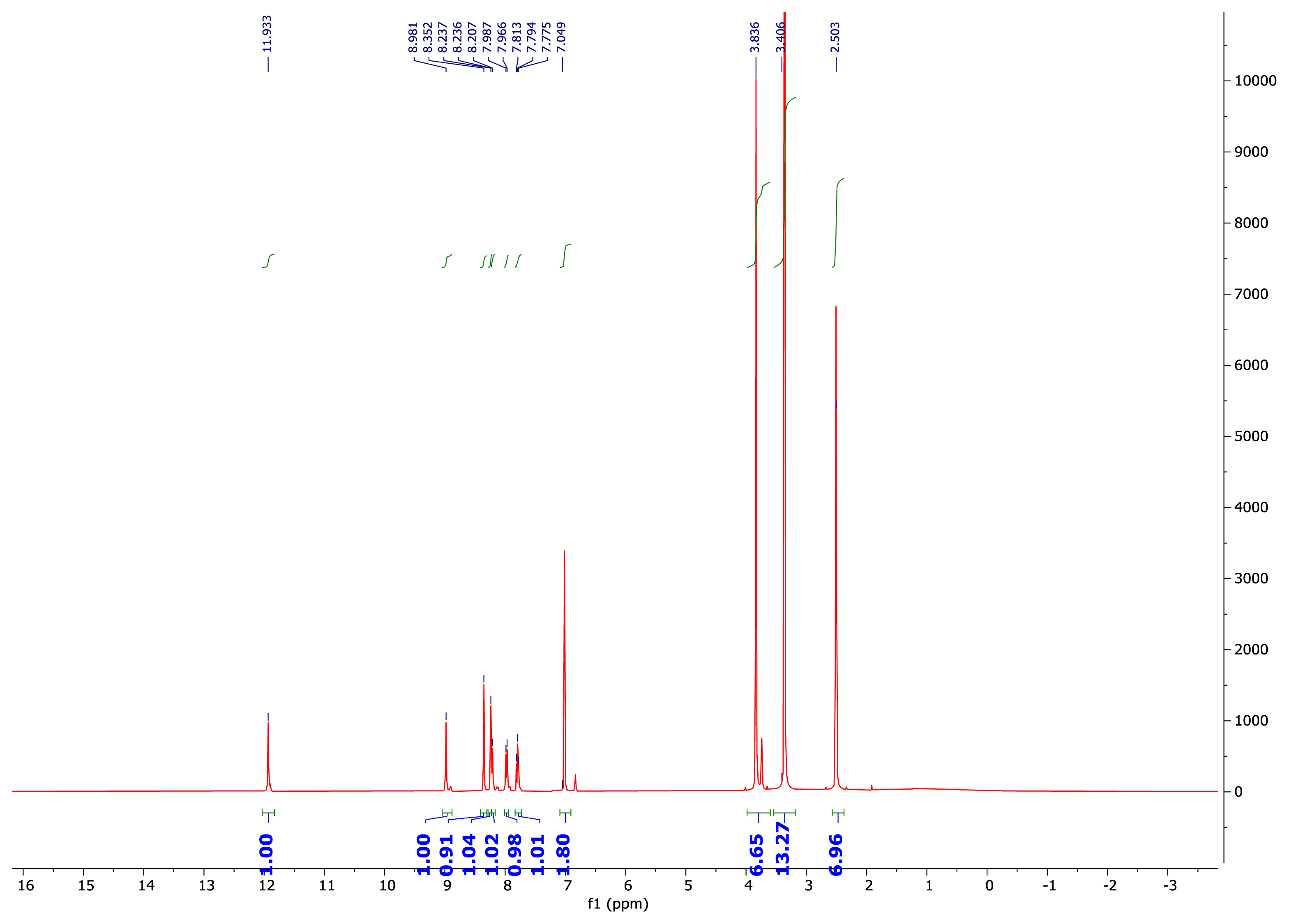

Supplement: Fig. S28 — 1H NMR spectrum of compound 7h [file turkjchem-46-1-236s28.tif]

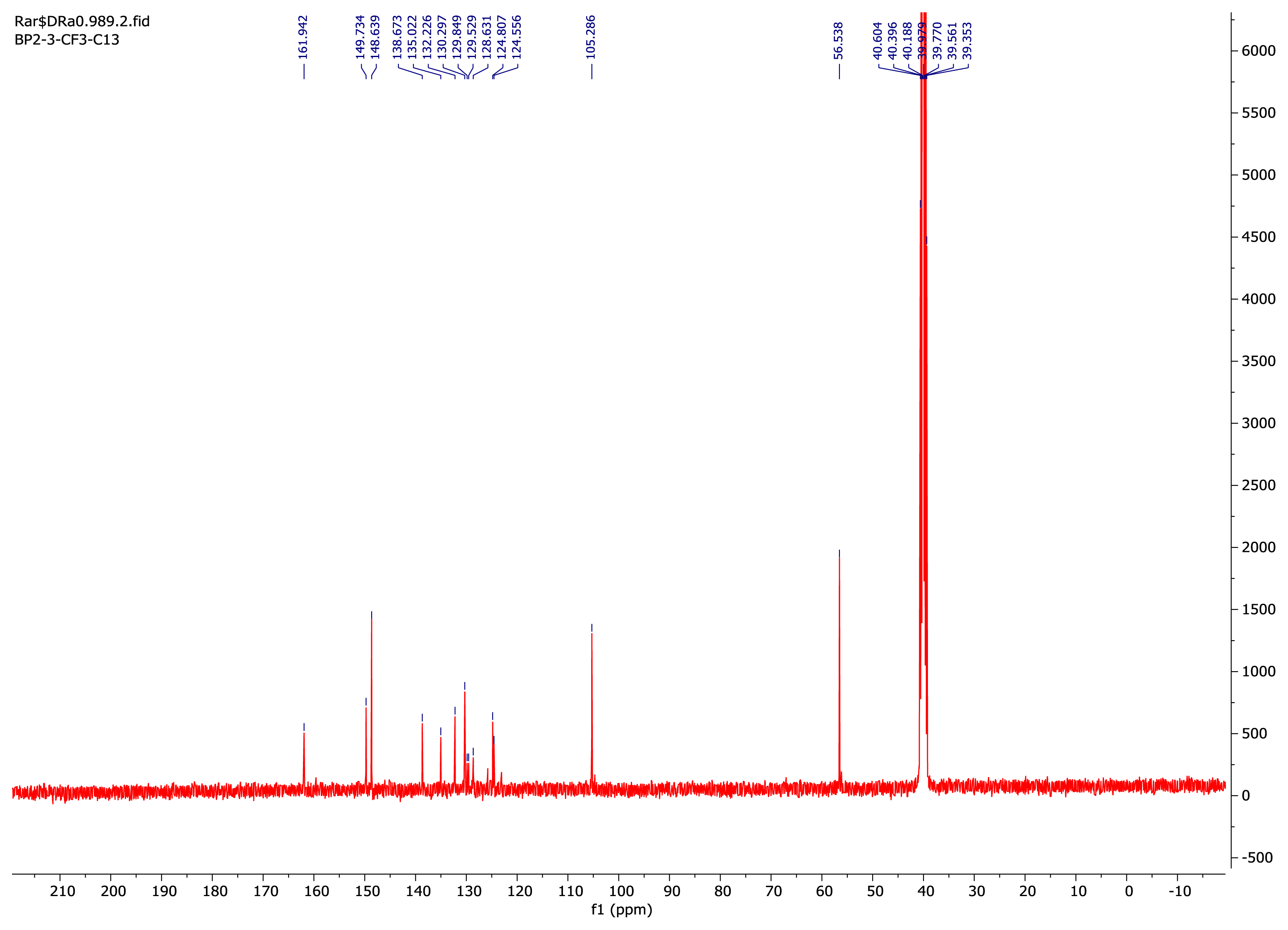

Supplement: Fig. S29 — 13C NMR spectrum of compound 7h [file turkjchem-46-1-236s29.tif]

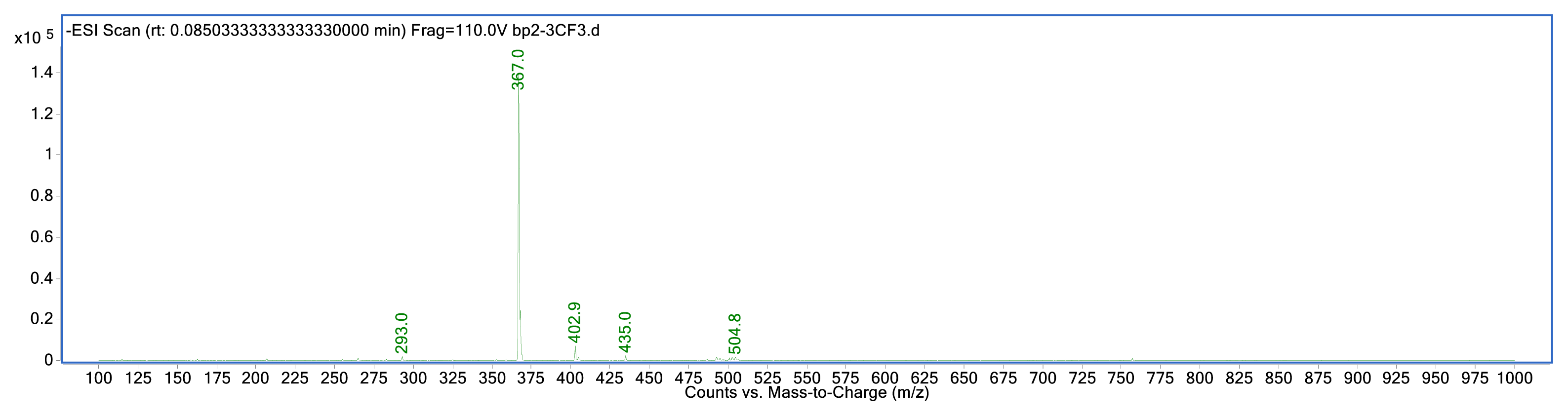

Supplement: Fig. S30 — LC-MS/MS spectrum of compound 7h [file turkjchem-46-1-236s30.tif]

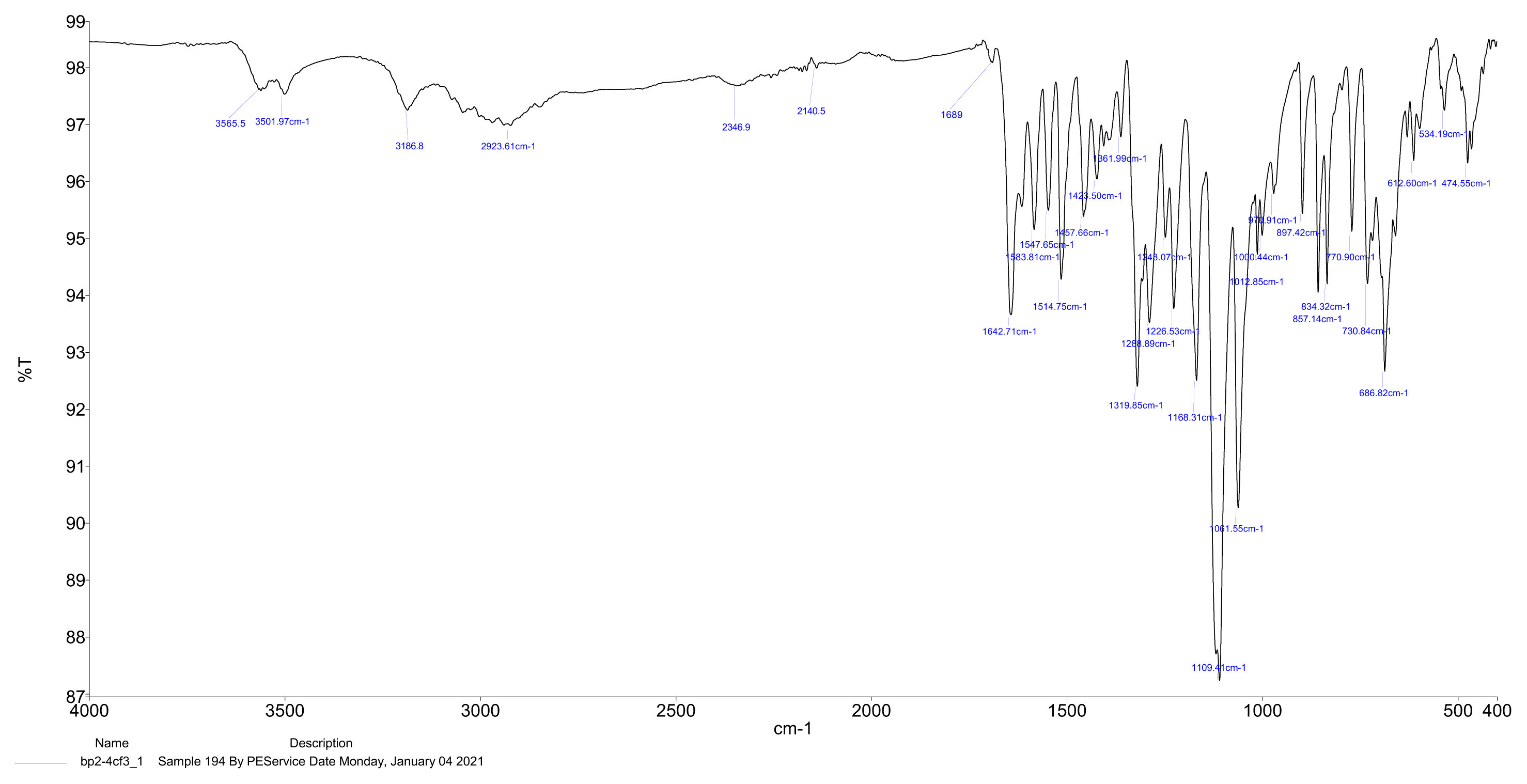

Supplement: Fig. S31 — FT-IR spectrum of compound 7i [file turkjchem-46-1-236s31.tif]

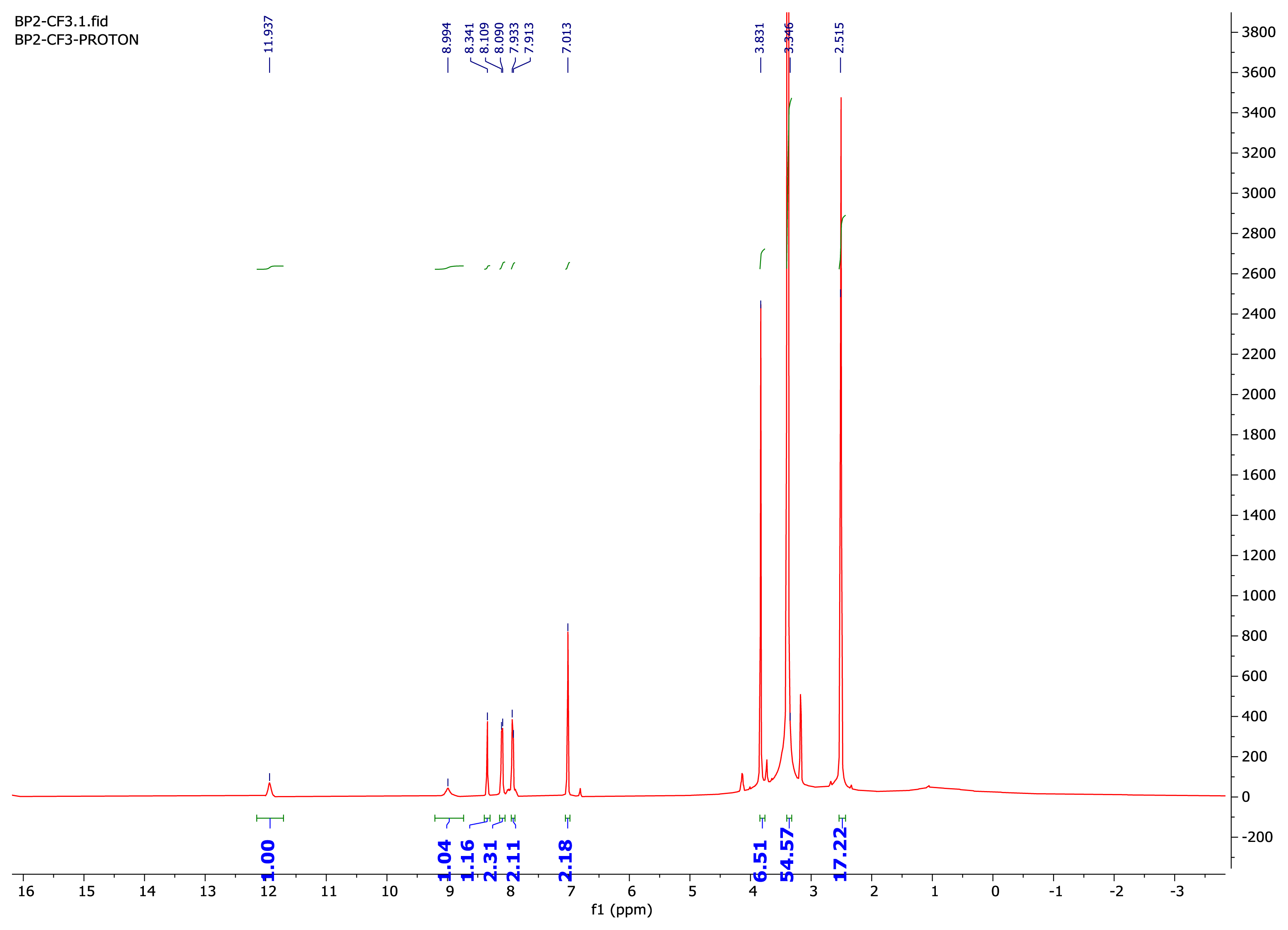

Supplement: Fig. S32 — 1H NMR spectrum of compound 7i [file turkjchem-46-1-236s32.tif]

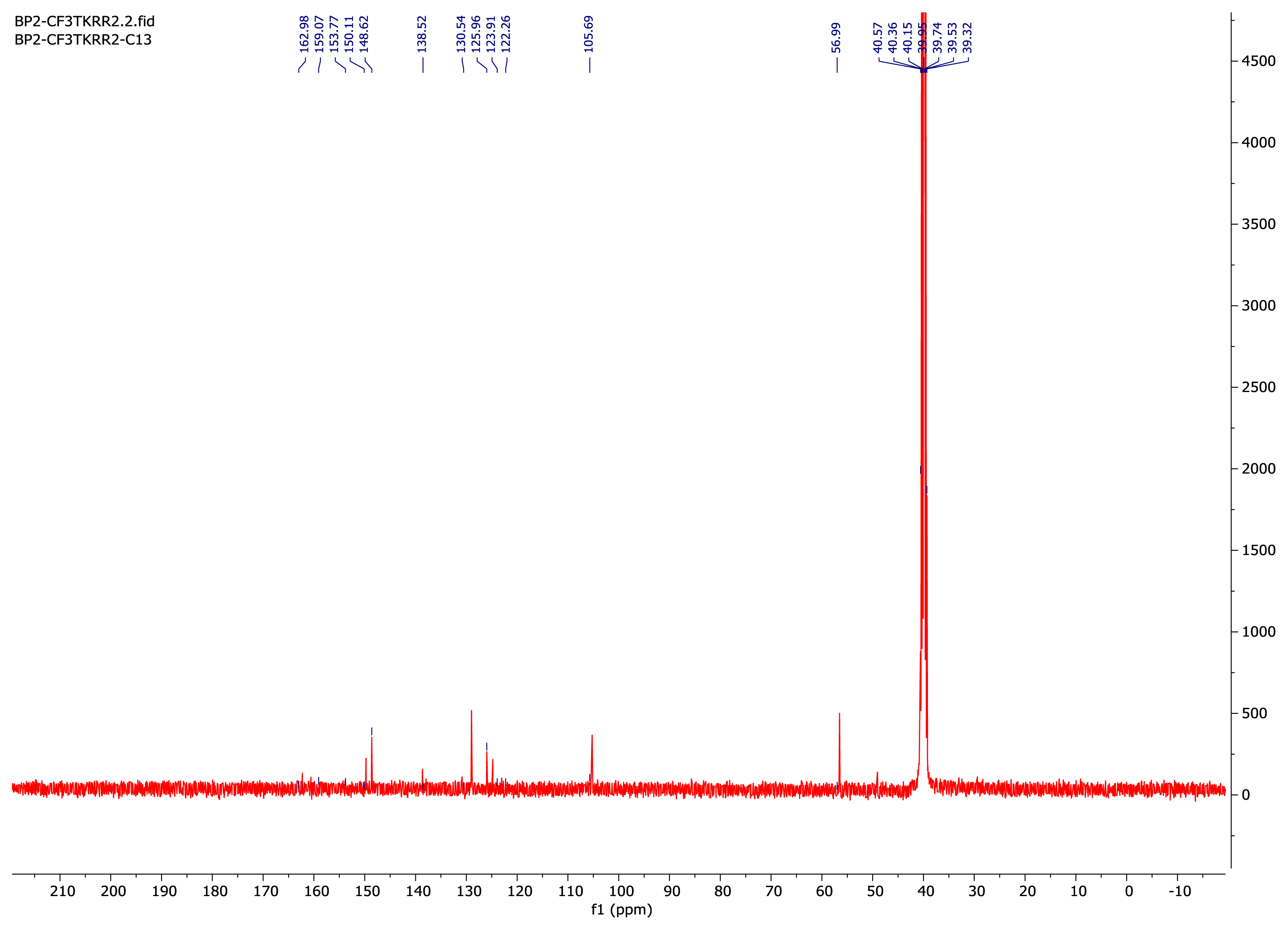

Supplement: Fig. S33 — 13C NMR spectrum of compound 7i [file turkjchem-46-1-236s33.tif]

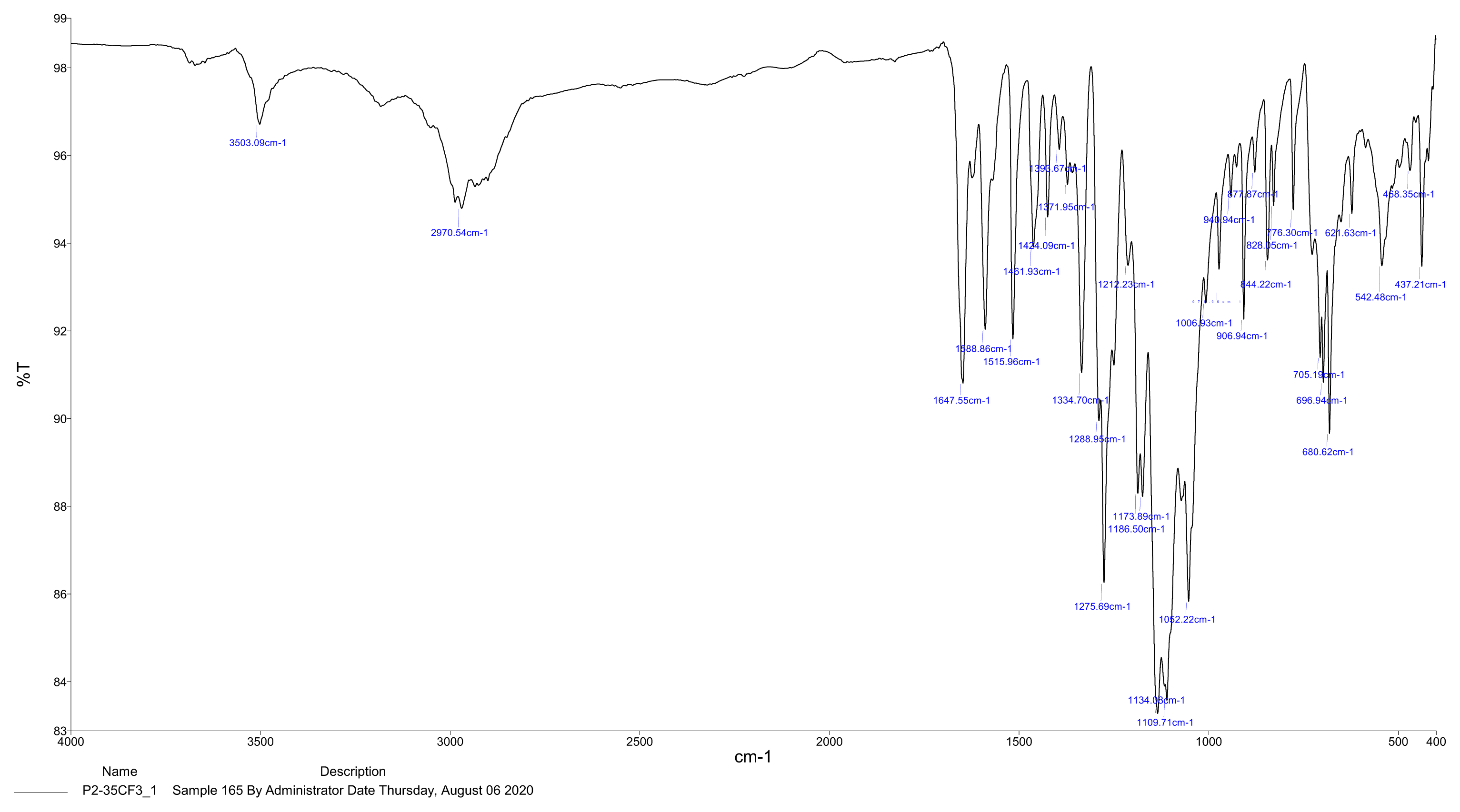

Supplement: Fig. S34 — FT-IR spectrum of compound 7j [file turkjchem-46-1-236s34.tif]

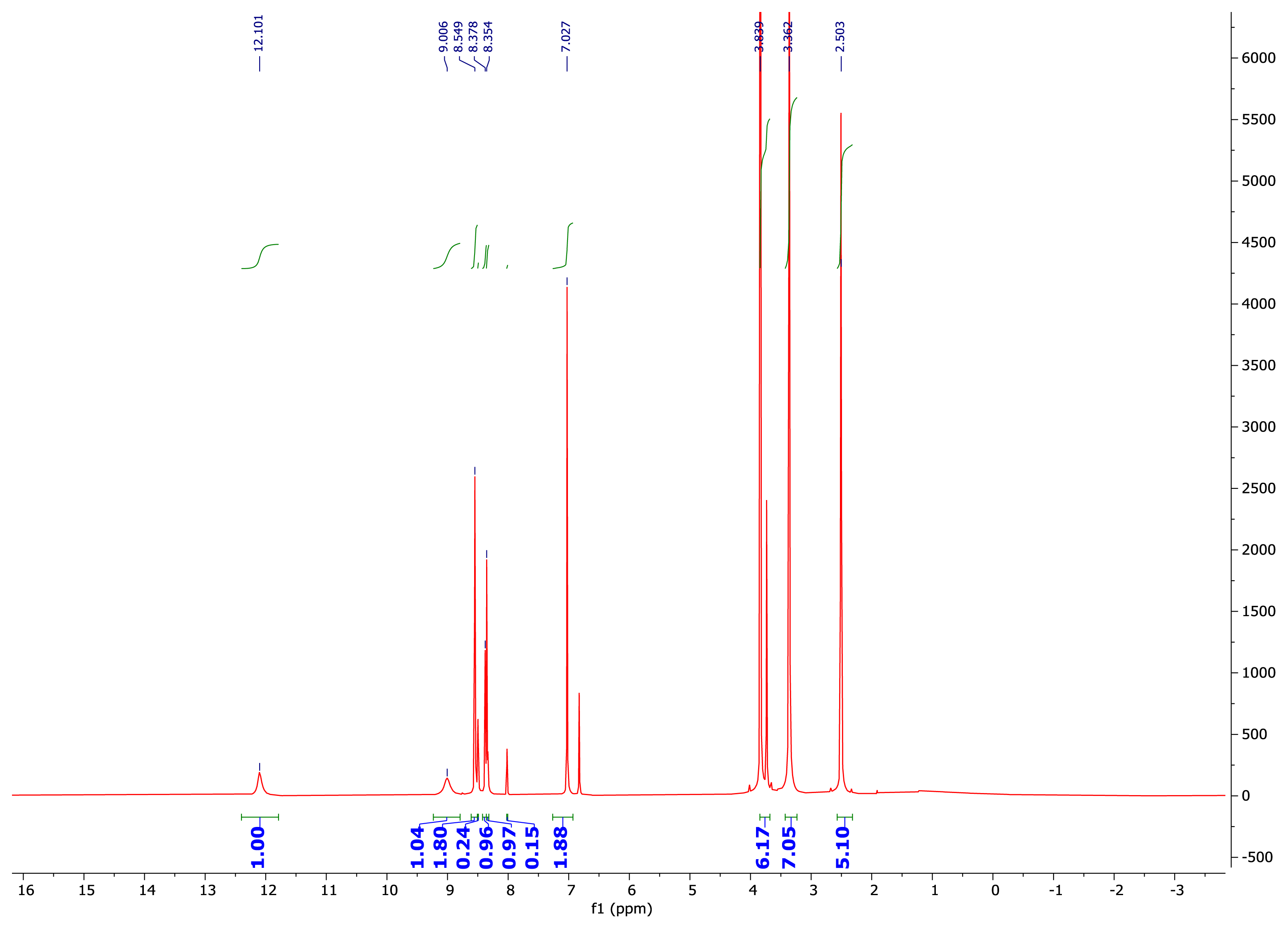

Supplement: Fig. S35 — 1H NMR spectrum of compound 7j [file turkjchem-46-1-236s35.tif]

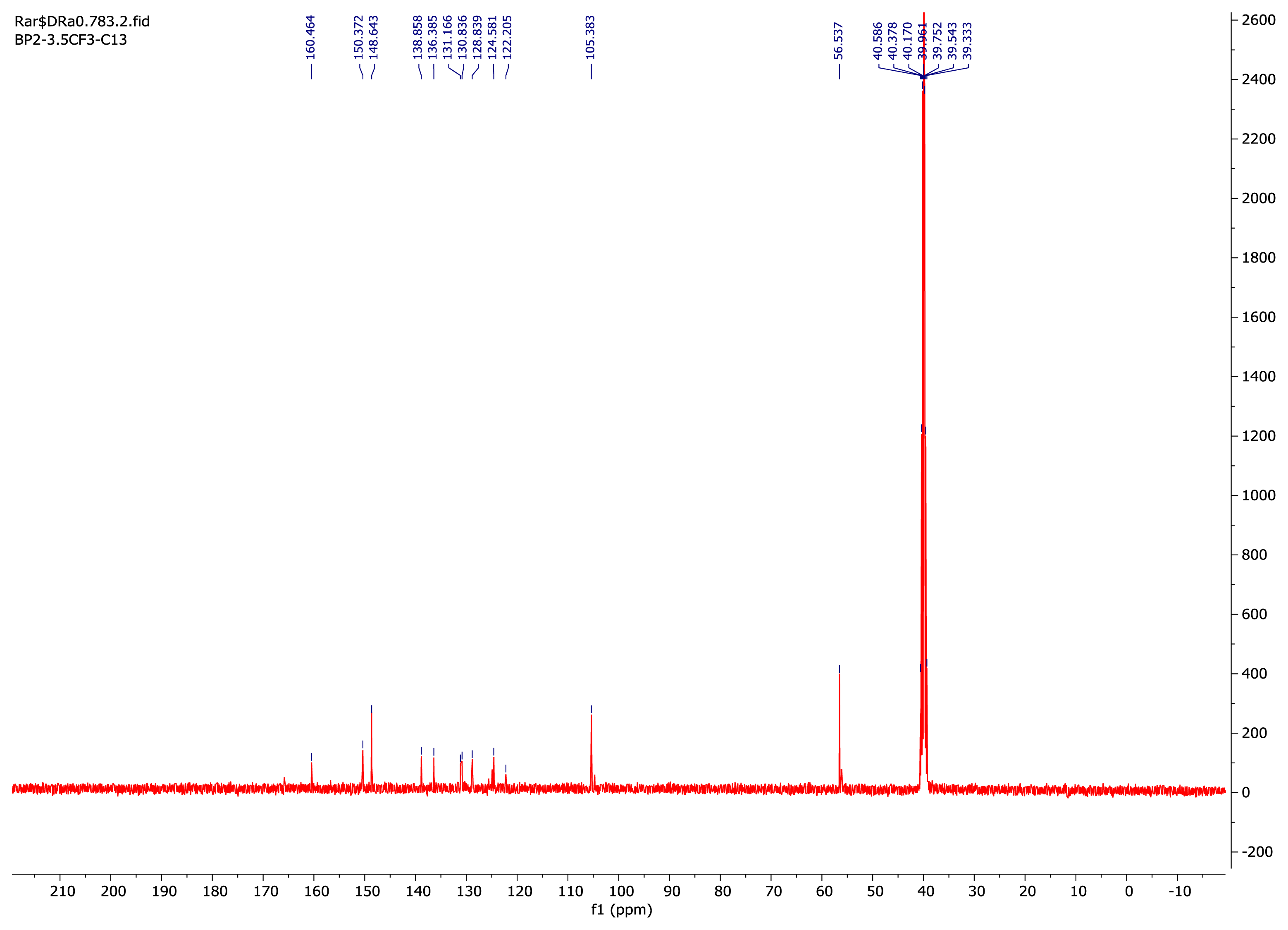

Supplement: Fig. S36 — 13C NMR spectrum of compound 7j [file turkjchem-46-1-236s36.tif]

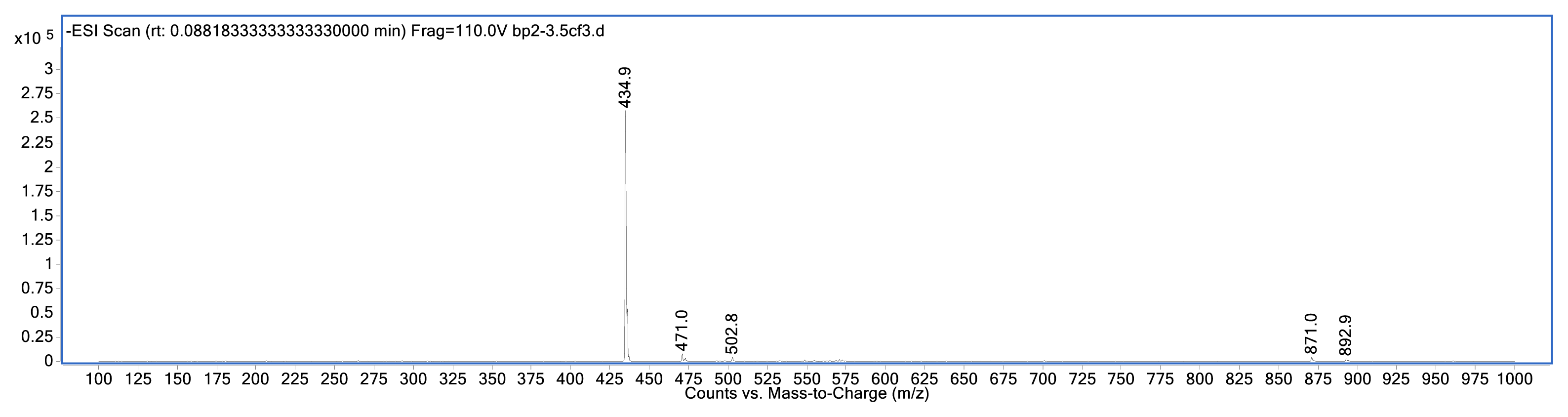

Supplement: Fig. S37 — LC-MS/MS spectrum of compound 7j [file turkjchem-46-1-236s37.tif]

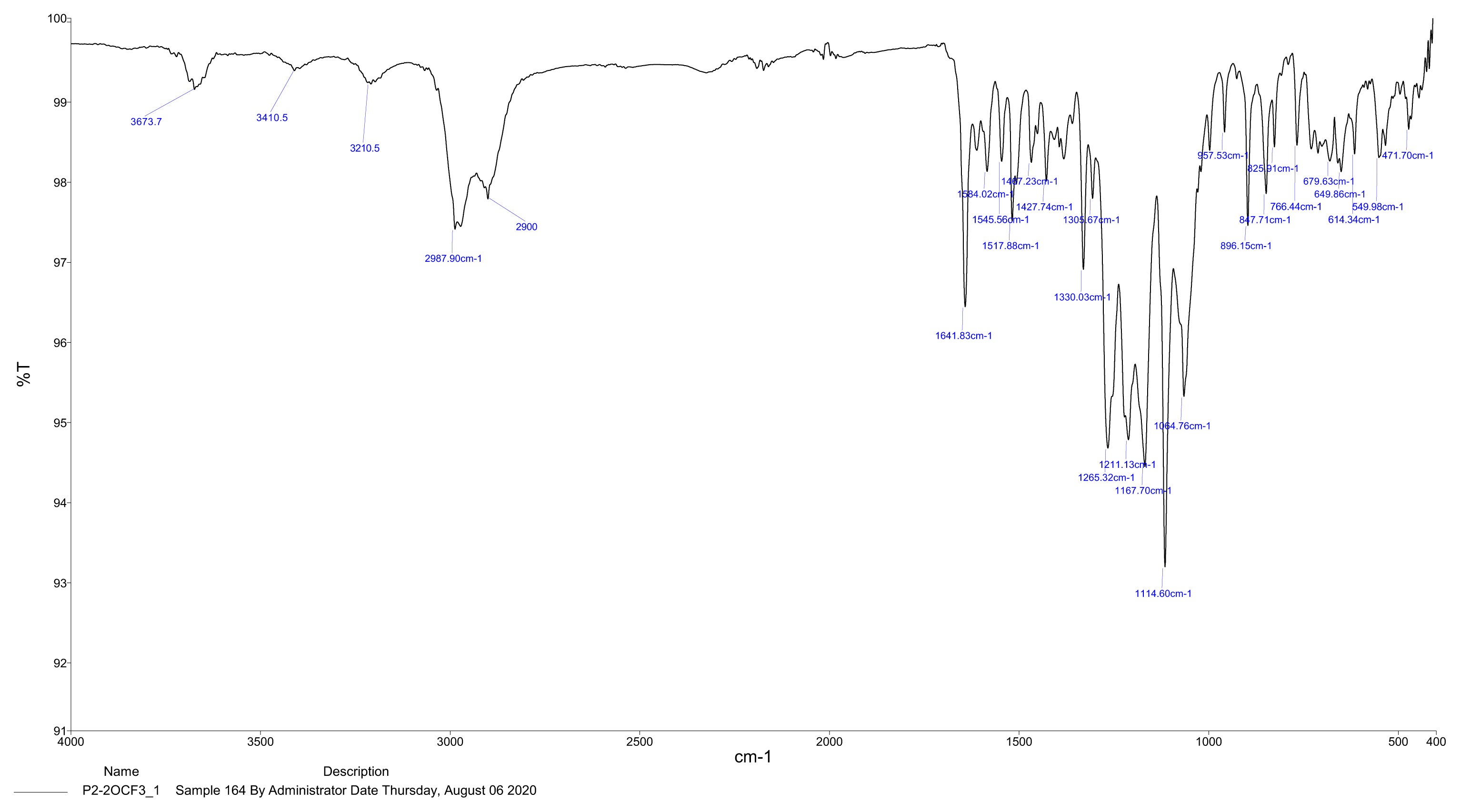

Supplement: Fig. S38 — FT-IR spectrum of compound 7k [file turkjchem-46-1-236s38.tif]

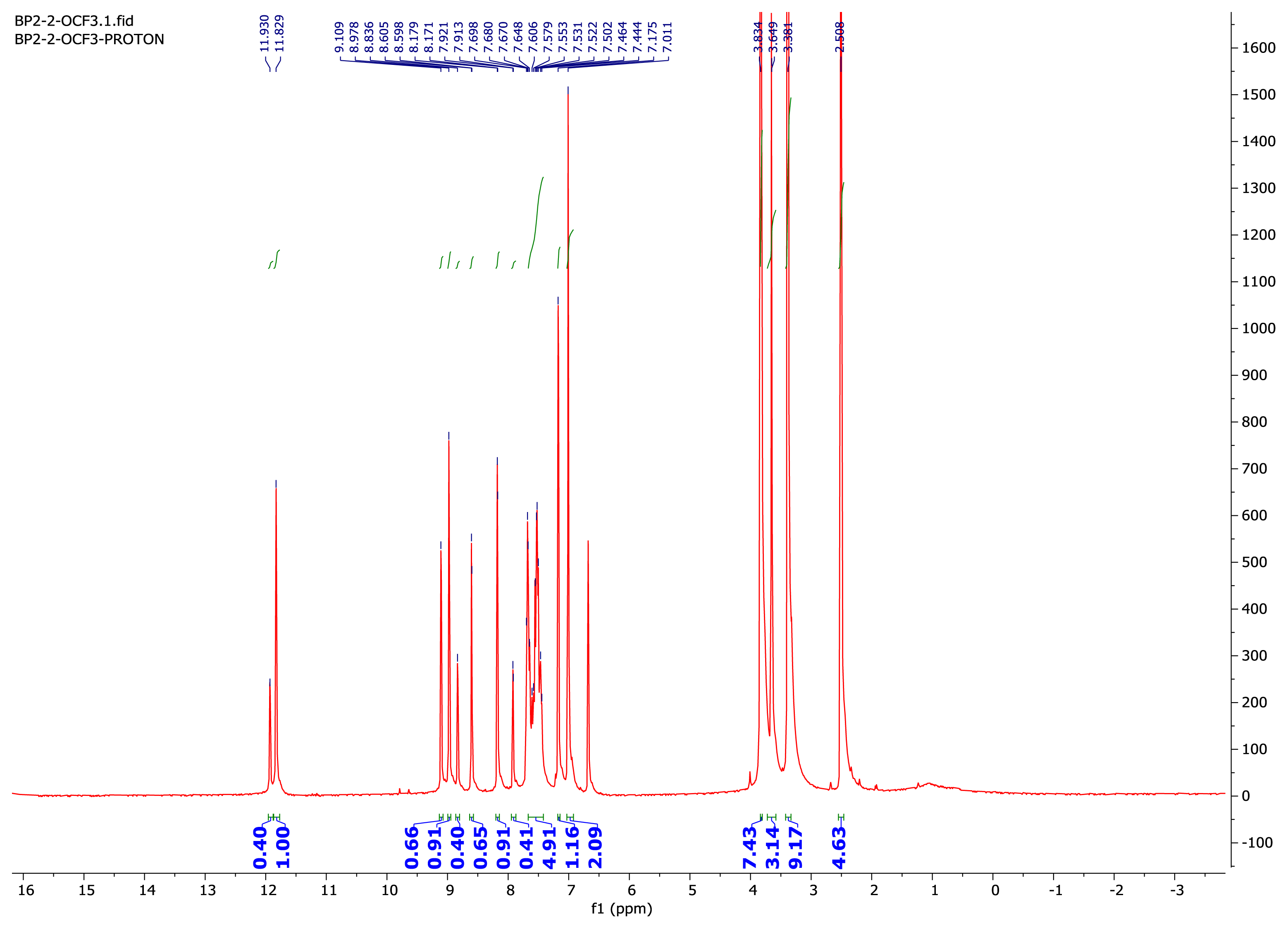

Supplement: Fig. S39 — 1H NMR spectrum of compound 7k [file turkjchem-46-1-236s39.tif]

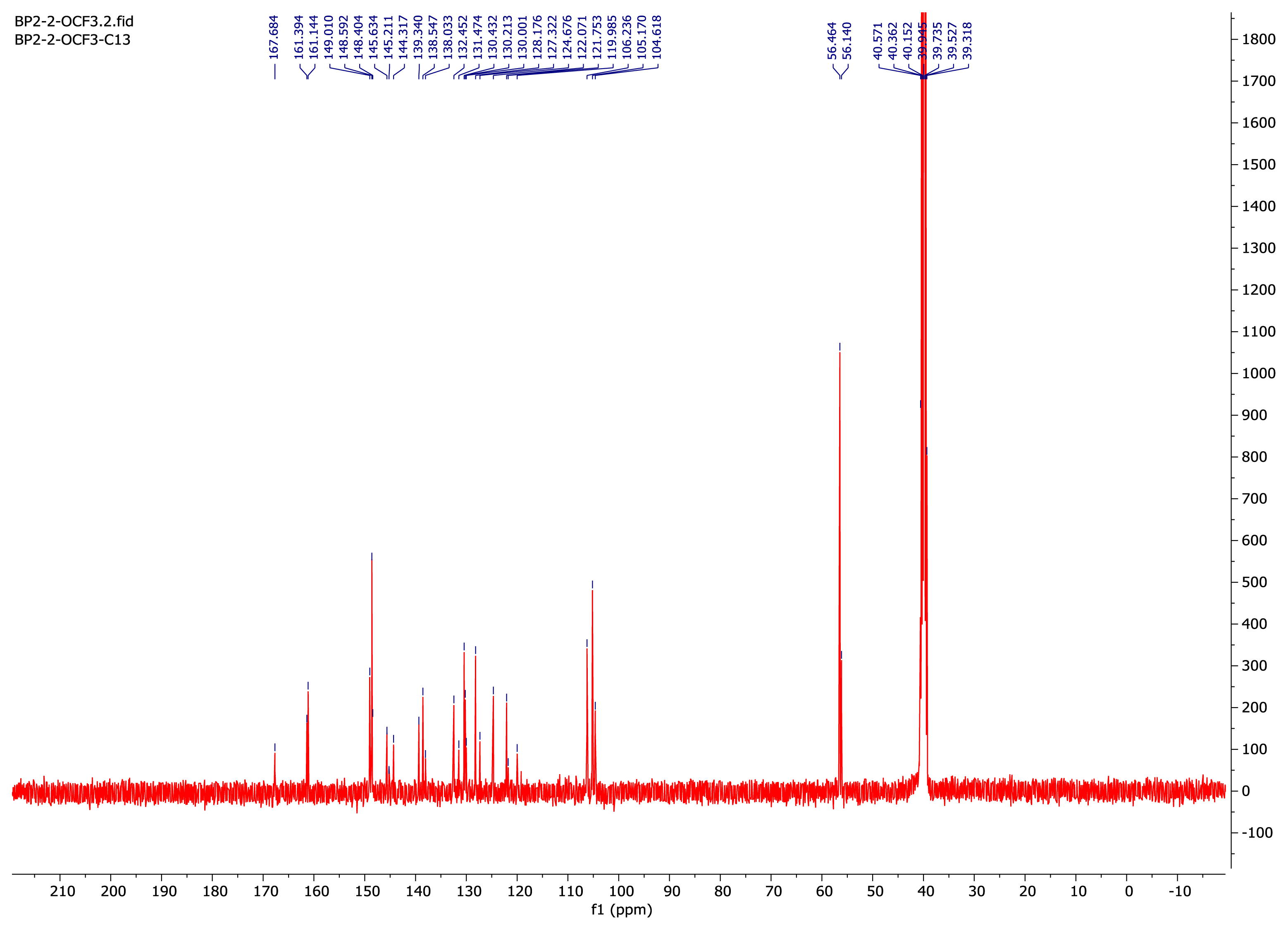

Supplement: Fig. S40 — 13C NMR spectrum of compound 7k [file turkjchem-46-1-236s40.tif]

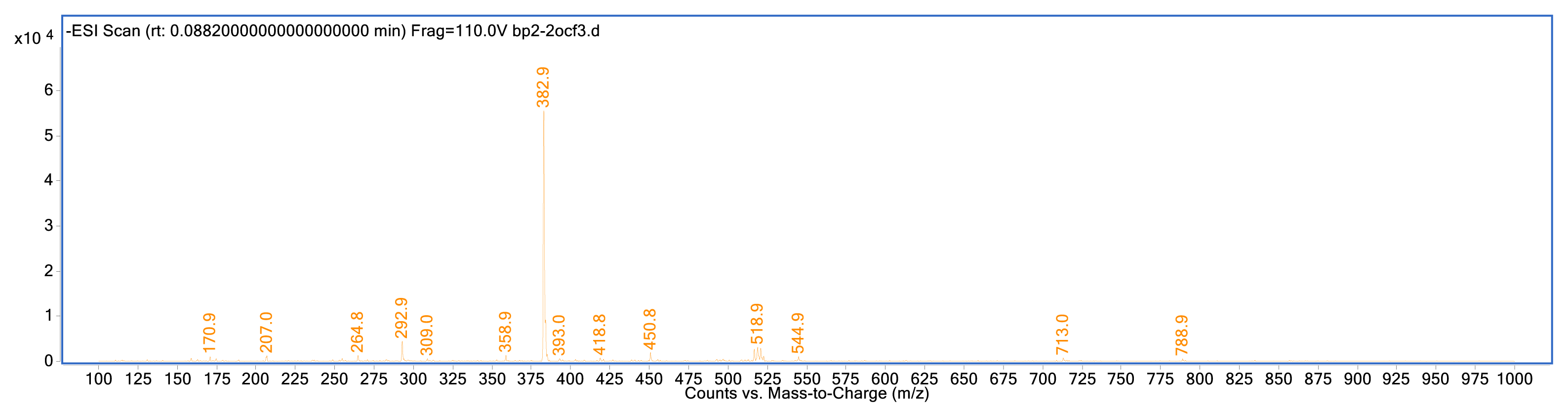

Supplement: Fig. S41 — LC-MS/MS spectrum of compound 7k [file turkjchem-46-1-236s41.tif]

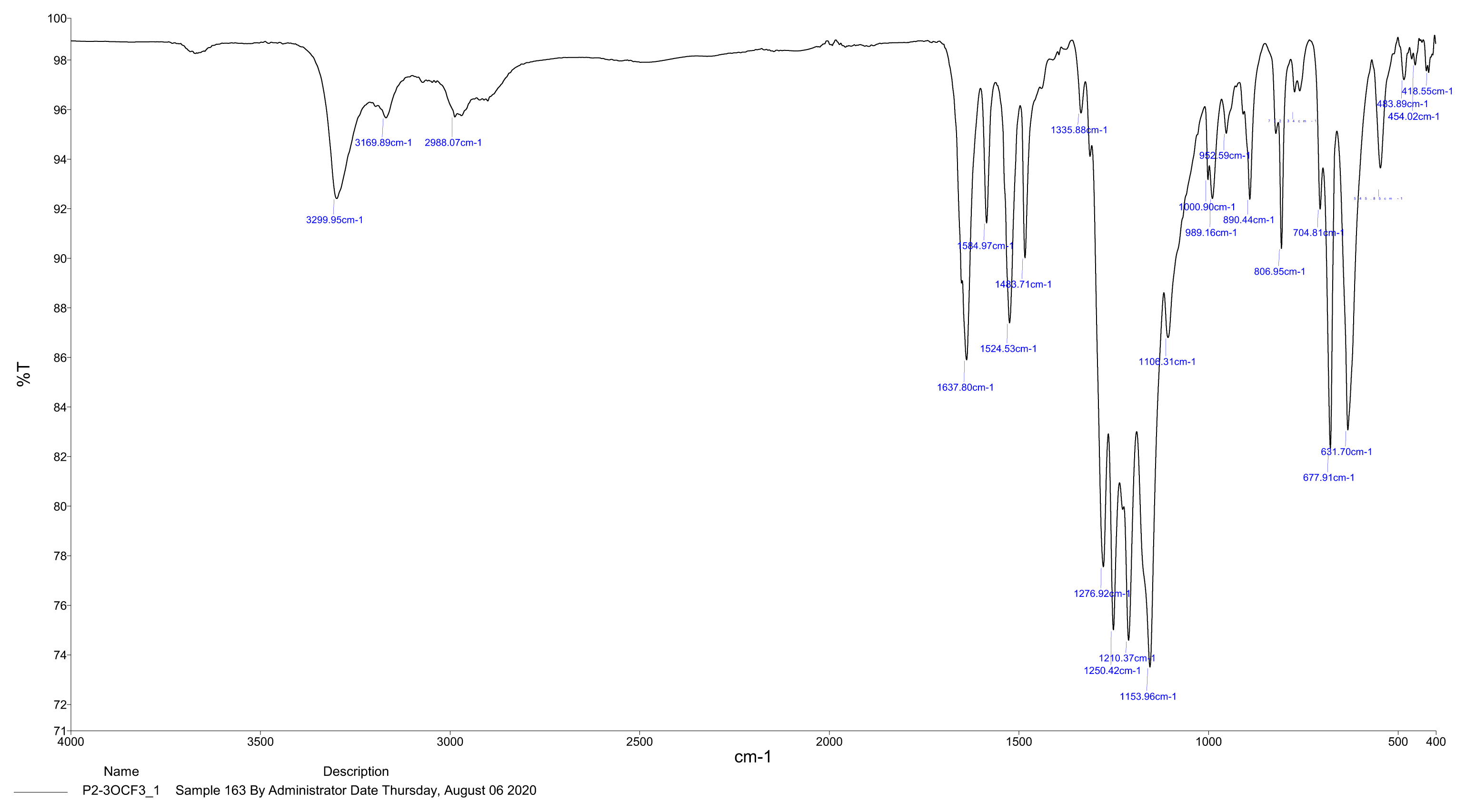

Supplement: Fig. S42 — FT-IR spectrum of compound 7l [file turkjchem-46-1-236s42.tif]

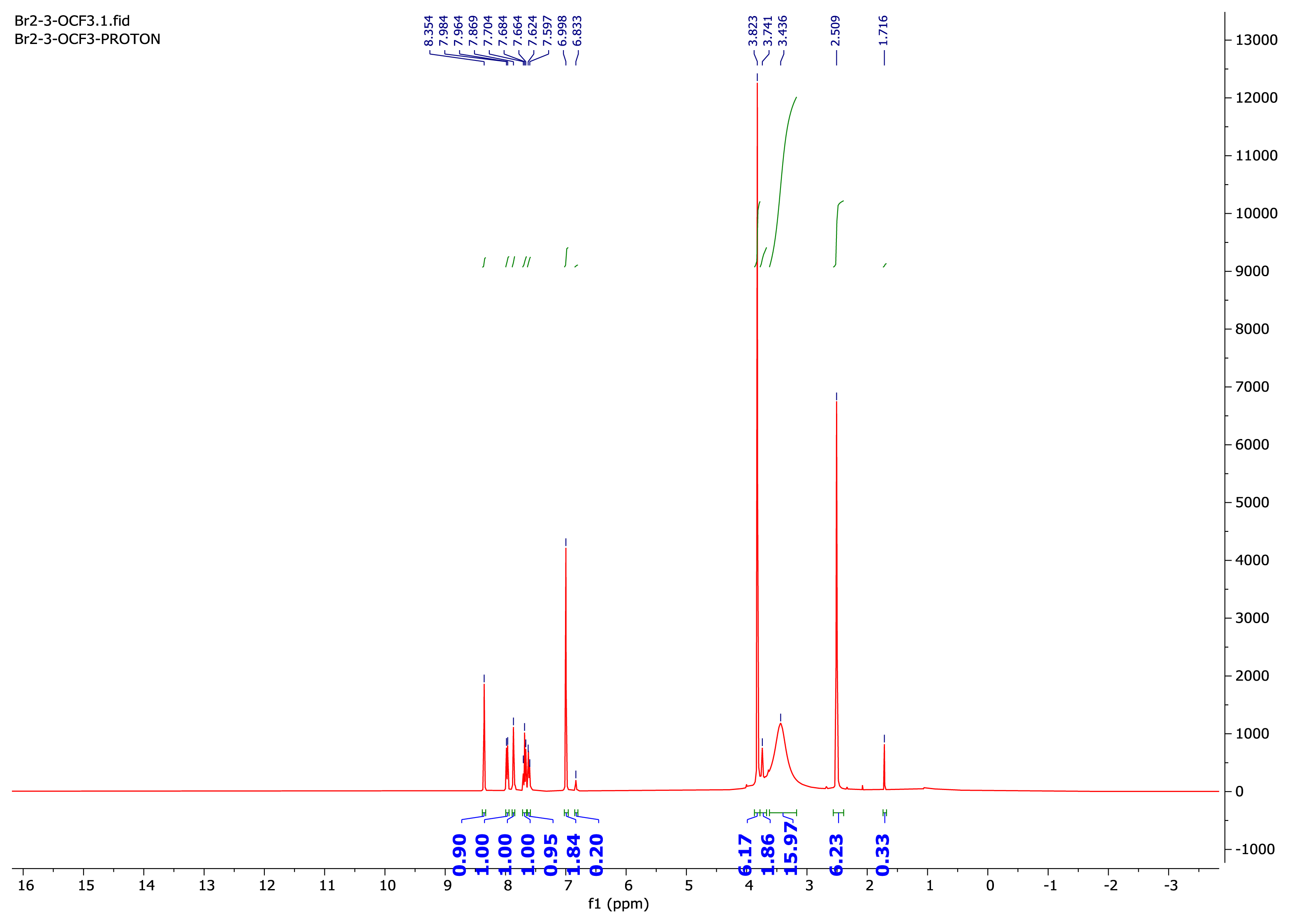

Supplement: Fig. S43 — 1H NMR spectrum of compound 7l [file turkjchem-46-1-236s43.tif]

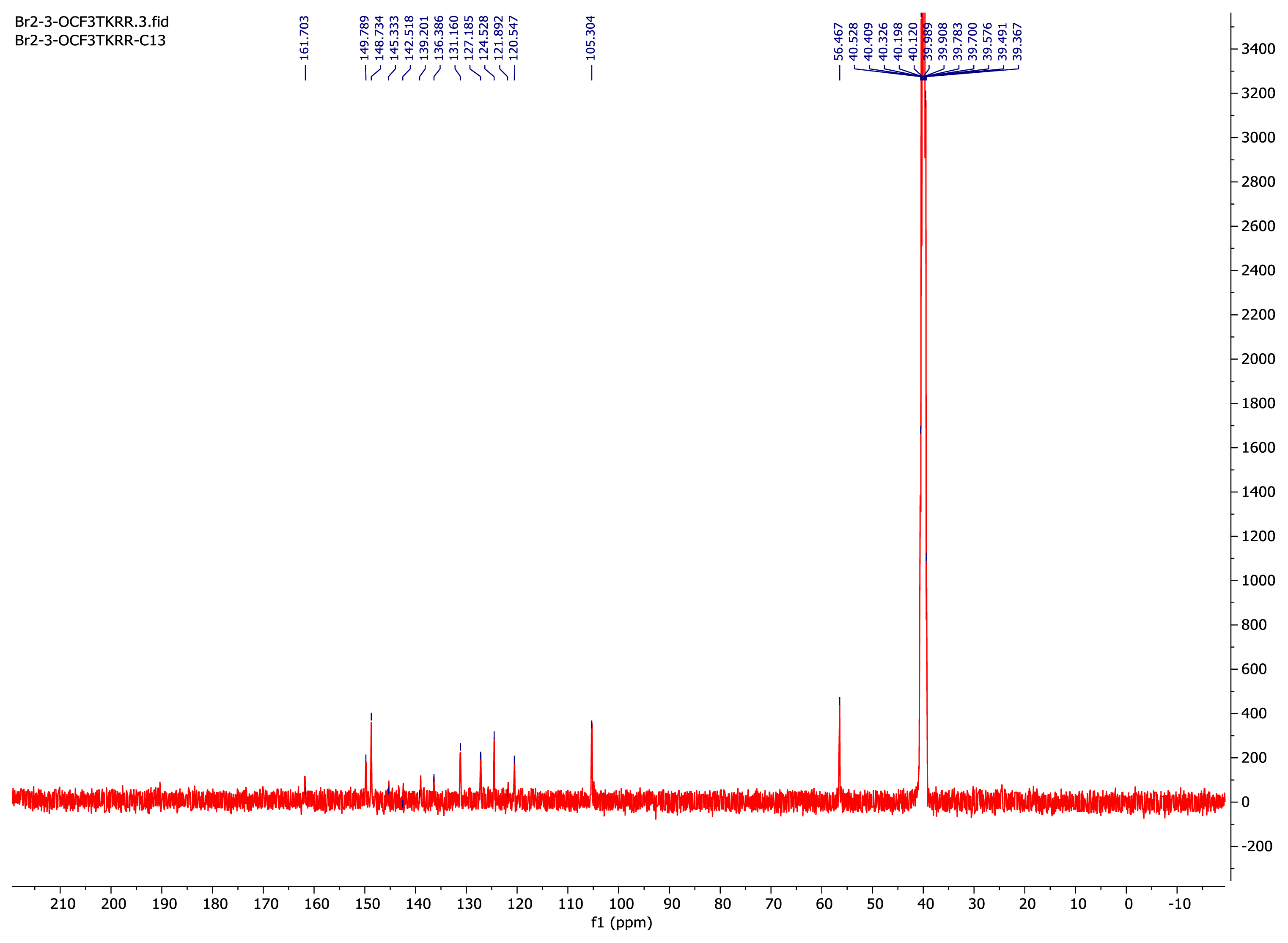

Supplement: Fig. S44 — 13C NMR spectrum of compound 7l [file turkjchem-46-1-236s44.tif]

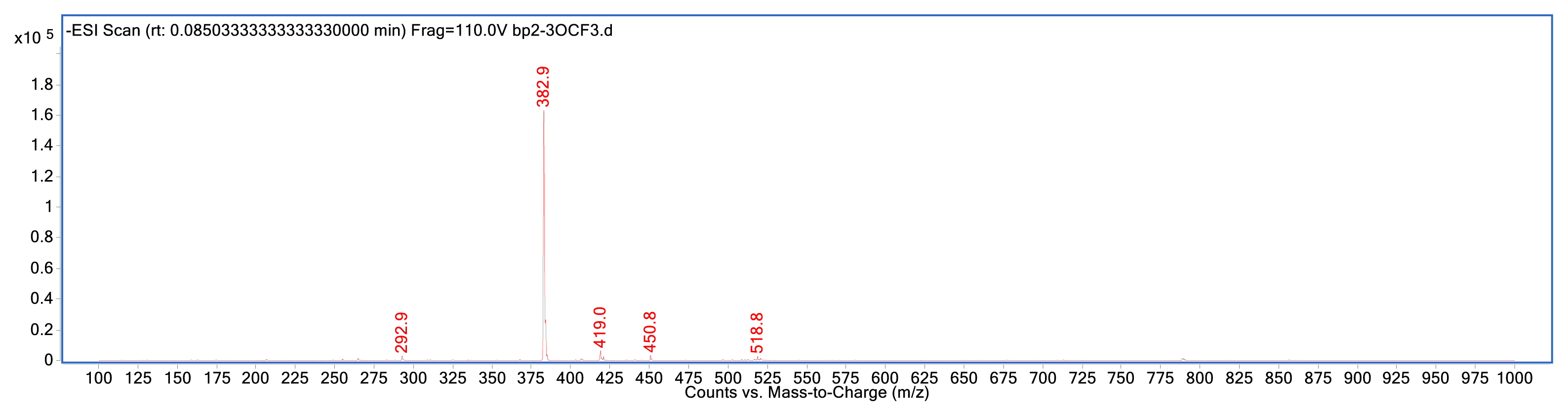

Supplement: Fig. S45 — LC-MS/MS spectrum of compound 7l [file turkjchem-46-1-236s45.tif]

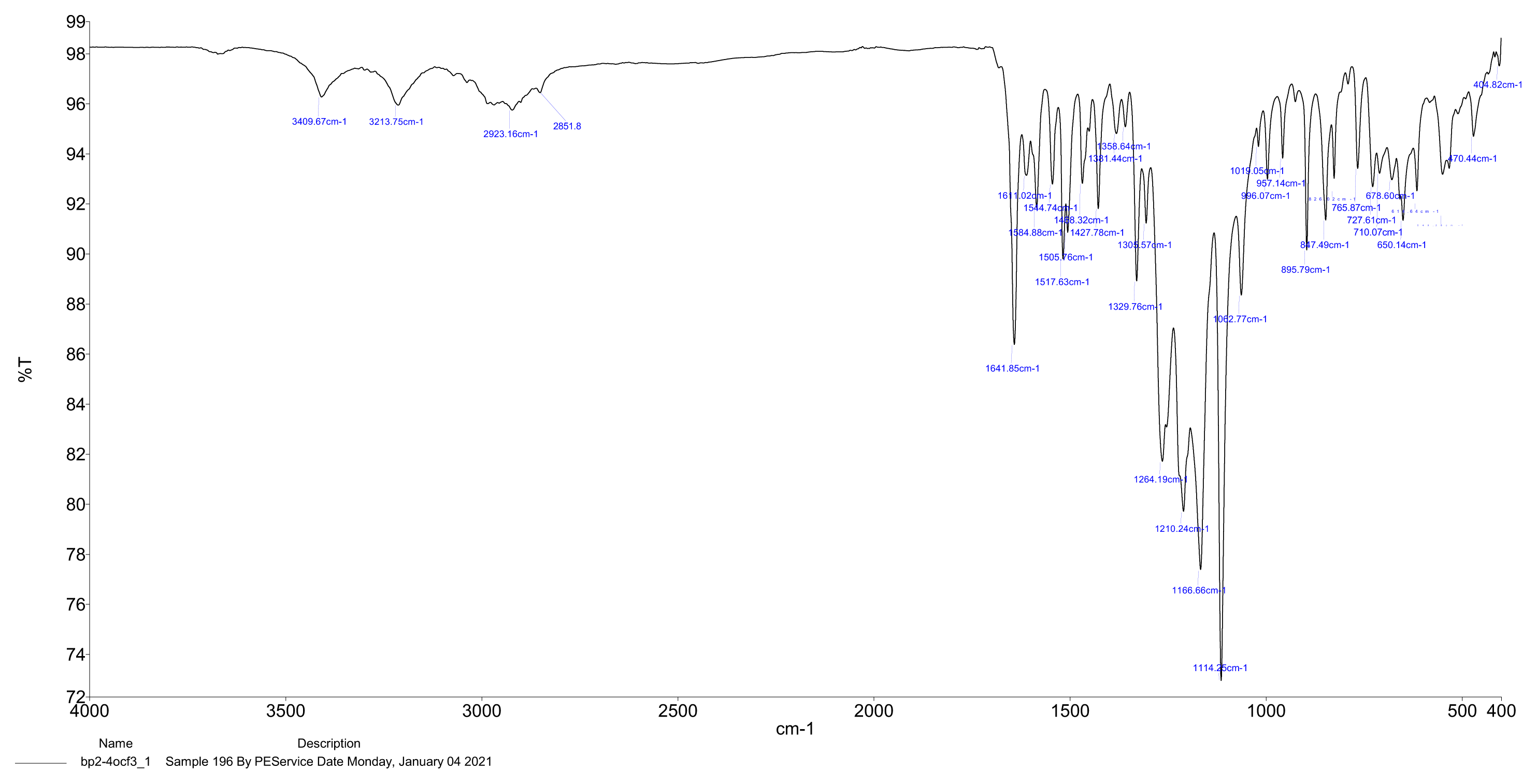

Supplement: Fig. S46 — FT-IR spectrum of compound 7m [file turkjchem-46-1-236s46.tif]

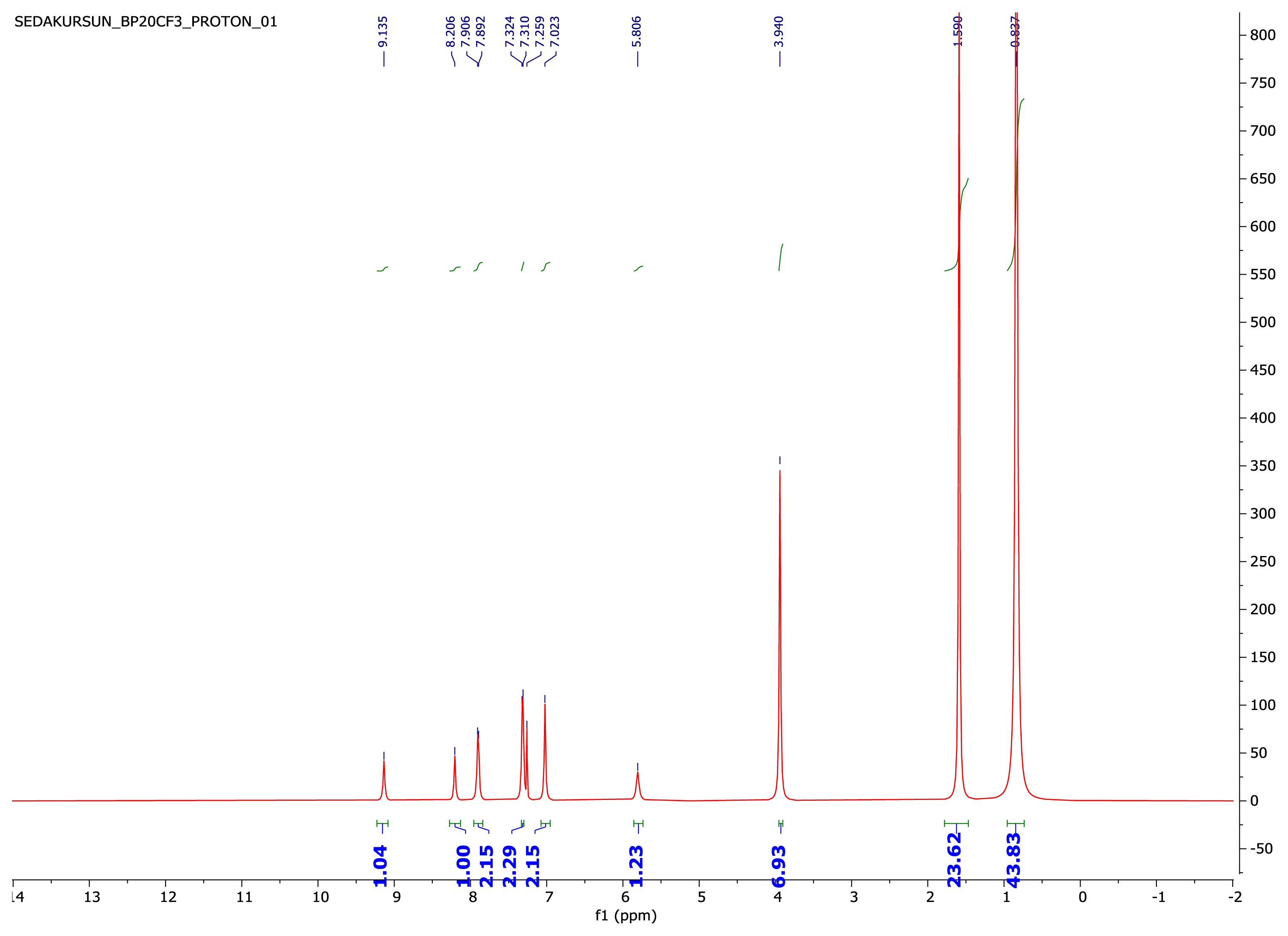

Supplement: Fig. S47 — 1H NMR spectrum of compound 7m [file turkjchem-46-1-236s47.tif]

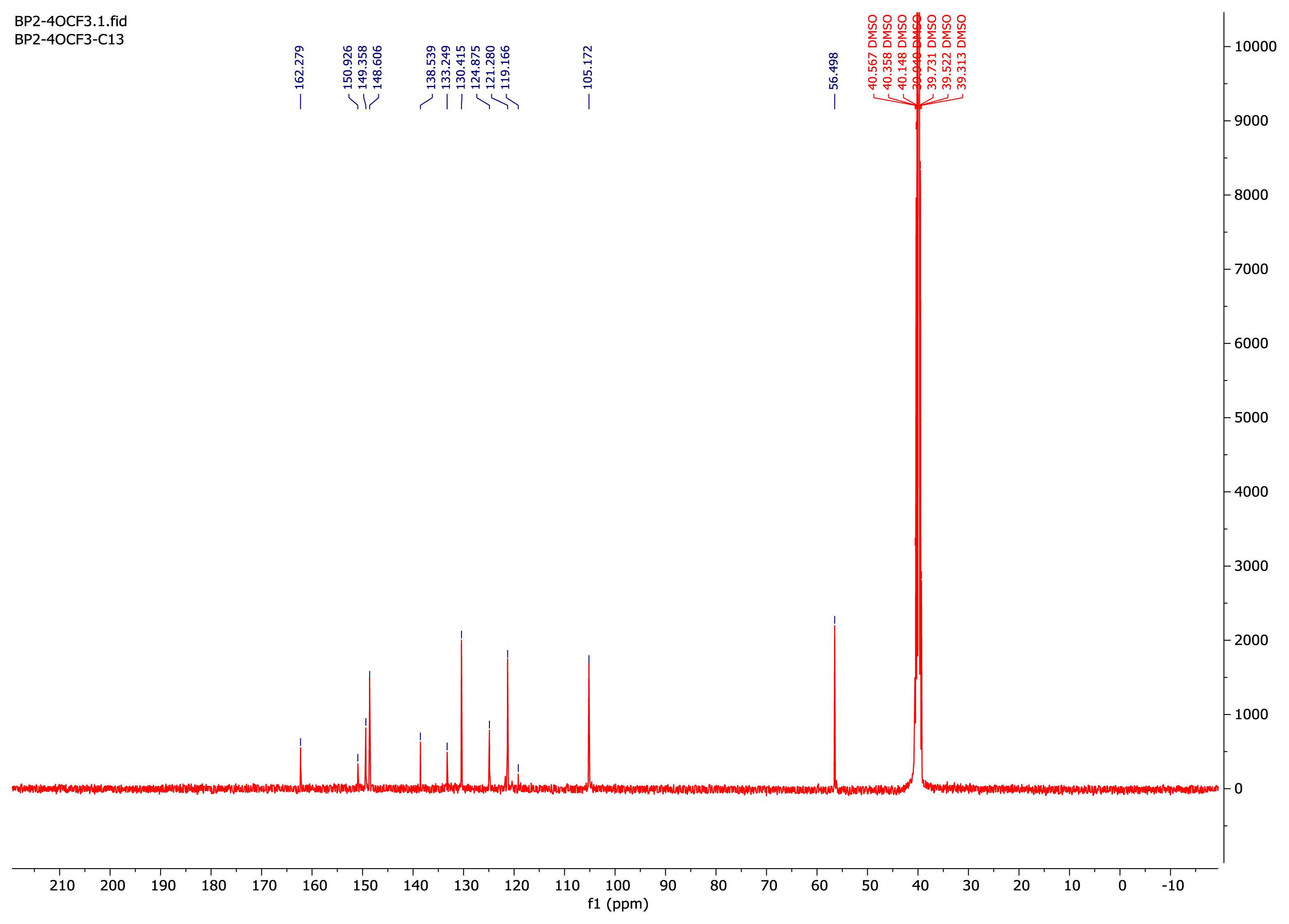

Supplement: Fig. S48 — 13C NMR spectrum of compound 7m [file turkjchem-46-1-236s48.tif]

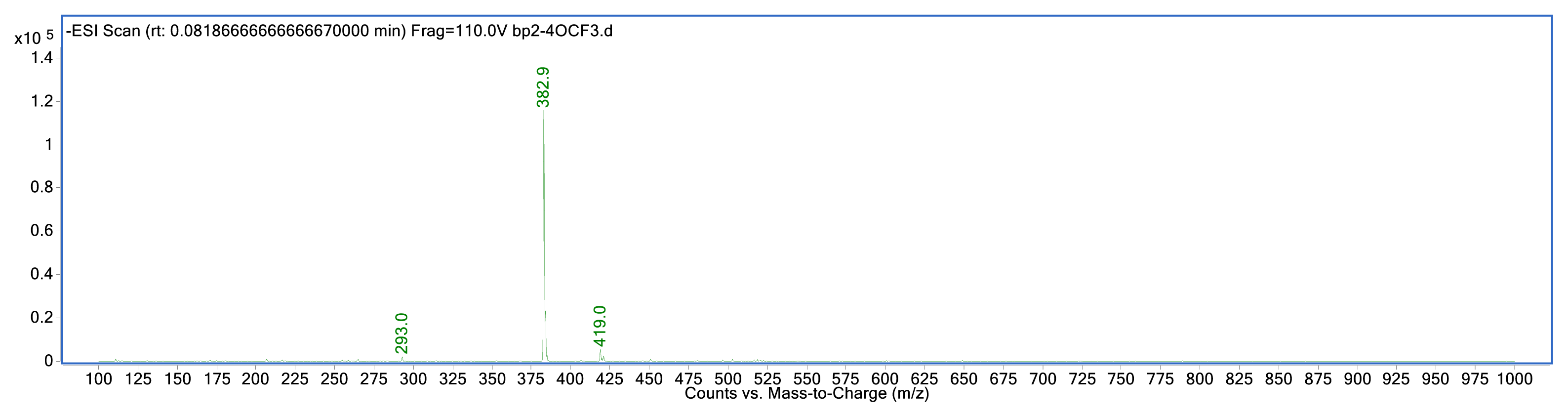

Supplement: Fig. S49 — LC-MS/MS spectrum of compound 7m [file turkjchem-46-1-236s49.tif]
